# Sexually dimorphic control of excitatory synapse maintenance in ventral hippocampal circuits by Neurexin2α ligand Vstm2b

**DOI:** 10.1038/s41467-026-78199-0

**Published:** 2026-10-05

**Authors:** Nicolas Chofflet, Mathilde Chofflet, Mai Inagaki, Anthony John Pastore, Parisa Zarei, Manni Wang, Edan Shpigel, Maia Fukuyama-Kaplan, Martin Resua Rojas, Jeremie Van Prooijen, Ana Novacic, Daham Hettiarachchi, Shanghua Fan, Benjamin Feller, Nayoung Yi, Eric Lecuyer, Gabby Rudenko, Hideto Takahashi

**Affiliations:** 1 Synapse Development and Plasticity Research Unit, Institut de Recherches Cliniques de Montréal, Montreal, QC Canada; 2 Integrated Program in Neuroscience, McGill University, Montreal, QC Canada; 3 UFR Santé, Université de Caen Normandie, Caen, France; 4 Graduate School of Biomedical Sciences, Tokushima University, Tokushima, Japan; 5 Department of Pharmacology and Toxicology, Sealy Center for Structural Biology and Molecular Biophysics, University of Texas Medical Branch, Galveston, TX USA; 6 RNA Biology Research Unit, Institut de Recherches Cliniques de Montreal, Montréal, QC Canada; 7 Department of Medicine, Université de Montréal, Montreal, QC Canada; 8 Division of Experimental Medicine, McGill University, Montreal, QC Canada

**Keywords:** Neuroscience, Molecular neuroscience

## Abstract

Neuronal networks rely on precise synapse formation, maturation, and maintenance orchestrated by synaptic adhesion molecules including α- and β-neurexins (Nrxns). Through unbiased proteomics, we identified Vstm2 family members as α-Nrxn-specific interactors that require inclusion of an insert at alternative splice site 2 (SS2) in α-Nrxns. In particular, as a selective ligand of axonal Nrxn2α^SS2+^, Vstm2b promotes glutamatergic presynaptic assembly. Loss of *Vstm2b* in mice impairs glutamatergic synaptic function and maintenance in the ventral hippocampal CA1 (vCA1), while leaving early synaptogenesis intact. These impairments are sex- and input-specific, highlighting distinct circuit vulnerabilities in male and female mice. Accordingly, *Vstm2b* knockout mice exhibit sexually dimorphic anxiety-related behaviors and social deficits. Our findings establish the Vstm2b-Nrxn2α synaptic adhesion complex as a critical regulator of vCA1 excitatory synapse stability and functional organization and reveal the α-Nrxn-centered interactome as a key driver of sex-dependent synaptic specificity and circuit functions that underlie social behavior and anxiety.

## Abstract (web summary)

This study identifies Vstm2b as a Neurexin2α ligand essential for sex- and input-specific maturation and maintenance of ventral hippocampal excitatory synapses. Loss of *Vstm2b* causes sexually dimorphic social deficits and increased anxiety in mice.

## Introduction

In the central nervous system (CNS), chemical synapses serve as intercellular junctions enabling fast, point-to-point neuronal communication^1^. After the initial contact between pre- and postsynaptic neurons, synapse development unfolds through successive stages of molecular assembly, functional organization, and long-term stabilization, all of which are orchestrated partly by synaptic adhesion molecules (SAMs)^1–3^. As the multitude of SAMs have diverse expression patterns, they have been proposed to form molecular codes that define synapse type, development, and function. In particular, presynaptic neurexins (Nrxns) are essential for synapse development, transmission, and specificity^4,5^. Genetic loss-of-function (LoF) of Nrxns impairs synaptic transmission, plasticity, and structural integrity^4,5^. Furthermore, Nrxn LoF produces context-dependent phenotypes in distinct synapse types, underscoring the essential role of Nrxns in synaptic specificity and diversity in the CNS^4,5^.

In vertebrates, Nrxns are encoded by three genes (*Nrxn1-3*) with expression driven by two promoters producing long α-isoforms (α-Nrxns) and short β-isoforms (β-Nrxns)^4,6^. *Nrxn1* also contains a third promoter expressing the shortest isoform, Nrxn1γ^4,5^. α-Nrxns are comprised of six laminin-neurexin-sex hormone-binding globulin (LNS) domains interspaced with three epidermal-growth-factor-like domains followed by a juxtamembranous stalk, a transmembrane region, and a cytosolic tail^4^. While their transmembrane and cytosolic regions are conserved with α-Nrxns, extracellularly, β-Nrxns feature a unique N-terminal histidine-rich domain followed by a single LNS domain homologous to α-Nrxn LNS6, whereas Nrxn1γ possesses only the stalk^4,6^. Through their extracellular domains, Nrxns bind numerous ligands^4^, including neuroligins (Nlgns)^7,8^, leucine-rich-repeat transmembrane neuronal proteins (LRRTMs)^9–11^, cerebellins (Cblns)^12^, dystroglycan (DAG)^13^, neurexophilins (Nxphs)^14^, and IgSF21^15,16^.

Beyond promoter usage, Nrxns undergo alternative splicing at up to six sites, generating hundreds of isoforms in the mouse and human brain^4–6^. This splicing is cell-type- and region-specific, and it shapes ligand interactions. Inclusion of splice site 4 (SS4) in αLNS6/βLNS domain allows assembly of the Nrxn-Cbln2-GluD1/2 complex, which controls the activity of AMPA and NMDA glutamate receptors (AMPARs and NMDARs)^17–19^, whereas only SS4-insert-lacking isoforms bind LRRTM1/2, forming protein complexes that regulate synaptic transmission and plasticity^4,11^. Thus, Nrxns act as presynaptic hubs with splicing programs dictating ligand specificity and synaptic physiology.

Despite the larger extracellular domain of α-Nrxns compared to β-Nrxns, only a few ligands have been identified and characterised as selective α-Nrxn interactors^20^: Nxphs^14,21^, IgSF21^15,16^, and the calcium channel auxiliary α2δ subunit^22^. Nxphs bind the second LNS (LNS2) domain of α-Nrxns in a splice site 2 (SS2)-dependent manner, regulating inhibitory synaptic transmission and plasticity^21,23,24^. IgSF21 selectively interacts with the LNS1 domain of Nrxn2α to control inhibitory synapse development^15,16^. Nrxn1α also binds α2δ via its LNS1 and LNS5 to inhibit Ca_V_2.2 channel function at neuromuscular junctions in *Caenorhabditis elegans* (*C. elegans*)^22^. In addition to these three α-Nrxn-selective interactors, Hevin has been proposed to bridge Nrxn1α to Nlgn1 for thalamocortical excitatory synapse formation^25,26^, but this remains controversial^27,28^. Hence, no selective α-Nrxn ligands regulating excitatory synapses have been identified, despite strong expression of α-Nrxns in glutamatergic neurons^29^. Importantly, mutations in *α-NRXN-*specific coding regions have been isolated in patients with neurodevelopmental and neuropsychiatric disorders such as autism spectrum disorders (ASD), intellectual disability, or schizophrenia^30^. Therefore, identifying α-Nrxn-specific ligands is critical for understanding synaptic mechanisms in both health and disease^20^.

To this end, this study employed an unbiased proteomic approach and identified V-Set and transmembrane domain containing 2 (Vstm2) family members (Vstm2a, Vstm2b, and Vstm2l) as selective interactors of α-Nrxns, but not β-Nrxns. The interactions require the inclusion of an insert at SS2 within the LNS2 domain of α-Nrxns, establishing LNS2 as a critical hotspot and SS2 splicing as a key regulatory mechanism within the α-Nrxn interactome. Notably, Vstm2b exhibits selective binding to Nrxn2α at an epitope that overlaps with that bound by Nxph1. While Vstm2b can induce glutamatergic presynaptic assembly via axonal Nrxn2α, we show that global deletion of *Vstm2b* does not impair the early formation of glutamatergic synapses but rather diminishes excitatory synaptic function and maintenance in the ventral hippocampus. Importantly, these deficits are sex- and input-dependent. In addition, *Vstm2b* knockout mice display heightened anxiety as well as impaired sociability and social recognition memory in a sex-dependent manner. Thus, our study establishes the Vstm2b-Nrxn2α complex as a regulator of the ventral hippocampal glutamatergic circuits that underlie sexually dimorphic social and anxiety-related behaviors.

## Results

### Identification of Vstm2 family members as α-Nrxn-specific interacting proteins

**Fig. 1:**
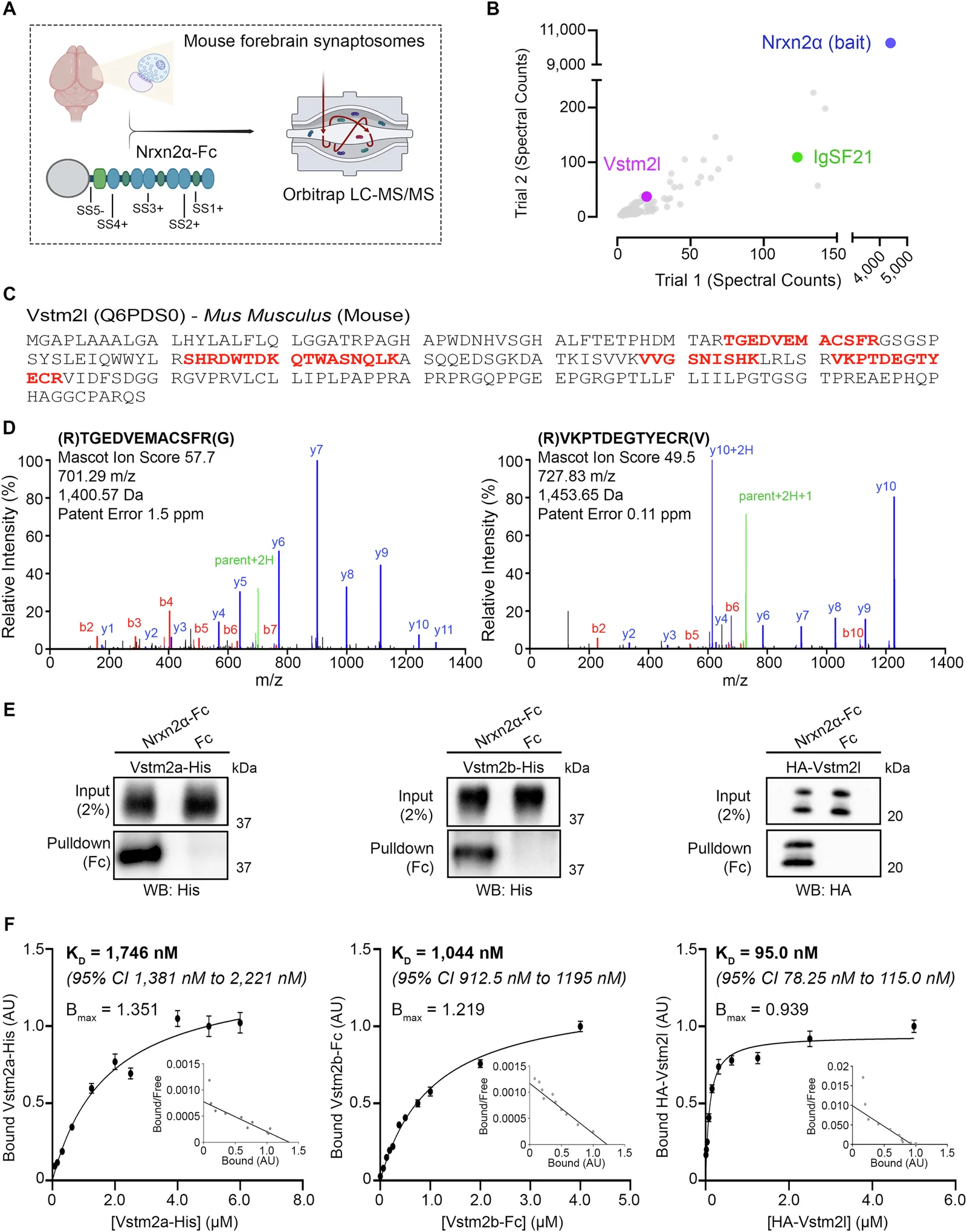
Vstm2a, Vstm2b and Vstm2l are cerebral Nrxn2α interactors. **A** Experimental workflow to isolate mouse Nrxn2α ECD (SS1+, SS2A+, SS3+, SS4+, SS5−)-interacting proteins through affinity-purification coupled to mass-spectrometry. *Created in BioRender. Chofflet, N. (2026)* https://BioRender.com/kpe2yau. **B** Identification of Vstm2l as an Nrxn2α interactor by tandem mass-spectrometry analysis. Following mass-spectrometry and SAINT analysis, candidates with a Bayesian false discovery rate (BFDR) of 0 were selected. The number of spectral counts for selected prey from two independent experiments are shown (two independent experiments, with Fc alone serving as the negative control, for a total of four analyzed samples). **C** Amino acid sequence of the mouse Vstm2l peptides identified by LC-MS/MS following pulldown by Nrxn2α-Fc and bound protein complexes. Residues of identified peptides are shown in red. **D** Spectra of the two Vstm2l peptides with the highest Mascot ion score unique to Nrxn2α-Fc bait obtained by LC-MS/MS. **E** Immunoblots of purified human Vstm2a-His (left), Vstm2b-His (middle) and HA-Vstm2l (right) pulled down by purified human Nrxn2α-Fc (SS1+, SS2A+, SS3−, SS4−, SS5−) but not by Fc alone. **F** Saturation binding of soluble Vstm2a-His (left) and HA-Vstm2l (right) to cell-surface expressed Myc-Nrxn2α (SS1+, SS2A+, SS3+, SS4−, SS5−) and soluble Vstm2b-Fc (middle) to cell-surface expressed HA-Nrxn2α. B_max_ and *K*_D_ values were obtained using a one-site specific binding model and nonlinear regression and averaged between three independent experiments. Scatchard plots were generated by linear regression of the data. Data are shown as mean ± SEM. For Vstm2a-His, *n* = 38, 46, 44, 48, 43, 35, 43, 25, 34, and 29 cells were analyzed at concentrations of 78.125, 156.25, 312.5, 625, 1250, 2000, 2500, 4000, 5000, and 6000 nM, respectively. For Vstm2b-Fc, *n* = 30 cells were analyzed at each indicated concentration. For HA-Vstm2l, the numbers of cells analyzed were *n* = 29, 40, 46, 43, 39, 45, 42, 41, 32, and 39 at concentrations of 9.77, 19.53, 39.06, 78.125, 156.25, 312.5, 625, 1250, 2500 and 5000 nM, respectively. Data were collected from three independent experiments. Source data are provided as a Source data file.

To identify α-Nrxn-interacting proteins, we used recombinant mouse Nrxn2α extracellular domain (ECD) fused to the human immunoglobulin Fc region (Nrxn2α-Fc) as bait in pull-down assays using mouse forebrain synaptosome samples (Fig. 1A). Proteins bound to Nrxn2α-Fc were analyzed by label-free liquid chromatography-tandem mass spectrometry (LC-MS/MS). Among the putative cell-surface proteins identified in two independent experiments was V-set and transmembrane domain containing 2 like protein (Vstm2l) (Fig. 1B–D). The Nrxn2α interactor IgSF21 was also identified, consistent with findings from our previous studies (Fig. 1B)^15,16^.

Vstm2l is a member of the immunoglobulin (Ig) Vstm family composed of 7 members: Vstm1, Vstm2a, Vstm2b, Vstm2l, Vstm3, Vstm4 and Vstm5 (Supplementary Fig. 1A, B). Vstm2l shares the highest homology with its two related brain-expressed isoforms, Vstm2a and Vstm2b (Supplementary Fig. 1A–C). Vstm1, 2a, 2l, 3, and 5 have been studied in the context of immune response and cancer as well as in adipocyte differentiation and metabolism^31–34^. In addition, Vstm5 was found to regulate neuronal dendritic morphology and migration through actin remodeling^35,36^. However, the roles of Vstm2l, Vstm2a, and Vstm2b in synapse and brain development remain unexplored. To validate the LC-MS/MS results, we performed pull-down experiments using purified recombinant soluble hexa-histidine (His)-tagged Vstm2a and Vstm2b (Vstm2a-His and Vstm2b-His), hemagglutinin (HA)-tagged Vstm2l (HA-Vstm2l), and Nrxn2α-Fc. Vstm2a-His, Vstm2b-His, and HA-Vstm2l were pulled down by Nrxn2α-Fc, but not by purified Fc protein (a negative control), indicating that there are direct protein interactions between Vstm2 members and the Nrxn2α ectodomain (Fig. 1E). To further characterize the interactions, we performed in situ binding assays by exposing COS-7 cells expressing full-length Nrxn2α to increasing concentrations of soluble recombinant Vstm2 variants. All of them bound in a concentration-dependent manner fitting a one-site binding model with dissociation constant (*K*_D_) values in the range 95 to 1746 nM (Fig. 1F).

**Fig. 2:**
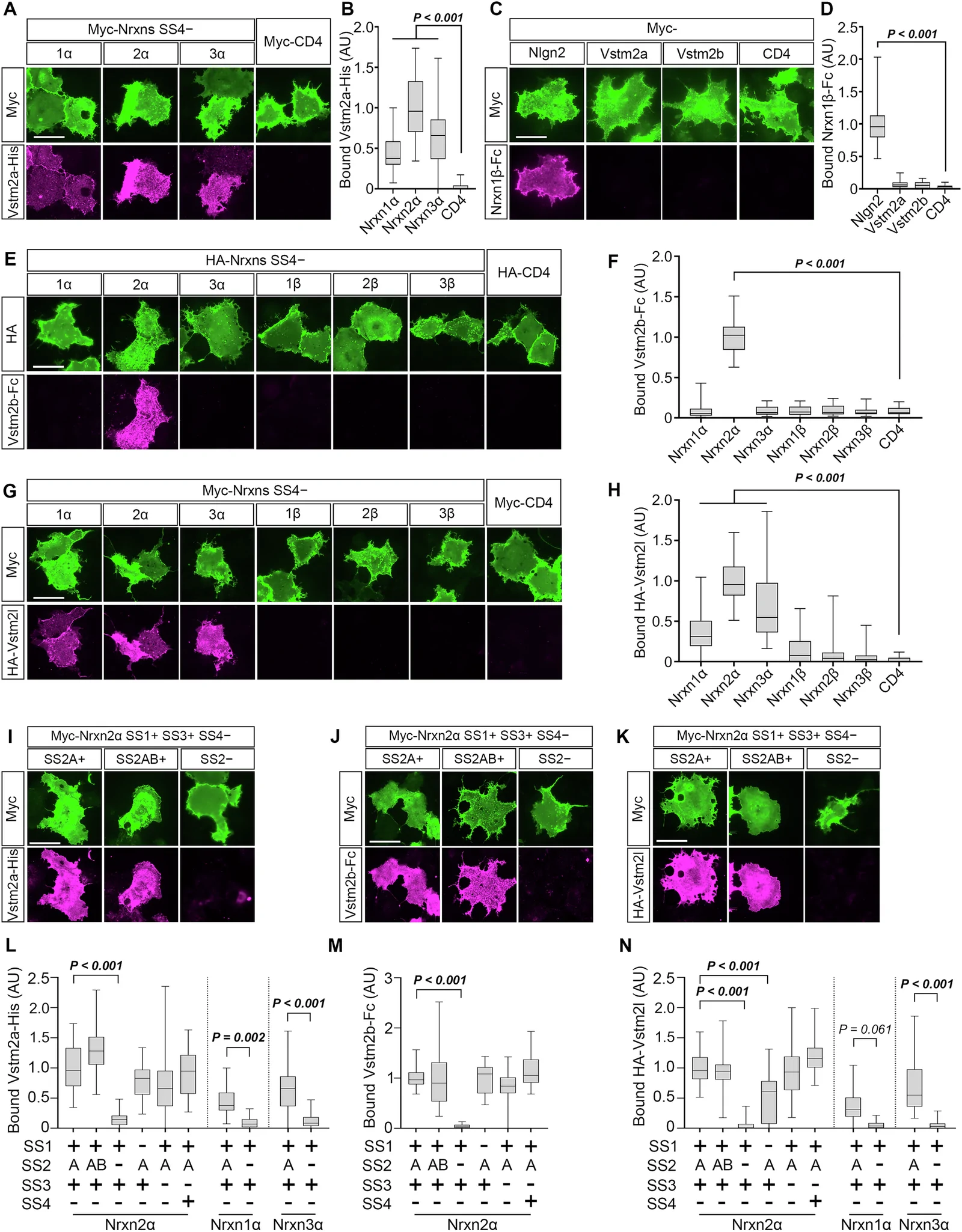
Vstm2a and Vstm2l interact with all three α-Nrxns, while Vstm2b selectively interacts with Nrxn2α. Immunofluorescence images (**A**) and quantification (**B**) of soluble Vstm2a-His bound to various Myc-tagged α-Nrxn isoforms (SS1+, SS2A+, SS3+, SS4−, SS5−). *n* = 20, 20, 18 and 20 cells were analyzed for Nrxn1α, Nrxn2α, Nrxn3α and CD4, respectively. *P* = 0.003 for Nrxn1α and *P* < 0.0001 for Nrxn2α, Nrxn3α compared to CD4. Immunofluorescence images (**C**) and quantification (**D**) of soluble Nrxn1β ^SS4−^-Fc bound to Myc-tagged Nlgn2 A+, Vstm2a, Vstm2b, or CD4. *n* = 54, 49, 29 and 50 cells were analyzed for Nlgn2, Vstm2a, Vstm2b and CD4, respectively. Immunofluorescence images (**E**) and quantification (**F**) of soluble Vstm2b-Fc bound to various HA-tagged Nrxn isoforms (α-Nrxns: SS1+, SS2A+, SS3+, SS4−, SS5−; β-Nrxns: SS4−, SS5−). *n* = 30 cells were analyzed for each construct. Immunofluorescence images (**G**) and quantification (**H**) of soluble HA-Vstm2l bound to various Myc-tagged Nrxn isoforms (α-Nrxns: SS1+, SS2A+, SS3+, SS4−, SS5−; β-Nrxns: SS4−, SS5−). *n* = 30 cells were analyzed for Nrxn1α, Nrxn2α, Nrxn1β, Nrxn2β, Nrxn3β, and CD4, whereas *n* = 36 cells were analyzed for Nrxn3α. Immunofluorescence images of soluble Vstm2a-His (**I**), Vstm2b-Fc (**J**), and HA-Vstm2l (**K**) bound to various alternatively spliced Myc-Nrxn2α SS2 isoforms. Quantification of Vstm2a-His (**L**), Vstm2b-Fc (**M**), and HA-Vstm2l (**N**) bound to various alternatively spliced α-Nrxn isoforms. For Vstm2a, *n* = 33, 39 and 30 cells were analyzed for Nrxn2α SS1+ SS2A+ SS3+ SS4−, Nrxn2α SS1+ SS2AB+ SS3+ SS4−, and all other α-Nrxn constructs, respectively. For Vstm2b, *n* = 29, 55 and 30 cells were analyzed for Nrxn2α SS1+ SS2A+ SS3+ SS4−, Nrxn2α SS1+ SS2AB+ SS3+ SS4− and all other Nrxn2α constructs, respectively. For Vstm2l, *n* = 36, 52 and 30 cells were analyzed for Nrxn2α SS1+ SS2A+ SS3+ SS4−, Nrxn2α SS1+ SS2AB+ SS3+ SS4− and all other α-Nrxn constructs, respectively. Statistical significance was examined by Kruskal–Wallis test with Dunn’s *post hoc* analysis (α = 0.05). Data are presented as medians with interquartile range (IQR) and minimum to maximum values. Data were collected from three independent experiments. Scale bars, 50 μm.

To elucidate whether Vstm2 proteins can interact with other Nrxn isoforms, we performed a series of in situ binding assays in which soluble recombinant Vstm2 variants were applied onto COS-7 cells expressing α- or β-Nrxn1, 2 or 3 (Fig. 2A–H). While Vstm2a-His and HA-Vstm2l bound to all three α-Nrxns, Vstm2b-Fc only interacted with Nrxn2α (Fig. 2B, F, H). Interestingly, no binding of Vstm2b-Fc or HA-Vstm2l to any β-Nrxn isoform was detected (Fig. 2F, H). Due to cross-reactivity of anti-His antibodies with the histidine-rich domain of β-Nrxns^37^, we assessed binding of soluble Nrxn1β-Fc to COS-7 cells expressing myc-tagged Vstm2a or Vstm2b. Soluble Nrxn1β-Fc showed no detectable interaction with Vstm2a, Vstm2b, or CD4 (a negative control), although it robustly bound to Nlgn2, a positive control (Fig. 2C, D). These data indicate that Vstm2 members are α-Nrxn-specific interacting proteins, and that Vstm2b in particular is a Nrxn2α-specific interactor.

### SS2 insert inclusion in α-Nrxns enables their interaction with Vstm2 proteins

To investigate how alternative splicing of α-Nrxns regulates their interactions with Vstm2 members, we conducted in situ binding assays using constructs expressing distinct α-Nrxn splice variants (Fig. 2I–N). Vstm2a, Vstm2b and Vstm2l all bound equally well to Nrxn2α^SS2+^ carrying either the SS2A insert (HAGIGHAM) or the SS2AB insert (HAGIGHAMVNKLHYL) but failed to interact with Nrxn2α^SS2−^, which lacks an SS2 insert (Fig. 2I–N). Similarly, insertion of the SS2A sequence into Nrxn1α and 3α was also required for binding with Vstm2a and 2l (Fig. 2L, N). In contrast, excision of SS1, SS3, or SS4 inserts from Nrxn2α did not markedly alter its interaction with any of the three Vstm2 proteins (Fig. 2L–N). Given that SS2 is located within LNS2 of α-Nrxns, we next investigated whether the LNS2 domain alone was sufficient for binding to Vstm2 members. We found that Vstm2 proteins bound to a membrane-tethered Nrxn2α LNS2 domain construct containing the SS2 insert (LNS2^SS2+^), but not to one lacking the SS2 insert (LNS2^SS2−^) or to Nrxn2α lacking the LNS2 domain (ΔLNS2) (Supplementary Fig. 2A–F). Despite weaker binding of Vstm2b to the isolated LNS2^SS2+^ domain than to full-length Nrxn2α (Supplementary Fig. 2E), domain mapping identified LNS2^SS2+^ as the minimal region that is both necessary and sufficient for Vstm2b binding (Supplementary Fig. 2G, H). Surface plasmon resonance (SPR) analysis revealed concentration-dependent binding of the isolated, hexa-histidine-tagged Ig V-set domain of Vstm2b to Nrxn2α LNS1–egfA–LNS2 containing the SS2 insert (K_D_ = 3.65 ± 0.13 μM), whereas no appreciable binding was detected for the corresponding construct lacking an SS2 insert (Supplementary Fig. 3). Together, these results indicate that SS2 insertion is required for the interaction between Vstm2 members and α-Nrxns.

Finally, because LNS2 contains a calcium (Ca^2+^)-binding site and SS2 insert inclusion greatly diminishes its affinity for Ca^2+^^38^, we tested for Ca^2+^-dependency of Vstm2/α-Nrxn interactions but found that Ca^2+^ depletion did not disrupt their binding (Supplementary Fig. 4A–F). Consistent with the Ca^2+^-chelation experiments, mutation of the conserved LNS2 Ca^2+^-coordinating residue (D335A), predicted to abolish Ca^2+^ binding^39^, did not impair Vstm2b binding and instead significantly enhanced it (Supplementary Fig. 4G, I). Likewise, binding of Neurexophilin-1 (Nxph1), another Ca^2+^-independent LNS2 ligand^21^, was unaffected by the D335A mutation despite exhibiting a distinct preference for the isolated LNS2 domain over full-length Nrxn2α (Supplementary Fig. 4H, J). Together, these findings suggest that Ca^2+^ coordination by LNS2 is not required for Vstm2 binding.

### Vstm2b and Nxph1 share an overlapping binding epitope on Nrxn2α

**Fig. 3:**
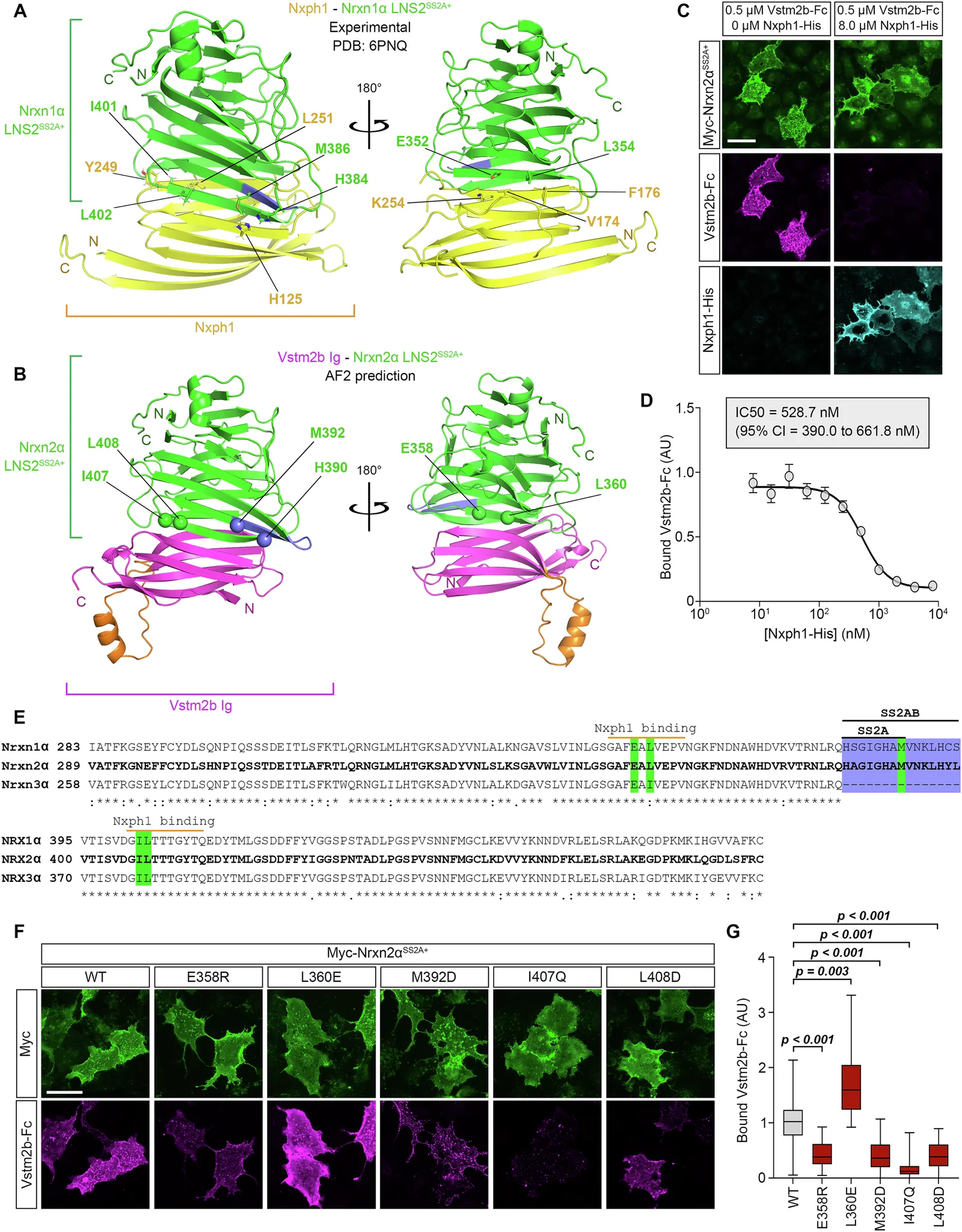
Predicted structure and binding interface of the Vstm2b-Nrxn2α LNS2 complex and competition between Vstm2b and Nxph1 for binding to Nrxn2α. **A** Crystal structure of Nxph1 (yellow) in complex with the Nrxn1α LNS2 domain (green) (PDB: 6PNQ; ref. ^21^). The SS2A splice insert is shown in blue. Residues of interest at the binding interface are labeled as sticks. **B** ColabFold prediction of Vstm2b (magenta) in complex with the Nrxn2α LNS2 domain (green). The SS2A splice insert is shown in blue. Nrxn2α residues selected for mutagenesis are represented as spheres: E^358^ (E^352^), L^360^ (L^354^), M^392^ (M^386^), I^407^ (I^401^), and L^408^ (L^402^), with corresponding Nrxn1α residues indicated in parentheses. A large loop in Vstm2b, with a low pLDDT score, is predicted to be flexible, and the loop is represented in orange. **C** Immunofluorescence images of soluble Vstm2b-Fc (magenta) bound to the surface of cells expressing Myc-Nrxn2α^SS2A+^ (green) in the presence or absence of soluble Nxph1-His (cyan). **D** Quantification of Vstm2b-Fc bound to Myc-Nrxn2α^SS2A+^ in the presence of increasing concentrations of soluble Nxph1-His (0–10,000 nM). *n* = 52, 50, 58, 46, 63, 45, 56, 60, 50, 40, 48 and 44 cells were analyzed at concentrations of 0, 7.8125, 15.625, 31.25, 62.5, 125, 250, 500, 1000, 2000, 4000 and 8000 nM, respectively. **E** Sequence alignment of the LNS2 domains of Nrxn1α (UniProt ID: Q9CS84), Nrxn2α (UniProt ID: E9Q7X7), and Nrxn3α (UniProt ID: Q6P9K9). All Nrxn3α isoforms found on UniProt lack SS2 inserts. The SS2 inserts A and AB are highlighted in teal. Residues selected for mutagenesis are highlighted in green. Key Nxph1-binding regions are indicated with a bar. The degree of conservation is designated underneath as follows: * conserved;  semi-conserved; partially conserved. Immunofluorescence images (**F**) and quantification (**G**) of soluble Vstm2b-Fc bound to Myc-Nrxn2α^SS2+^ WT or mutants expressed on COS-7 cells. *n* = 76, 40, 44, 71, 41 and 38 cells were analyzed for WT, E358R, L360E, M392D, I407Q and L408D, respectively. Statistical significance was examined by Kruskal–Wallis test with Dunn’s *post hoc* analysis (α = 0.05). Data are shown as mean ± SEM (**D**) or as medians with interquartile range (IQR) and minimum to maximum values (**G**). Data were collected from three independent experiments. Scale bars, 50 μm.

Nxph1 binds to the LNS2 domain of α-Nrxns, with the presence of splice inserts (A or AB) at the SS2 site enhancing affinity^21^. The crystal structure of the Nxph1/Nrxn1α LNS2^SS2A+^ complex reveals a β-sheet dimer with edge-to-edge interactions between the β-sheets of Nxph1 and Nrxn1α LNS2 (Fig. 3A)^21^.

Given that Vstm2b also binds the isolated Nrxn2α LNS2^SS2+^ (Supplementary Fig. 2), we hypothesized that the Nrxn2α LNS2^SS2A+^/Vstm2b complex may resemble that of Nrxn1α/Nxph1. Indeed, ColabFold, a variation of AlphaFold2 (AF2), predicted that the Nrxn2α LNS2^SS2A+^/Vstm2b complex has a similar extended β-sheet assembly as observed in the Nrxn1α LNS2^SS2A+^/Nxph1 complex despite clear structural differences between the “β-sandwich” of Nxph1 and the predicted immunoglobulin-like (Ig-like) domain of Vstm2b (Fig. 3A, B and Supplementary Fig. 5A, B). The predicted model for Nrxn2α LNS2^SS2A+^ closely matched the experimentally determined structure of Nrxn1α LNS2^SS2A+^. Competitive binding assays confirmed that soluble His-tagged Nxph1 blocked the interaction of soluble Vstm2b-Fc with Nrxn2α expressed on the cell surface in a concentration-dependent manner (Fig. 3C, D), suggesting that Nxph1 and Vstm2b share an overlapping binding site on the Nrxn2α LNS2 domain.

The crystal structure of the Nrxn1α-LNS2^SS2A+^/Nxph1 complex (PDB: 6PNQ; ref. ^21^) indicates that I401 of Nrxn1α (analogous to I407 in Nrxn2α) inserts into a hydrophobic pocket created primarily by Y249 and L251 of Nxph1, forming a prominent feature at one end of the β-dimer interface (Fig. 3A). L402 (analogous to L408 in Nrxn2α) is also located on the same end of the interface, but this residue is largely solvent-exposed and points to the opposite side of the β-strand compared to I401 (Fig. 3A, B, E). On the other end of the β-dimer, E352 in Nrxn1α (E358 in Nrxn2α) is solvent-exposed but near K254 of Nxph1, suggesting an ionic interaction, while L354 of Nrxn1α (L360 in Nrxn2α) packs between the Nxph1 residues V174 and F176, forming hydrophobic interactions (Fig. 3A, B, E). The residue M386 (M392 in Nrxn2α) located in the SS2A insert forms hydrophobic interactions with H125 located on β-strand β1 of Nxph1 at the surface of the interface between the two proteins (Fig. 3A). Based on these analyses, and the fact that Nxph1 competes with Vstm2b for binding to Nrxn2α, we designed the following mutants in Nrxn2α: E358R to flip the charge and disrupt any potential ion pairs, as well as L360E and M392D to bury a negative charge against potential hydrophobic surfaces. In addition, I407Q was designed to disrupt any buried hydrophobic packing, and L408D was designed to disrupt the hydrophobic surface patch of the Nrxn2α LNS2 domain.

Our cell-surface binding assays using our panel of Nrxn2α mutants demonstrated that I407Q disrupted Vstm2b binding the most (75% loss of binding), followed by E358R, M392D, and L408D (~50% loss of binding) (Fig. 3F, G). Interestingly, L360E enhanced Vstm2b binding. All of the mutants showed comparable surface expression levels (Supplementary Fig. 5C). These results suggest that Vstm2b engages a binding interface of LNS2^SS2A+^ which overlaps with that recognized by Nxph1, enabling Nxph1 to competitively displace Vstm2b. AF2 did not yield a high-confidence prediction for the 3D structure of Vstm2b or its complex with Nrxn2α. Thus, the molecular basis of the binding specificity between Vstm2b and Nrxn2α will require experimental structure determination of the Vstm2b-Nrxn2α protein complex.

### Vstm2a and Vstm2b are GPI-anchored membrane proteins

**Fig. 4:**
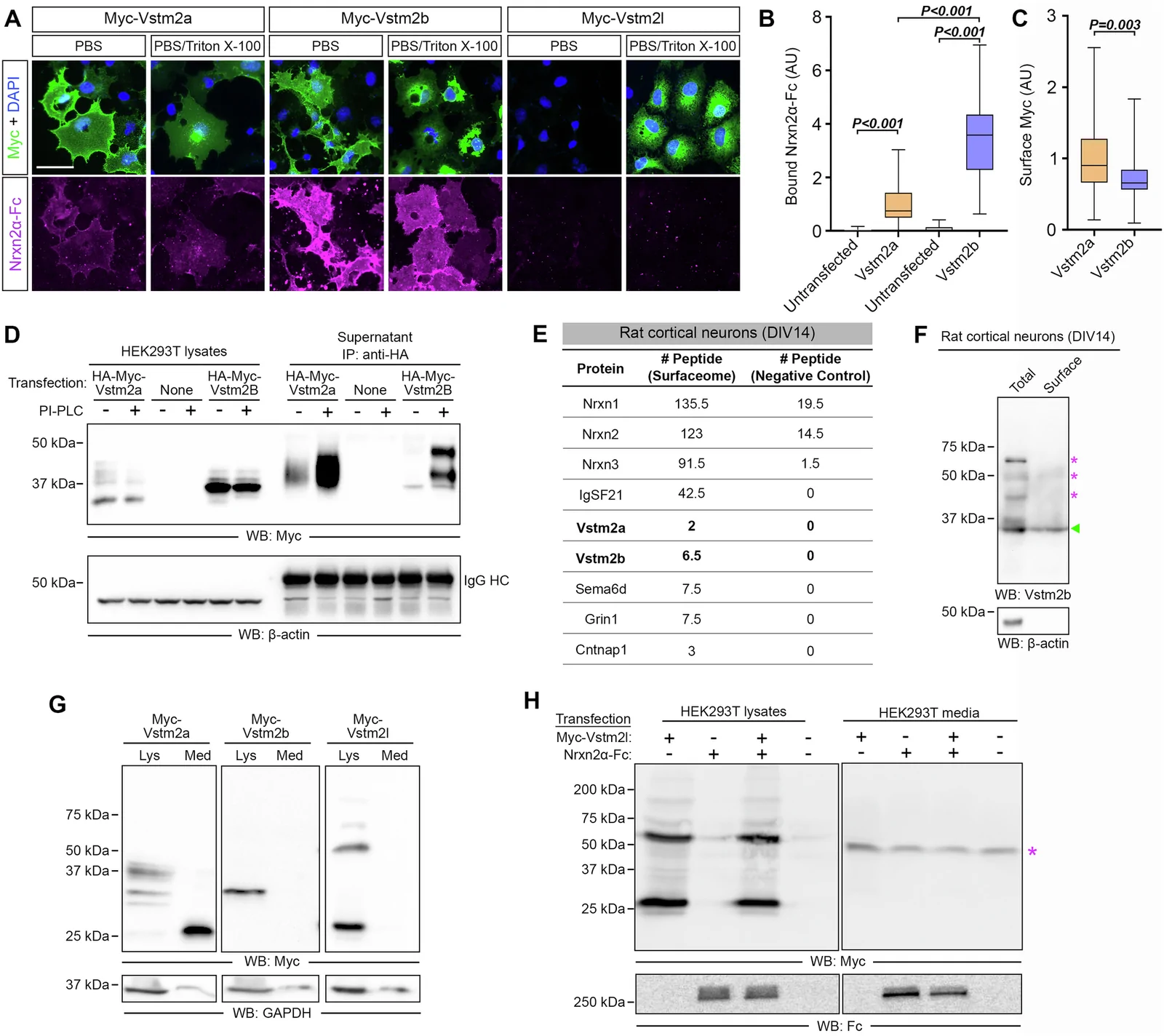
Vstm2a and Vstm2b are GPI-anchored membrane proteins. **A** Immunofluorescence images of COS-7 cells expressing Myc-tagged Vstm2a, Vstm2b or Vstm2l (green) incubated with soluble Nrxn2α-Fc (SS1+, SS2A+, SS3+, SS4+, SS5−; magenta). Cells were stained with or without plasma membrane permeabilization. Scale bar, 50 μm. **B** Quantification of Nrxn2α-Fc bound to the transfected cells expressing Myc-Vstm2a or Myc-Vstm2b, and to neighboring untransfected cells, as assessed by Myc-positive/negative and DAPI-positive regions. For Vstm2a, *n* = 35 untransfected and 36 transfected cells; for Vstm2b, *n* = 53 untransfected and 53 transfected cells. **C** Quantification of cell-surface expression of extracellularly Myc-tagged Vstm2a and Vstm2b. Data are presented as medians with interquartile range (IQR) and minimum to maximum values (**B**, **C**). *n* = 36 and 53 transfected cells were analyzed for Vstm2a and Vstm2b, respectively. Statistical significance was examined by Kruskal–Wallis test with Dunn’s *post hoc* analysis (**B**) or two-tailed Mann–Whitney test (**C**) (α = 0.05). *n* ≥ 35 cells from two independent experiments. **D** Immunoblots of cell lysates and culture medium from HEK293T cells transfected with HA-Myc–tagged Vstm2a or HA-Myc–Vstm2b, or left untransfected, in the presence or absence of PI-PLC treatment. β-actin was used as a loading control. **E** Exclusive spectrum counts of Vstm2a, Vstm2b, and selected cell adhesion molecules from the cortical neuron surfaceome (one independent experiment, with two independent culture wells serving as biological replicates for a total for four analyzed samples). The negative control, processed without biotin, accounts for non-specific binding and background signal. **F** Immunoblots of rat cortical neuron lysates and biotinylated cell-surface proteins, probed with anti-Vstm2b antibodies. The green arrowhead indicates endogenous Vstm2b; magenta asterisks indicate non-specific bands. β-actin was used as a loading control. **G** Immunoblots of lysates and culture medium from HEK293T cells expressing Myc-tagged Vstm2a, Vstm2b, or Vstm2l. Lys: lysates, Med: culture medium. GAPDH was used as a loading control. **H** Immunoblots of lysates and culture medium from HEK293T cells expressing Myc-Vstm2l and Nrxn2α^SS2A+^-Fc alone or together. The magenta asterisk indicates non-specific bands. Similar results were obtained in three independent experiments for (**D**, **G**) and one independent experiment for (**F**, **H**). Source data are provided as a Source data file.

All Vstm2 members contain a putative N-terminal signal peptide (Supplementary Fig. 1C). Vstm2a and Vstm2b are also predicted to be glycosylphosphatidylinositol (GPI)-anchored, while Vstm2l is not (Supplementary Fig. 1C). In agreement with this, when exogenously expressed in COS-7 cells, both Vstm2a and Vstm2b, but not Vstm2l, were displayed on the cell surface, where they bound Fc-tagged Nrxn2α ECD (Fig. 4A). Nrxn2α-Fc bound to Vstm2a- and Vstm2b-expressing cells, but not to neighboring untransfected cells, with stronger binding observed for Vstm2b (Fig. 4B) despite its slightly lower surface expression (Fig. 4C), consistent with their relative binding affinities (Fig. 1F). Treatment with a phosphatidylinositol-specific phospholipase C (PI-PLC), which specifically cleaves GPI anchors, released Vstm2a and Vstm2b into the cell supernatants (Fig. 4D), confirming that they are GPI-anchored membrane proteins. Consistently, in cell surface biotinylation assays, endogenous Vstm2a and Vstm2b, but not Vstm2l, were detected in the surfaceome of cultured cortical neurons together with Nrxns (Fig. 4E), and endogenous Vstm2b was validated as a surface protein in cultured rat cortical neurons by immunoblotting (Fig. 4F). Together, these results establish Vstm2a and Vstm2b as GPI-anchored membrane proteins on the cell surface that can bind the Nrxn2α ECD.

Interestingly, an N-terminal fragment of Vstm2a, but not of Vstm2b or Vstm2l, was released into the culture medium of HEK293T cells with a lower apparent molecular weight (~25 kDa) than the corresponding species in lysates (~40 kDa), perhaps as a result of ectodomain shedding (Fig. 4G). The absence of Vstm2l in the medium suggests that Vstm2l might require association with α-Nrxns for secretion, echoing FAM19A1 secretion, which requires binding the Nrxn1β ECD^40^. However, Vstm2l was also not detected in the media, even when co-expressed with Nrxn2α-Fc (Fig. 4H), suggesting little to no secretion of Vstm2l from HEK293T cells, even in the presence of Nrxn2α. Enzymatic deglycosylation further revealed that both Vstm2a and Vstm2l carry N-linked glycans and that Vstm2a additionally undergoes sialic acid (SA) modification (Supplementary Fig. 6).

### Vstm2b is expressed in CA1 pyramidal neurons in the ventral hippocampus

**Fig. 5:**
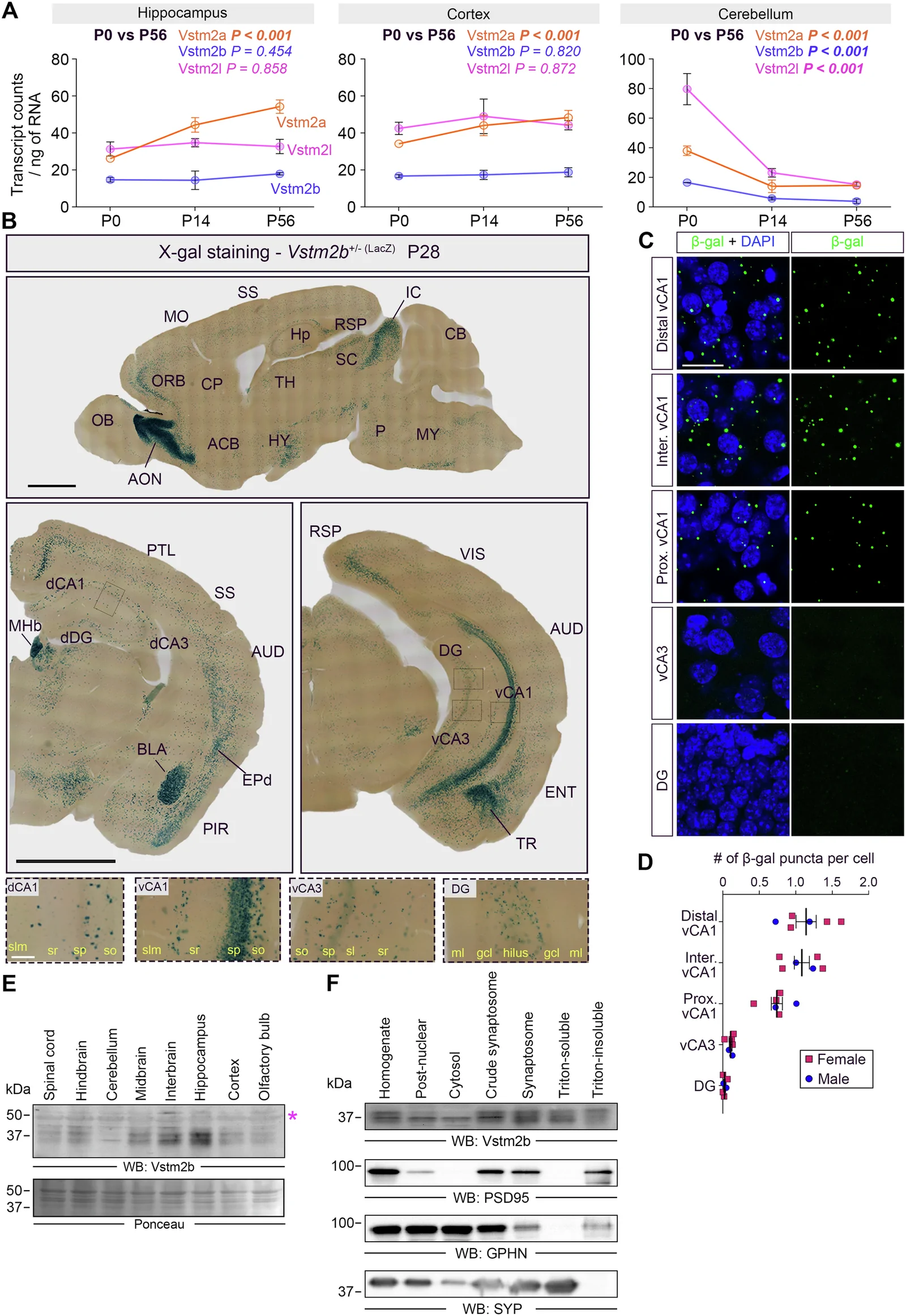
Vstm2b is highly expressed in the CA1 pyramidal cells of the ventral hippocampus. **A** Absolute quantification of Vstm2a, Vstm2b and Vstm2l transcripts by RT-qPCR in the hippocampus, cortex and cerebellum of P0, P14 and P56 mice. Statistical significance was examined by two-way ANOVA with Tukey post hoc analysis (α = 0.05). Data are shown as mean ± SEM. *n* = 3 mice per developmental stage; each sample was analyzed by qPCR in technical triplicate and averaged. **B** X-gal staining of sagittal and coronal sections of *Vstm2b* reporter mouse brains at P28. Dashed boxes indicate the regions from the ventral hippocampus (vCA1, vCA3, and DG) and the dorsal CA1 (dCA1) shown at higher magnification below. Scale bars, 2 mm (black) or 150 μm (white). ACB nucleus accumbens; AON anterior olfactory nucleus, AUD auditory areas, BLA basolateral amygdala, CB cerebellum, CP caudoputamen, ENT entorhinal area, EPd endopiriform nucleus dorsal, Hp hippocampus, HY hypothalamus, IC inferior colliculus, MHb medial habenula, MO somatomotor areas, MY medulla, OB olfactory bulb, ORB orbital area, P pons, PIR piriform area, PTL posterior parietal association areas, RSP retrosplenial cortex, SC superior colliculus, SS somatosensory areas, TH thalamus, TR postpiriform transition area, VIS visual areas, slm *stratum lacunosum-moleculare,* sr *stratum radiatum,* so *stratum oriens,* sp *stratum pyramidale,* sl *stratum lucidum,* gcl *granule cell layer,* ml *molecular layer*. Representative images (**C**) and quantification (**D**) of β-galactosidase immunofluorescence in pyramidal cells in the distal, intermediate, and proximal vCA1 and the vCA3, and DG granule cell layer of *Vstm2b* reporter mice at P28. Scale bar, 20 μm. Data are shown as mean ± SEM; *n* = 6 mice (2 males, 4 females). **E** Immunoblots of anatomically defined CNS regions probed for Vstm2b at P28. The magenta asterisk denotes a non-specific ~50-kDa band that remains detectable in *Vstm2b* KO brain lysate (see Supplementary Fig. 7C). **F** Immunoblots of mouse forebrain fractionations probed for Vstm2b as well as PSD95 and gephyrin (GPHN) and synaptophysin (SYP). The Triton-soluble fractions were enriched for SYP, and the insoluble fractions were enriched for PSD95 and GPHN. Similar results were obtained in three and two independent experiments for (**E**, **F**), respectively. Source data are provided as a Source data file.

We next investigated CNS expression of the Vstm2 family members. Real-time quantitative PCR revealed that Vstm2a, Vstm2b, and Vstm2l mRNA are relatively stably expressed throughout postnatal development in the hippocampus and cortex (Fig. 5A) but sharply downregulated between postnatal day 0 (P0) and P14 in the cerebellum. In all three regions, Vstm2b was consistently less abundant than Vstm2a and Vstm2l.

Given its localization at the plasma membrane and its ability to bind the Nrxn2α ECD with higher affinity than Vstm2a, features typical of synaptic adhesion complexes, we decided to focus on Vstm2b. To further characterize Vstm2b expression in the mouse brain, we generated and analyzed *Vstm2b* reporter mice (*Vstm2b*^tm1a^ line) expressing β-galactosidase (β-gal) under the *Vstm2b* promoter^41^ (*Vstm2b*^+/−(LacZ)^ mice; Supplementary Fig. 7A, B). X-gal staining at P28 revealed enriched expression in the ventral hippocampus, specifically in the ventral CA1 (vCA1) pyramidal layer, with sparse signals in other hippocampal subregions including the dorsal CA1 (dCA1) (Fig. 5B), suggesting that Vstm2b is expressed in vCA1 pyramidal cells as well as in hippocampal GABAergic interneurons. There was also prominent expression in the basolateral amygdala (BLA) and the medial habenula (MHb), as well as in olfactory-related regions, including the anterior olfactory nucleus (AON), the piriform cortex (PIR), the dorsal endopiriform nucleus (EPd), and the postpiriform transition area (TR) (Fig. 5B and Supplementary Fig. 8A). β-gal immunofluorescence further confirmed enrichment in vCA1 pyramidal cells (Fig. 5C, D), and expression in parvalbumin- and calretinin-positive GABAergic interneurons (Supplementary Fig. 8B). These observations are consistent with our analysis of a publicly available single-cell RNA sequencing dataset^42^ (Supplementary Fig. 8C). Together, these results indicate that Vstm2b exhibits a region- and cell-type-dependent expression pattern, with specific enrichment in vCA1 pyramidal cells within the hippocampal formation.

Immunoblotting for Vstm2b also revealed robust enrichment in the hippocampus relative to other brain regions, with again little to no expression detected in the cerebellum at P28 (Fig. 5E). To assess whether Vstm2b localizes to synapses, we performed subcellular fractionation of mouse brains. Vstm2b was detected in synaptosomes and in both Triton-soluble and Triton-insoluble fractions, which correspond to pre- and postsynaptic compartments, respectively, as validated by compartment-specific markers (Triton-soluble: positive for Synaptophysin (SYP), negative for PSD95 and Gephyrin (GPHN); Triton-insoluble: negative for SYP, positive for PSD95 and GPHN) (Fig. 5F). These results indicate that Vstm2b is present in synaptic fractions containing both pre- and postsynaptic markers within the hippocampus. To further examine the subcellular distribution of Vstm2b in neurons, exogenous HA-Vstm2b was expressed in cultured rat hippocampal neurons and visualized by immunofluorescence using an anti-HA antibody. HA-Vstm2b exhibited a punctate distribution along both axonal and dendritic processes (Supplementary Fig. 9A, B). Quantification of the dendritic-to-axonal polarity index revealed no preferential enrichment in either compartment at DIV14 or DIV21 (Supplementary Fig. 9C). These findings indicate that Vstm2b is broadly distributed across neuronal processes, consistent with its expression in both pre- and postsynaptic compartments as observed for endogenous Vstm2b in mouse brain synaptic fractions (Fig. 5F).

### Vstm2b induces glutamatergic presynaptic assembly via axonal Nrxn2α

**Fig. 6:**
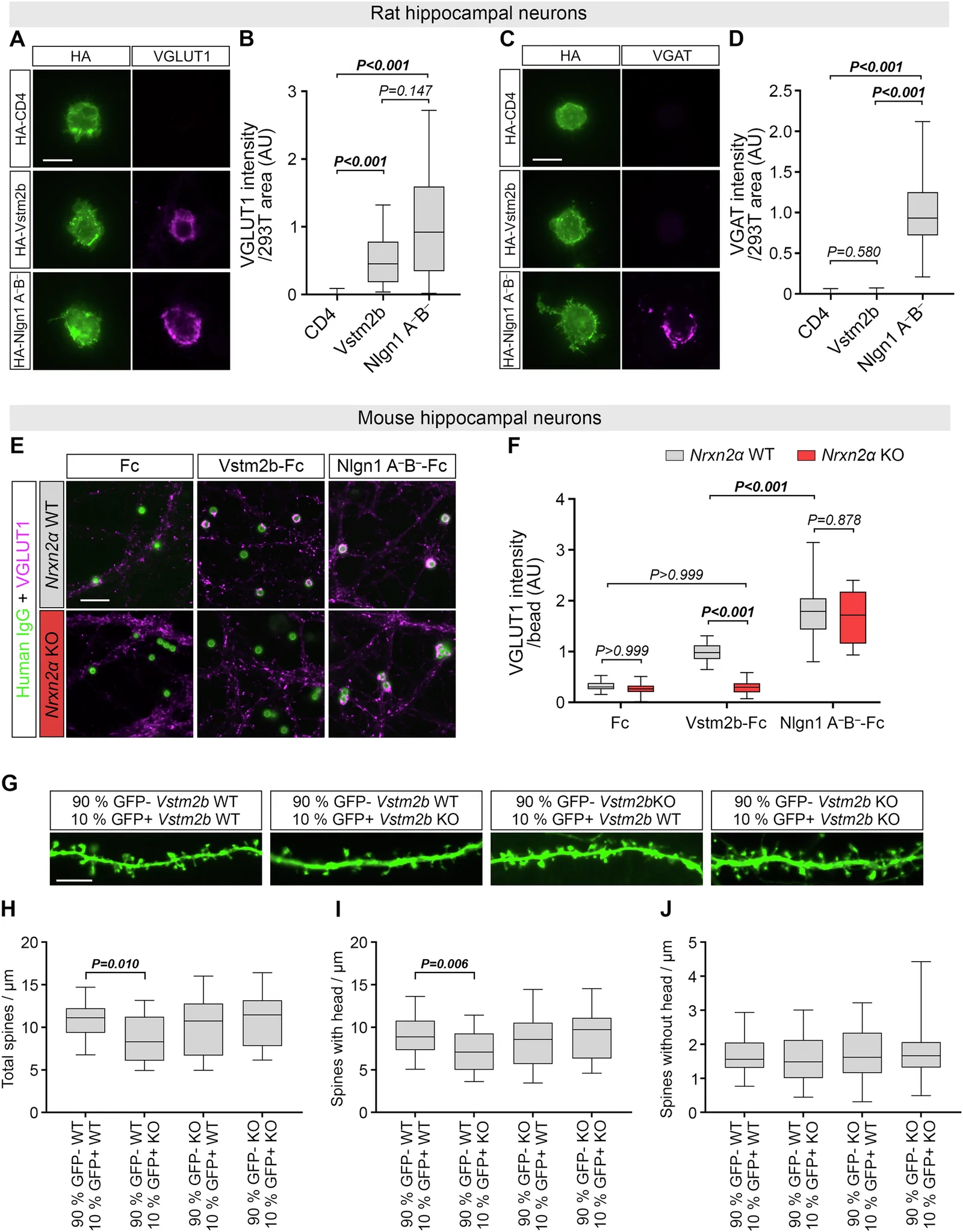
Vstm2b induces glutamatergic, but not GABAergic, presynaptic assembly through Nrxn2α, and endogenous postsynaptic Vstm2b regulates dendritic spine density. Representative immunofluorescence images of VGLUT1 (**A**) and VGAT (**C**) around HEK293T cells expressing HA-CD4, HA-Vstm2b, or HA-Nlgn1 A^−^B^−^ cocultured with rat hippocampal neurons. Scale bars, 15 μm. Quantification of VGLUT1 (**B**) or VGAT (**D**) accumulated on HEK293T cells in (**A**, **C**). Statistical significance was examined with Kruskal–Wallis tests with Dunn’s *post hoc* analysis (α = 0.05). Data are presented as medians with interquartile range (IQR) and minimum to maximum values. For both VGLUT1 and VGAT, *n* = 34, 34 and 36 cells were analyzed for CD4, Vstm2b and Nlgn1, respectively. Data were collected from three independent experiments for hippocampal neurons. **E** Representative fluorescence images of VGLUT1 (magenta) clustering induced by Fc- Vstm2b-Fc or Nlgn1 A^−^B^−^-Fc-coated beads (green) applied to mouse hippocampal neurons derived from *Nrxn2α* wild-type (WT) and knockout (KO) littermates. Scale bars, 10 μm. **F** Quantification of VGLUT1 accumulated on beads in (**E**). Statistical significance was examined with a two-way ANOVA with Sidak post hoc analysis (α = 0.05). Data are presented as medians with interquartile range (IQR) and minimum to maximum values. *n* = 30 images with 10 beads averaged per image for each indicated condition from three independent experiments. **G** Representative images of dendritic segments from mixed mouse hippocampal neuron cultures containing 90% GFP-negative (GFP-) cells and 10% GFP-expressing (GFP+) cells of the indicated *Vstm2b* genotypes. Scale bar, 5 μm. Quantification of total spine density (**H**), spines with heads (**I**), and spines without heads (**J**) per 10 μm of dendrite under the indicated culture conditions. Statistical significance was examined by one-way ANOVA with Tukey’s post hoc test (α = 0.05). Comparisons were made to the 90% GFP- WT/10% GFP + WT culture condition. Only significant *P* values are shown. Data are presented as medians with interquartile range (IQR) and minimum to maximum values. *n* = 27 dendritic segments for 90% GFP- WT/10% GFP + WT, 33 dendritic segments for 90% GFP- WT/10% GFP+ *Vstm2b* KO, 24 dendritic segments for 90% GFP- *Vstm2b* KO/10% GFP + WT, and 30 dendritic segments for 90% GFP- *Vstm2b* KO/10% GFP+ *Vstm2b* KO. Data were obtained from two independently prepared neuronal cultures.

Given that several postsynaptic membrane proteins, including Nlgns and LRRTMs, promote presynaptic assembly through transcellular interactions with presynaptic Nrxns^4^, we next tested whether Vstm2b exhibits similar activity using heterologous synapse formation assays. We co-cultured HEK293T cells expressing HA-Vstm2b, HA-Nlgn1 A^−^B^−^, or a negative control (HA-CD4) with rat hippocampal neuron cultures and assessed the clustering of glutamatergic and GABAergic presynaptic markers, VGLUT1 and VGAT, respectively (Fig. 6A–D). Vstm2b induced robust VGLUT1 clustering, but not VGAT clustering, around transfected HEK293T cells compared to a negative control, CD4, while Nlgn1 A^−^B^−^ induced both (Fig. 6B, D). These results indicate that Vstm2b selectively promotes glutamatergic, but not GABAergic, presynaptic assembly.

To determine if Vstm2b-mediated presynaptic assembly requires its trans-interaction with endogenous axonal Nrxn2α, we performed heterologous synapse formation assays in which Vstm2b-Fc-coated beads were applied to hippocampal neurons isolated from *Nrxn2α* WT or knockout (KO) littermate embryos (Fig. 6E, F). Consistent with the HEK cell-based assays, Vstm2b-Fc-coated beads induced a significant accumulation of VGLUT1, but not VGAT, around the beads compared with negative control Fc-coated beads (Fig. 6E, F for VGLUT1; Supplementary Fig. 10 for VGAT), confirming that Vstm2b selectively promotes excitatory, but not inhibitory, presynaptic assembly. Notably, VGLUT1 clustering around Vstm2b-Fc-coated beads was significantly dampened in *Nrxn2α* KO neurons to levels comparable to the clustering induced by Fc-coated controls (Fig. 6F). In contrast, VGLUT1 clustering induced by Nlgn1 A^−^B^−^-Fc-coated beads was unaltered in *Nrxn2α* KO neurons (Fig. 6F), consistent with a previous study^16^. Similarly, VGLUT1 clustering was significantly reduced around Vstm2b-expressing HEK293T cells in *Nrxn2α* KO cortical neurons (Supplementary Fig. 11). To formally identify endogenous Nrxn2α as a presynaptic receptor for Vstm2b, we incubated rat hippocampal neurons with Vstm2b-Fc-coated beads, cross-linked bead-associated protein complexes with DTSSP, and analyzed them by label-free LC-MS/MS (Supplementary Fig. 12A, B). Consistent with our in situ binding assay results, we isolated Nrxn2, but not Nrxn1 or Nrxn3 (Supplementary Fig. 12C–F), and did not detect Nrxn2β-specific peptides. Together, these results indicate that endogenous Nrxn2α acts as a presynaptic functional receptor that mediates Vstm2b-induced excitatory presynaptic assembly through a trans-interaction.

### Postsynaptic Vstm2b regulates dendritic spine density

The ability of Vstm2b to induce glutamatergic presynaptic assembly through a trans-interaction with axonal Nrxn2α suggested that postsynaptic Vstm2b is involved in excitatory synapse development. To test this, we developed a strategy to isolate pre- and post-synaptic effects of *Vstm2b* loss. Specifically, we generated and validated a *Vstm2b*^tm1d^ global KO mouse line (Supplementary Fig. 7A–D) and established mixed hippocampal neuron cultures, in which a small number of GFP-labeled (GFP+) *Vstm2b* WT neurons or GFP+ homozygous *Vstm2b* KO neurons (10%) were surrounded by an excess of unlabeled *Vstm2b* WT neurons or unlabeled homozygous *Vstm2b* KO neurons (90%). We then assessed dendritic spine density in the GFP+ neurons in these mixed cultures (Fig. 6G). GFP+ *Vstm2b* KO neurons surrounded by *Vstm2b* WT neurons (i.e. post-synaptic *Vstm2b* loss) exhibited a significant reduction in both total spine density and the density of head-positive spines compared with GFP+ *Vstm2b* WT neurons surrounded by *Vstm2b* WT neurons (Fig. 6H, I). In contrast, GFP+ *Vstm2b* WT neurons surrounded by *Vstm2b* KO neurons (i.e. pre-synaptic Vstm2b loss) displayed spine densities comparable to those of GFP+ *Vstm2b* WT neurons surrounded by *Vstm2b* WT neurons (Fig. 6H, I). Thus, postsynaptic, rather than presynaptic, Vstm2b is critical for excitatory synapse development. Interestingly, GFP+ *Vstm2b* KO neurons surrounded by *Vstm2b* KO neurons did not show changes in either total spine density or head-positive spine density (Fig. 6H, I), suggesting that the relative level of postsynaptic Vstm2b expression compared with neighboring neurons determines the impact of Vstm2b loss on dendritic spine density. This observation is consistent with transcellular competitive processes previously reported in studies of sparse Nlgn1 knockdown in the cortex and in *Nlgn1* KO neurons cocultured with WT neurons^43^. Finally, the density of headless spines, presumed precursors of mature dendritic spines, remained unchanged across all conditions (Fig. 6J), suggesting that Vstm2b primarily regulates the maturation and/or maintenance of excitatory synapses rather than their initial formation.

### SS2 splicing of α-Nrxns is cell- and gene-dependent, with SS2-positive Nrxn2α predominating in glutamatergic neurons

To gain insights into how alternative splicing of Nrxn2α at SS2 contributes to synaptic connectivity through Vstm2b, we examined the cell-type specific pattern of SS2 splicing in the mouse hippocampus and neocortex by analyzing a published dataset mapping ribosome-associated transcript isoforms in genetically defined neuron types^44^ (Supplementary Fig. 13). In both the neocortex and the hippocampus, glutamatergic neurons predominantly express SS2-insert-containing Nrxn2α isoforms, whereas GABAergic neurons, particularly in the hippocampus, exhibit lower SS2 inclusion (~25%) (Supplementary Fig. 13B, E, H). Notably, hippocampal CA3 pyramidal neurons (Grik4+), which project to the CA1 via Schaffer collaterals (SC), display 90% SS2 inclusion in Nrxn2α transcripts (Supplementary Fig. 13E). However, the percent spliced-in (PSI) of SS2 differs markedly across the three α-Nrxn genes. In Nrxn1α, far fewer molecules contained an SS2 insert (Supplementary Fig. 13A, D, G), and Nrxn3α was almost exclusively expressed as SS2-lacking isoforms in all cell types (Supplementary Fig. 13C, F, I). Together with the predominant expression of Vstm2b in vCA1 pyramidal cells and its absence in vCA3 pyramidal cells (Fig. 5B–D), these results are consistent with a model in which Vstm2b engages in a trans-interaction with presynaptic Nrxn2α carrying the SS2 insert to organize SC-CA1 glutamatergic synapses in the ventral hippocampus.

### *Vstm2b* is required for transmission and maintenance, but not early formation, of excitatory synapses in ventral CA1 circuits in a sex- and input-specific manner

To analyze the role of endogenous Vstm2b on glutamatergic synapses in vivo, we comprehensively characterized a *Vstm2b*^tm1d^ global KO mouse line (Supplementary Fig. 7A–C). Immunoblotting confirmed the complete loss of endogenous Vstm2b protein (Supplementary Fig. 7C). We found no deviation of litter genotypes from Mendelian distribution, no gross change in body or brain morphology, and no drastic change in body weight in the KO animals (Supplementary Fig. 7D–H).

**Fig. 7:**
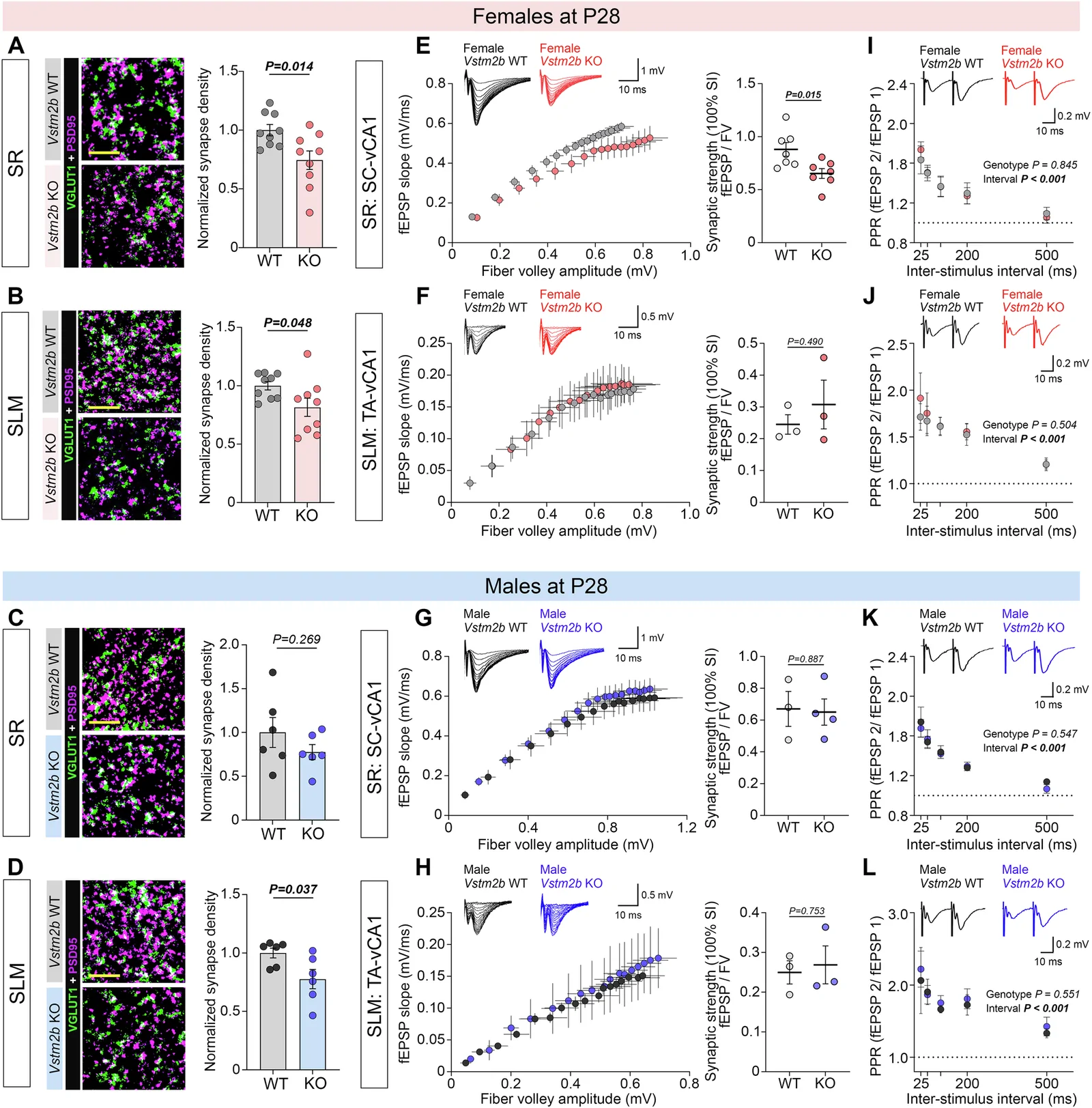
Loss of *Vstm2b* diminishes hippocampal glutamatergic synapse density and basal synaptic transmission in ventral hippocampal CA1 circuits of 1-month-old mice in a sex- and input-specific manner. High-magnification immunofluorescence images (left) and quantification (right graph) of VGLUT1–PSD95 colocalization puncta, corresponding to glutamatergic synapses, in the vCA1 SR and SLM of *Vstm2b* WT and KO females (**A** for SR and **B** for SLM) and males (**C** for SR and **D** for SLM) at P28. Scale bars, 5 μm. Statistical significance was determined by unpaired two-tailed *t*-tests (α = 0.05). Data are shown as mean ± SEM, and each point represents the averaged result from one mouse; *n* = 9 female WT mice; *n* = 9 female KO mice; *n* = 6 male WT mice; *n* = 6 male KO mice. Input/output relationship shown as fEPSP slope versus fiber volley (FV) amplitudes elicited in the SR and SLM of *Vstm2b* WT and KO females (**E** for SR and **F** for SLM) and males (**G** for SR and **H** for SLM) at P28. Inset: representative fEPSPs elicited with stepwise increases in stimulation intensity (scale bars, 1.0 mV for SR and 0.5 mV for SLM, 10 ms). Right graphs: synaptic strength assessed as fEPSP plotted against FV amplitude at maximum stimulation intensity in each condition. Statistical significance was determined by unpaired two-tailed *t*-tests (α = 0.05). Data are shown as mean ± SEM, and each point represents the averaged result from one mouse. Paired-pulse ratio (PPR) between the first and second fEPSPs at different inter-stimulus intervals in *Vstm2b* WT and KO females (**I** for SR and **J** for SLM) and males (**K** for SR and **L** for SLM) at P28. Data are shown as mean ± SEM. Statistical significance was assessed by two-way RM ANOVA (α = 0.05). Insets: Representative PPR trace at a 25 ms inter-stimulus interval (scale bars, 0.2 mV, 10 ms). Sample numbers for the SC-vCA1 synapses (**E**, **G**, **I**, **K**)**:** *n* = 7 mice (24 recordings) for female WT; *n* = 7 mice (25 recordings) for female KO; *n* = 3 mice (12 recordings) for male WT; *n* = 4 mice (17 recordings) for male KO. Sample numbers for the TA-vCA1 synapses (**F**, **H**, **J**, **L**): *n* = 3 mice (9 recordings) for female WT; *n* = 3 mice (9 recordings) for female KO; *n* = 3 mice (12 recordings) for male WT; *n* = 3 mice (11 recordings) for male KO.

To test whether endogenous Vstm2b was required for the structural organization of glutamatergic synapses, we assessed excitatory synaptic markers in the vCA1 of *Vstm2b* WT and KO littermates at P28. Low-magnification images showed no obvious changes in VGLUT1 and PSD95 immunoreactivity or laminar architecture (Supplementary Fig. 14). However, in high-magnification images, a significant decrease in glutamatergic synapse density, as indicated by VGLUT1–PSD95 colocalization puncta, was detected in the *stratum radiatum* (SR) of female, but not male, KO mice (Fig. 7A, C). In contrast, synapse density in the *stratum lacunosum-molecular*e (SLM) was significantly reduced in both female and male KO mice compared to sex-matched WT littermates (Fig. 7B, D). The reduction in VGLUT1–PSD95 colocalization puncta was not accompanied by changes in the density or area of VGLUT1 or PSD95 single puncta **(**Supplementary Fig. 15**)**, indicating a specific disruption in synaptic connectivity rather than a general decrease in synaptic protein content. Importantly, the density of GABAergic synapses assessed by VGAT-GPHN colocalization puncta was unchanged between genotypes in both sexes in the *stratum pyramidale* (SP), SR and SLM of vCA1 (Supplementary Fig. 16). Thus, *Vstm2b* deletion results in a sex- and input-dependent reduction of excitatory synapse density in the ventral CA1 at P28.

Next, we examined the role of Vstm2b in glutamatergic synaptic transmission by measuring evoked field excitatory postsynaptic potentials (fEPSPs) in the intermediate SR, where ventral CA3 axons form synapses onto vCA1 dendrites (Fig. 7 and Supplementary Fig. 17A–D, F–I). Consistent with the structural phenotypes (Fig. 7A, C), loss of *Vstm2b* resulted in a significant decrease in input/output (I/O) responses of SC-vCA1 synapses, as revealed by plotting fEPSP slope against fiber volley (FV) amplitude, in female, but not male, mice (Fig. 7E, G). In contrast, comparable I/O responses between genotypes were observed in both sexes in the SLM, where temporoammonic (TA) inputs from the entorhinal cortex (EC) innervate distal dendrites of the vCA1 (Fig. 7F, H), despite the significant reduction of excitatory synapse density in this layer (Fig. 7B, D). We further assessed paired-pulse facilitation (PPF), a form of presynaptic short-term plasticity, and found that PPF of both SC-vCA1 and TA-vCA1 synapses was comparable between WT and KO littermates in both sexes (Fig. 7I–L). These results indicate that the observed I/O deficit of SC-vCA1 synapses in KO females is not due to altered neurotransmitter release probability and further suggest that presynaptic terminals of both SC-vCA1 and TA-vCA1 synapses may remain functionally intact in KO mice. Thus, the female-specific reduction of excitatory synapse density in the SR is associated with reduced strength of SC-vCA1 synapses, whereas the decrease in synapse density within the SLM is not accompanied by reduced strength of TA–vCA1 inputs in either sex.

**Fig. 8:**
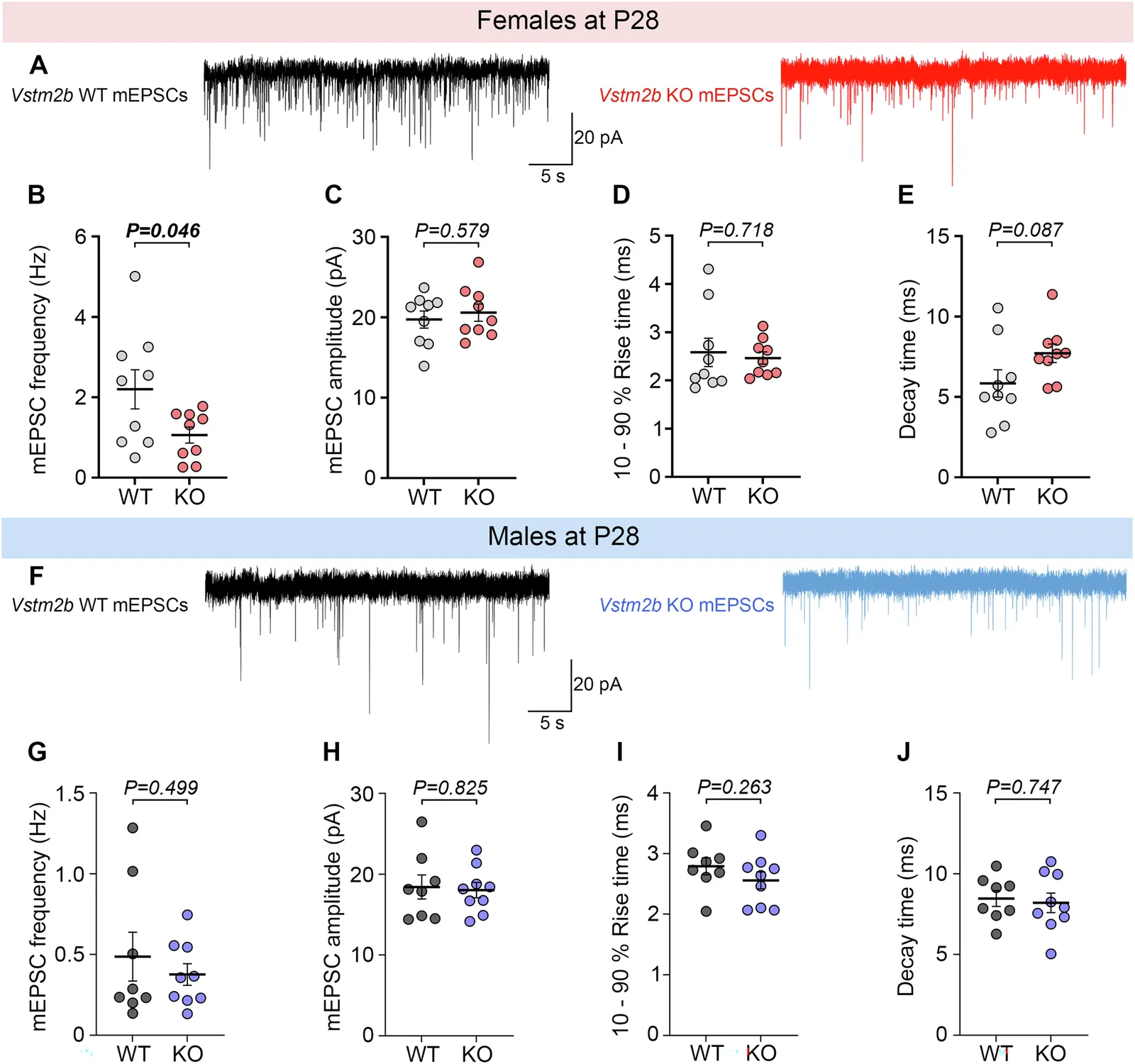
Loss of *Vstm2b* reduces miniature excitatory synaptic transmission in vCA1 pyramidal neurons of female mice, but not male mice. Representative mEPSC recordings from ventral hippocampal CA1 pyramidal neurons of *Vstm2b* WT and KO females (**A**) and males (**F**) at P28. Scale bars, 20 pA, 5 s. Quantification of mEPSC frequency in female (**B**) and male (**G**) mice. Quantification of mEPSC amplitude in female (**C**) and male (**H**) mice. Quantification of mEPSC 10–90% rise time in female (**D**) and male (**I**) mice. Quantification of mEPSC decay time in female (**E**) and male (**J**) mice. Statistical significance was determined for (**B**–**E**, **G**–**J)** by unpaired two-tailed *t*-tests (α = 0.05). Data are shown as mean ± SEM, and each point represents one recorded cell. Sample numbers: *n* = 9 cells from 6 female WT mice; *n* = 9 cells from 6 female KO mice; *n* = 8 cells from 5 male WT mice; *n* = 9 cells from 5 male KO mice.

To further assess excitatory synaptic function, we recorded miniature excitatory postsynaptic currents (mEPSCs) from vCA1 pyramidal neurons at P28 (Fig. 8). Consistent with the reduced excitatory synapse density and impaired SC-vCA1 basal synaptic transmission, female *Vstm2b* KO mice exhibited a significant reduction in mEPSC frequency compared to WT littermates, whereas mEPSC amplitude and kinetics were unchanged (Fig. 8A–E). In contrast, mEPSC frequency, amplitude, and kinetics were comparable between WT and KO males (Fig. 8F–J), despite the reduced excitatory synapse density observed in the SLM, suggesting that the loss of distal SLM synapses may have a limited impact on somatically recorded mEPSCs. Together, these results indicate that loss of *Vstm2b* reduces spontaneous excitatory synaptic transmission selectively in female mice through a decrease in functional synapse number without altering postsynaptic functional properties. Notably, WT females displayed a higher mEPSC frequency and faster decay time than WT males (Supplementary Fig. 18A, D), whereas amplitude and rise time were similar between sexes (Supplementary Fig. 18B, C), indicating intrinsic sex-dependent differences in excitatory synaptic properties in vCA1. Importantly, mEPSC frequency and decay time were comparable between sexes in *Vstm2b* KO condition (Supplementary Fig. 18A, D). These results indicate that *Vstm2b* KO diminishes the intrinsic sex differences, further supporting sex-dependent regulation of excitatory synaptic function by *Vstm2b*.

**Fig. 9:**
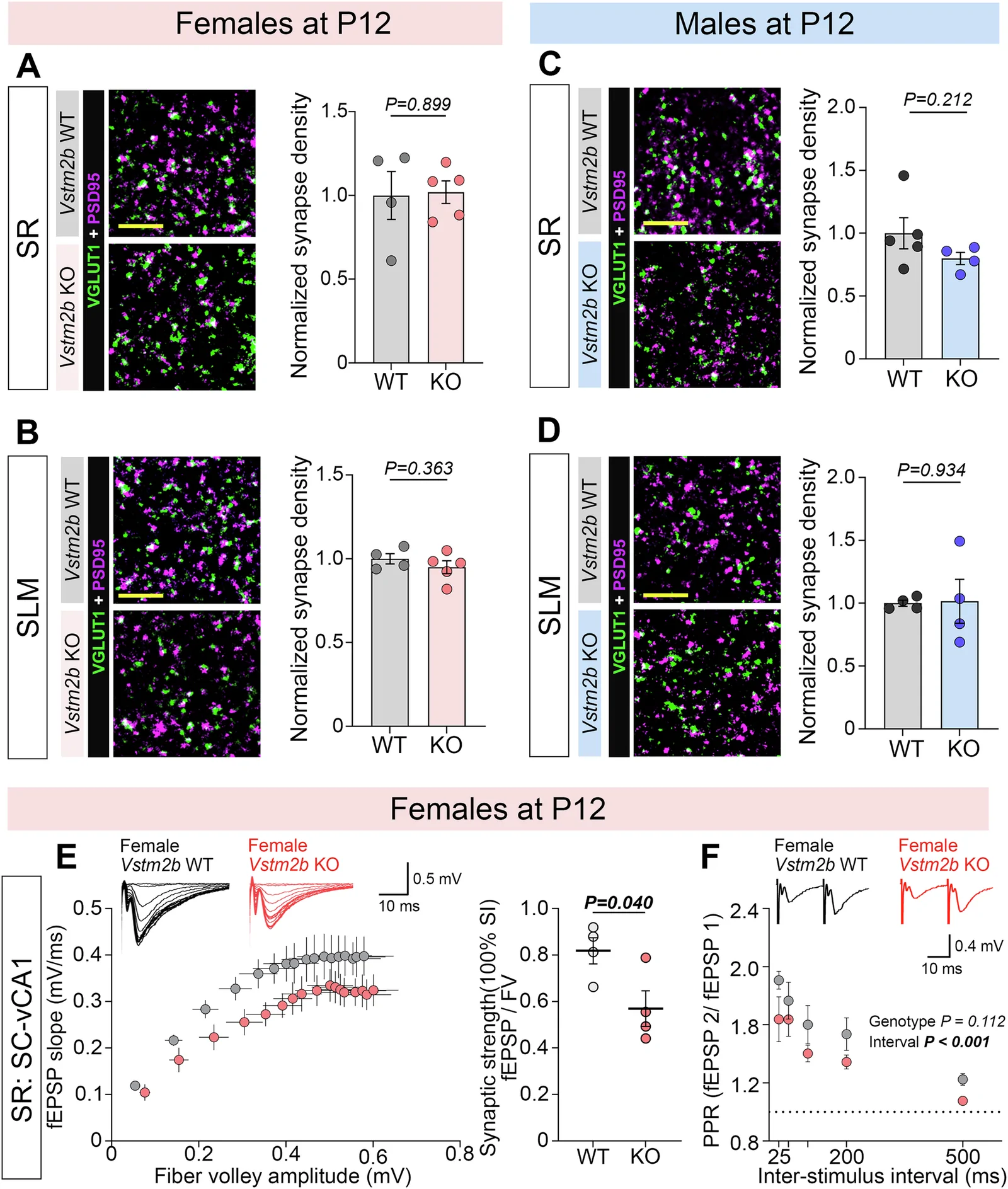
Loss of *Vstm2b* alters excitatory synaptic transmission, but not density, in the vCA1 SR of females during the peak period of synaptogenesis. High-magnification immunofluorescence images (left) and quantification (right graph) of VGLUT1–PSD95 colocalization puncta, corresponding to glutamatergic synapses, in the vCA1 of *Vstm2b* WT and KO females (**A** for SR and **B** for SLM) and males (**C** for SR and **D** for SLM) at P12. Scale bars, 5 μm. Statistical significance was determined by unpaired two-tailed *t*-tests (α = 0.05). Data are shown as mean ± SEM, and each point represents the averaged result from one mouse; *n* = 4 and 5 female WT and KO mice (**A**, **B**); *n* = 5 and 4 male WT and KO mice (**C**, **D**), respectively. **E** Input/output relationship shown as fEPSP slope versus FV amplitude elicited in the SR of *Vstm2b* WT and KO female mice at P12. Inset: representative fEPSPs in the vCA1 SR elicited with stepwise increases in stimulation intensity (scale bars, 0.5 mV, 10 ms). Right graph: synaptic strength assessed as fEPSP plotted against FV amplitude at maximum stimulation intensity. Statistical significance was determined by unpaired *t*-tests (α = 0.05). Data are shown as mean ± SEM, and each point represents the averaged result from one mouse. **F** Paired-pulse ratio (PPR) between the first and second fEPSPs at different inter-stimulus intervals in *Vstm2b* WT and KO mice at P12. Data are shown as mean ± SEM. Statistical significance was assessed by two-way RM ANOVA (α = 0.05). Insets: representative PPR trace at a 25 ms inter-stimulus interval (scale bars, 0.4 mV, 10 ms). Sample numbers for (**E**, **F**): *n* = 4 mice (15 recordings) for female WT; *n* = 4 mice (16 recordings) for female KO.

To determine whether the reduced synapse density in the *Vstm2b* KO vCA1 results from impaired synaptogenesis or defects in synapse maintenance, we examined mice at P12 (Fig. 9), a developmental stage corresponding to the peak of synaptogenesis^45^. At this stage, the density of glutamatergic synapses (VGLUT1/PSD95 colocalization puncta) as well as the density and area of VGLUT1 and PSD-95 single puncta in the vCA1 SR and SLM were comparable between KO and WT littermates in both sexes (Fig. 9A–D and Supplementary Fig. 19). Strikingly, however, the synaptic strength of SC-vCA1 synapses in female KO mice was significantly reduced, whereas PPF remained unchanged (Fig. 9E, F and Supplementary Fig. 17E, J). These results suggest that impaired synaptic transmission precedes - and likely drives - the subsequent reduction in synapse density in the SR of *Vstm2b* KO female mice, consistent with defective synapse maintenance. Together, these findings indicate that Vstm2b is required for excitatory synapse function and maintenance, rather than for early synaptogenesis, within ventral hippocampal circuits.

### Loss of *Vstm2b* leads to sex-dependent heightened anxiety and impaired social responses

**Fig. 10:**
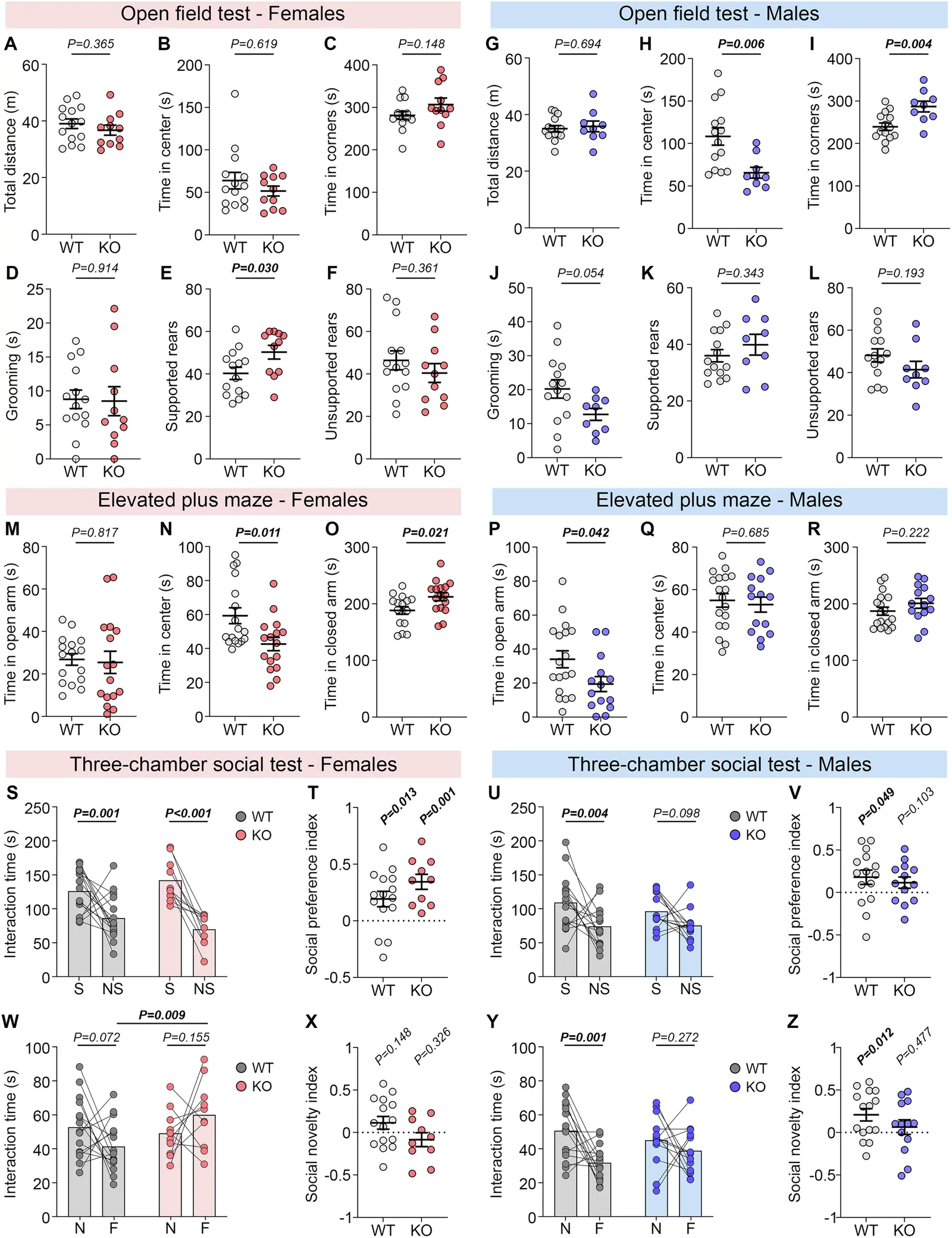
Deletion of *Vstm2b* in mice induces sexually dimorphic anxiety-like behaviors and social impairments. **A**–**L** Open field test. Total distance moved (**A**, **G**). Time spent in the center zone (**B**, **H**). Time spent in the corners of the arena (**C**, **I**). Time spent self-grooming (**D**, **J**). Number of supported rearing events (**E**, **K**). Number of unsupported rearing events (**F**, **L**). **M**–**R** Elevated plus maze. Time spent in the open arms (**M**, **P**). Time spent in the center zone of the maze (**N**, **Q**). Time spent in the closed arms (**O**, **R**). **S**–**Z** Three-chamber sociability and social novelty test (3C). Time spent investigating social and non-social stimulus (**S**, **U**). Sociability index (**T**, **V**). A sociability index >0 means that the mouse spent more time investigating the social stimulus than the non-social one. Time spent investigating a novel and a familiar conspecific (**W**, **Y**). Social novelty index (**X**, **Z**). A social novelty index >0 means that the mouse spent more time investigating the novel conspecific than the familiar one. S social, NS non-social, N novel, F familiar. Statistical significance was examined by unpaired two-tailed *t*-tests (**A**–**R**), two-way ANOVA with post hoc Fisher’s least significant difference (LSD) (**S**, **U**, **W**, **Y**), or one-sample *t*-tests (two-tailed) against 0 as hypothetical mean (**T**, **V**, **X**, and **Z**) (α = 0.05). Sample number: *n* = 14 (OFT), 16 (EPM), 15 (3C) mice for female WT; 11 (OFT), 16 (EPM), 10 (3C) mice for female KO; *n* = 14 (OFT), 18 (EPM), 15 (3C) mice for male WT, and 9 (OFT), 14 (EPM), 13 (3C) mice for male KO. Data are shown as mean ± SEM.

Given that the ventral hippocampus is implicated in anxiety and social memory^46,47^, we analyzed the impact of *Vstm2b* deletion on anxiety and social behaviors. In an open field test (OFT), WT and KO mice in both sexes showed comparable locomotion (Fig. 10A, G). However, KO males, but not females, spent significantly less time in the center zone and more in the corners than sex-matched WT controls (Fig. 10B, C, H, I), indicating increased thigmotaxis. As self-grooming and rearing are linked to stress and anxiety^48,49^, we measured these behaviors in the OFT. KO males tended to groom less, while KO females showed significantly more supported rearing than WT controls (Fig. 10D–F, J–L), suggesting sex-dependent exploratory alterations, associated with increased caution and anxiety-related responses in some contexts^50^. In the elevated plus maze, female KO mice spent equivalent time as WT controls in open arms but significantly less in the center and more in closed arms (Fig. 10M–O). Male KO mice spent significantly less time in the open arms than WT controls (Fig. 10P–R). In a light-dark box test, we did not detect any differences in time spent or the latency to first entrance in the lit compartment (Supplementary Fig. 20A–D). Altogether, these results suggest that loss of *Vstm2b* elevates anxiety, particularly in males.

We next analyzed whether loss of *Vstm2b* affects social behavior using the three-chamber interaction test. Female KO mice exhibited normal social preference, spending significantly more time investigating an age- and sex-matched conspecific than a non-social stimulus (an empty cage) (Fig. 10S, T). In contrast, KO males failed to prefer a conspecific, suggesting impaired sociability (Fig. 10U, V). During the social novelty phase, WT females tended to prefer a novel conspecific over a familiar one (Fig. 10W, X). However, KO females did not show any preference for social novelty, spending significantly more time with the familiar mouse than did WT controls (Fig. 10W, X). KO males exhibited similar novelty impairment (Fig. 10Y, Z). No deficits were detected in spatial working memory or associative memory, assessed by Y-maze and contextual fear conditioning tests (Supplementary Fig. 20E–N). Together, these results suggest that Vstm2b KO males are impaired in both sociability and social recognition memory, whereas KO females show deficits restricted to social recognition memory.

## Discussion

Studies on Vstm2 members remain scarce, and no previous study has described their roles in synapse biology. Our proteomic profiling of the Nrxn2α ECD interactome revealed Vstm2a, 2b, and 2l as α-Nrxn-specific ligands, with interaction requiring inclusion of an insert at SS2 within the LNS2 domain. Vstm2b selectively interacts with Nrxn2α^SS2+^ via a binding site that overlaps with that of Nxph1, resulting in direct binding competition. SS2 insert inclusion is highly regulated in a gene- and cell-type-specific manner, with Nrxn2α expressed almost exclusively as SS2-insert-containing isoforms in hippocampal glutamatergic neurons. While Vstm2b promotes glutamatergic presynaptic assembly through trans-interaction with axonal Nrxn2α, *Vstm2b* deletion impairs glutamatergic synaptic function and maintenance rather than early synaptogenesis in the ventral hippocampal CA1. Importantly, these effects are sex- and input-specific, with distinct vCA1 pathways affected in male and female mice. Correspondingly, *Vstm2b* KO mice exhibit sex-dependent anxiety-related behaviors and deficits in sociability and social recognition memory. Together, these findings indicate that the Vstm2b-Nrxn2α complex emerges as an important regulator of ventral hippocampal glutamatergic circuits and may contribute to sexually dimorphic synapse specificity and circuit function.

During early development, an excess of nascent synapses is generated, followed by activity-dependent refinement that stabilizes active synapses while eliminating those that are less active, ensuring proper circuit maturation^51^. Constitutive deletion of Nrxns or their ligands typically impairs synapse function and integrity without profoundly affecting synapse formation^4^. For instance, global deletion of Nlgn1 does not diminish excitatory synapse density in hippocampal or cortical pyramidal neurons^43^. Instead, Nlgn1 regulates activity-dependent spinogenesis and synapse number through an intercellular competitive process^43^, and both Nlgns and LRRTMs prevent activity-dependent synapse elimination in vitro^52^. Thus, Nlgn1 controls synapse density in a context- and activity-dependent manner rather than acting as a master regulator of early synaptogenesis. We found a similar intercellular competitive process for Vstm2b: deletion of *Vstm2b* reduced the density of mature dendritic spines in KO neurons cocultured with an excess of WT neurons, whereas the density was unaffected in KO-KO co-cultures. Furthermore, we show that global *Vstm2b* KO reduces synapse density at P28, during a critical period of hippocampal circuit maturation, but not at P12, when early synaptogenesis is occurring, suggesting that Vstm2b, like Nlgn1, is not essential for initial synapse formation. Notably, in the SR of females, *Vstm2b* KO leads to functional impairments that precede synapse loss, implying that Vstm2b deficiency may facilitate the removal of less active synapses and/or destabilize active ones. To further clarify how Vstm2b regulates synapse density, conditional deletion of *Vstm2b* in the hippocampus before, during, and after synaptogenesis will be important.

While the reduction in synapse density in the SR correlates with decreased SC-vCA1 synaptic strength in *Vstm2b* KO female mice, this correlation is not observed for TA-vCA1 inputs in the SLM of either sex. Although data from the ventral CA1 are limited, studies in the dorsal CA1 indicate that the SR and SLM differ markedly: the SLM has fewer excitatory and relatively more inhibitory synapses than the SR^53^, and although perforated synapses are more prevalent, AMPAR expression is lower^53,54^. In addition, excitatory synapses in the SLM exhibit lower baseline release probability than their SR counterparts, in part due to a reduced contribution of N-type voltage-gated Ca^2+^ channels to neurotransmitter release; however, this N-type channel contribution can be enhanced during synaptic potentiation through NMDAR- and L-type Ca^2+^ channel–dependent mechanisms^55^, indicating that TA synapses retain a substantial capacity for activity-dependent strengthening. Consistently, TA inputs also exhibit input-specific homeostatic plasticity: while lesions of EC neurons reduce excitatory synapse density in the SLM, both lesions and chemogenetic silencing of these neurons increase spontaneous synaptic currents in the CA1, an effect not seen following SC lesions^56^. Similarly, amino-oxyacetic acid (AOAA)-induced partial neuronal loss in EC layer III does not significantly reduce TA–CA1 efficacy, suggesting that even a modest number of intact synapses can sustain physiologically normal fEPSPs^57^. Together, these findings suggest that the homeostatic properties of TA-CA1 synapses may account for the absence of an fEPSP phenotype in *Vstm2b* KO mice, despite a significant reduction in the number of excitatory synapses in the SLM. Future studies such as pharmacological inhibition of N-type Ca^2+^ channels during SLM fEPSP recordings could help clarify the mechanisms underlying the apparent dissociation between reduced synapse density and preserved basal synaptic transmission in *Vstm2b* KO mice.

In both vertebrates and invertebrates, sexual dimorphism can be imprinted in the nervous system through sex-specific differences in synaptic connectivity. For instance, in *C. elegans*, sex-specific pruning maintains distinct subsets of synapses, establishing sexually dimorphic networks^58^. In mice, Nrxn3 contributes to sex-specific GABAergic synaptic connectivity within the ventral subiculum^59^. We found that *Vstm2b* deletion in mice impairs vCA1 glutamatergic synapse maintenance in both the SR and SLM in females, but only in the SLM in males. On the other hand, the mechanisms underlying sex-dependent maturation and maintenance of the SC-CA1 and TA-CA1 synapses remain unknown. Interestingly, astrocyte-released Humanin (Hn) plays a critical role in synapse maintenance and function, with its secretion regulated by ovarian hormones^60^. Moreover, Vstm2l acts as an Hn ligand and modulates its activity^61^, leading to the intriguing hypothesis that Vstm2b may also interact with Hn signaling to confer sexually dimorphic synapse maturation and maintenance. Future studies are required to determine whether Vstm2b physically and functionally interacts with Hn. Furthermore, assessing synaptic density and function in gonadectomized *Vstm2b* WT and KO mice, as well as evaluating Nrxn2α expression and its SS2 splicing levels in these mice, may provide further insight into the mechanisms through which Vstm2b contributes to hippocampal sexual dimorphism.

While this study identifies synaptic defects in the vCA1 and associated behavioral abnormalities in *Vstm2b* KO mice, further work is necessary to directly test the causal relationship between these phenotypes. The ventral hippocampus, particularly the vCA1, has been strongly implicated in the regulation of social memory and anxiety-related behaviors. Previous studies have shown that vCA1 neuronal ensembles encode social memory and regulate social recognition through projections to downstream limbic regions such as the nucleus accumbens^47^, while integrating information from upstream inputs including the dCA2 and the amygdala^62^. Additionally, the vCA1 contains a subset of anxiety-responsive pyramidal neurons that are activated in anxiogenic environments and drive avoidance behavior^63,64^. Accordingly, optogenetic inhibition of vCA1 pyramidal neurons promotes exploration and produces anxiolytic effects, whereas activation of their projections to the lateral hypothalamic area (LHA) increases anxiety and avoidance^64^. The selective enrichment of Vstm2b in vCA1 pyramidal neurons relative to other hippocampal excitatory neurons suggests that loss of *Vstm2b* in this population may drive cell-autonomous synaptic dysfunction and compromised synaptic integrity, potentially underlying at least part of the observed behavioral abnormalities. At the same time, Vstm2b is also expressed in other social- and anxiety-related regions, including the amygdala and the hypothalamus, as well as in hippocampal GABAergic interneurons. Targeted viral delivery of Cre recombinase into the vCA1 of *Vstm2b*-floxed mice, or conditional deletion of Vstm2b in glutamatergic or GABAergic neurons, would help elucidate whether and how region- or cell-type–specific loss of Vstm2b drives local circuit dysfunction within the vCA1 and contributes to behavioral impairments.

Alternative splicing of α-Nrxns at SS2 is critical for α-Nrxn-Vstm2 interaction. SS2 splicing exhibits pronounced cell type- and gene-specific regulation, with predominant inclusion of SS2 inserts in Nrxn2α in hippocampal glutamatergic neurons. In addition, Vstm2b selectively induces glutamatergic, but not GABAergic, presynaptic assembly through axonal Nrxn2α. These findings support a model in which Nrxn2α^SS2+^ regulates vCA1 glutamatergic synapse function and maintenance through interactions with Vstm2b. While inclusion of SS2 inserts favors the binding of Nxph1 to α-Nrxns, it prevents dystroglycan (DAG) association with the LNS2 domain of α-Nrxns^21,65^. Unlike Vstm2b, both Nxph1 and αDAG are restricted to inhibitory synapses where they support synaptic plasticity and homeostasis^24,66^. Together, these observations indicate that cell-type-dependent splicing at the Nrxn2α SS2 site orchestrates a diverse repertoire of ligand interactions, thereby endowing Nrxn2α with context-specific roles in synaptic function and integrity. Building on studies demonstrating that SS4-dependent splicing of Nrxn1 and Nrxn3 shapes the Nrxn interactome and regulates NMDAR and AMPAR function^17–19^, analogous manipulation of SS2 splicing in Nrxn2α may offer valuable insight into its isoform-specific synaptic functions.

Vstm2a and Vstm2l were proposed to be secreted proteins^31,61^, but our results indicate that Vstm2a is membrane-bound and Vstm2l remains cytosolic. Our in silico analysis predicted a C-terminal GPI anchor of Vstm2a and Vstm2b, which we then validated by PI-PLC treatment. We also detected a soluble extracellular Vstm2a fragment, suggesting proteolytic release (ectodomain shedding). Strikingly, despite harboring a signal peptide, Vstm2l was not detected extracellularly, either as a secreted form or tethered to the cell surface. These findings are consistent with proteomic evidence identifying Vstm2a, but not Vstm2l or Vstm2b, in the neuronal mouse brain secretome^67^. Notably, the initial identification of Vstm2l as an Nrxn2α binding partner was obtained from AP-MS experiments performed on detergent-solubilized synaptosomes, in which intracellular proteins are accessible for affinity purification due to membrane disruption, likely explaining its isolation despite the absence of detectable extracellular localization. Whether Vstm2l can be secreted from specific cell types, either alone or together with a chaperone protein partner, remains unresolved. Notably, Vstm2l has been reported to localize to mitochondria in prostate cancer cells, interacting with VDAC1 to support mitochondrial homeostasis^68^. Vstm2b expression and localization also remain incompletely defined: we detected endogenous Vstm2b in synaptosomal and postsynaptic fractions, but antibody limitations prevented the precise determination of layer-specific expression or subcellular and subsynaptic localization in the vCA1 at the ultrastructural level. Multiple fixation strategies failed to immunolabel native Vstm2b in mouse tissue, suggesting that an epitope tagging approach may be required.

Building on previous evidence about Vstm2 members outside the nervous system, our findings provide a framework for investigating potential links between cell adhesion programs and signaling pathways in the CNS. In colorectal cancer cells, Vstm2a interacts with low-density lipoprotein receptor-related protein ^6^ (LRP6) and pinpoint programmed death ligand 1 (PD-L1)^32,34^. LRP6-mediated Wnt signaling is involved in synapse development and maintenance and has been implicated in ASD^69^, while the PD-L1/PD-1 immune checkpoint regulates neuronal excitability, synaptic plasticity, and learning and memory^70^. Future studies should characterize the synaptic roles of Vstm2 family members, including Vstm2a and Vstm2l, and determine whether they link α-Nrxns to these pathways in the regulation of CNS synapses. Furthermore, Vstm2a enhances bone morphogenetic protein (BMP) signaling in preadipocytes^31^, and Vstm2l promotes interleukin-4 (IL-4) signaling in colorectal cancer^33^. BMP signaling controls excitation-inhibition balance^71^, while neuronal IL-4 signaling modulates network activity and cognition^72^. Together, these findings suggest that Vstm2 family members may regulate synaptic function in the CNS by integrating multiple signaling pathways with α-Nrxn–based adhesion programs as multifaceted modulators.

## Methods

### Animal and ethics statement

All animal procedures were conducted in accordance with the guidelines of the Canadian Council on Animal Care and were approved by the IRCM Animal Care Committee (Protocol number: 2020-10 and 2024-06). Mice were housed in groups of two to five per cage under a 12-h light/dark cycle at 22–24 °C, 30–50% humidity, and with ad libitum access to food and water. All ex vivo and in vivo experiments and analyses included both male and female mice, and data were analyzed separately by sex. Sex was considered in the study design. For all experiments and analyses, investigators were blinded to genotype.

### Generation of *Vstm2b* LacZ reporter mouse line and *Vstm2b* global knockout mouse line

To generate *Vstm2b* mutant mice, ES cell clones (*Vstm2b*^tm1a(EUCOMM)Hmgu^) were obtained from the European Conditional Mouse Mutagenesis Consortium (Eucomm)^41^. This cell line (#HEPD0724_2_F02) was generated using a targeting vector (#PG00191_Z_4_A05) containing the Engrailed-2 splice acceptor upstream of a lacZ reporter, and *Vstm2b* exons 3, 4, and 5 are floxed. Homologous recombination of the targeting vector in the ES cells was verified by long-range PCR. On-target homologous recombination was verified in *Vstm2b*^tm1a^ mouse genomic DNA by Southern blotting. ES cells were injected into C57BL/6 J blastocysts to generate chimeras in the same B6 genetic background and underwent germline transmission by crossing with C57BL/6J mice to produce the *Vstm2b*^tm1a^ line. The *Vstm2b*^tm1c^ line was generated by crossing *Vstm2b*^tm1a^ mice with ACTB:FLPe B6J mice that induces germline deletion of FRT-flanked sites (The Jackson Laboratory, JAX stock #005703, B6.Cg-Tg(ACTFLPe)9205Dym/J). The *Vstm2b*^tm1d^ line was generated by crossing *Vstm2b*^tm1c^ mice with E2a-Cre B6J mice that achieve germline deletion of loxP-flanked sites (The Jackson Laboratory, JAX stock #003724, *B6*.FVB-Tg(EIIa-cre)C5379Lmgd/J). *Vstm2b*^tm1d^ mice were backcrossed at least 7 times and maintained on a C57BL/6J background. Removal of the FLPe and Cre transgenes was verified by PCR. Genotyping of *Vstm2b* mutant mice was performed using the following primers: *Vstm2b*^tm1a^: F1(5′-GGG GAC CCT TTG CTA AGG CCA CGG-3′), R1 (5′-CCG CCA CTT GCT GCA GGC TAC TCC-3′) and R2 (5′-CAC AAC GGG TTC TTC TGT TAG TCC CAA CCC CTT CCT C-3′); *Vstm2b*^tm1c^: F1(5′-GGG GAC CCT TTG CTA AGG CCA CGG-3′) and R1 (5′-CCG CCA CTT GCT GCA GGC TAC TCC-3′); *Vstm2b*^tm1d^: F1(5′-GGG GAC CCT TTG CTA AGG CCA CGG-3′); R1 (5′-CCG CCA CTT GCT GCA GGC TAC TCC-3′) and R3 (5′-CCA AAC TTG GGG ATC TGA GAG TAC AGT TCT CAA TGC TGT CCG-3′). The expected sizes of the PCR products are: tm1a +/+ = 312 bp, −/− = 438 bp; tm1c +/+ = 312 bp, fl/fl = 499 bp; tm1d +/+ = 312 bp’ −/− = 584 bp. In this study, we used homozygous *Vstm2b*^tm1d^ mice as Vstm2b global knockout mice (*Vstm2b* KO) and wild-type (WT) littermates as control mice. *Nrxn2α* global knockout (*Nrxn2α* KO) mice were genotyped as previously described^16^.

### Plasmids

Full length cDNAs encoding mouse Vstm2a (#MR202852), Vstm2b (#MR223744) and Vstm2l (#MR202504) were purchased from Origene. Mouse Vstm2a, Vstm2b, and Vstm2l lacking the signal peptide were cloned in frame with the TrkC signal peptide with an N-terminal Myc, HA, or HA-Myc epitope tag. Vstm2b-Fc was generated by PCR-amplifying mouse Vstm2b lacking the N-terminal signal peptide and the C-terminal GPI signal sequence (aa 29–263) and subcloning the PCR fragment between and in frame with the Nrxn1β signal peptide and human Fc in the pcDNA4 vector. Extracellularly tagged Myc-Nrxns were generated by PCR-amplifying Nrxns lacking the signal peptide using a series of HA-Nrxn constructs as templates and subcloning the PCR fragments in frame with the TrkC signal peptide and an N-terminal Myc epitope tag. Myc-Nrxn mutants were generated by site-directed mutagenesis^15^. HA-Nlgn1, HA-CD4, Nlgn1-Fc, and Fc were previously described^16,73^. All DNA constructs were verified by sequencing.

### Primary rodent neuron cultures and plasmid transfection

Cultures and transfection of rat or mouse hippocampal neurons were performed essentially as described previously^15,16^. Briefly, hippocampal or cortical neurons from E18 rat embryos were cultured on poly-L-lysine-coated glass coverslips in Neurobasal medium (Gibco, Cat#:21103-049) supplemented with NeuroCult SM1 (StemCell, Cat#: 05711) and GlutaMaX (Gibco, Cat#: 35050061). Transfection of HA-Vstm2b into rat hippocampal neurons was performed at 8 days in vitro (DIV) using a ProFection Mammalian Transfection System kit (Promega; #E1200) kit according to the manufacturer’s instructions. For mouse neuron cultures, hippocampi or cortices from E16-18 embryo littermates derived by crossing *Nrxn2α*- or *Vstm2b-*heterozygous mice were extracted and stored individually in tubes containing Hibernate E (BrainBits, Cat#: HE500) for around 4 h until genotyping results were obtained. Hippocampi or cortices from littermates of the same genotype were pooled, dissociated using trypsin (0.25%) for 15 min at 37 °C or papain (20 units/ml) for 30 min at 37 °C, respectively, and the resulting cells were plated onto poly-L-lysine-coated coverslips at ~80,000 cells/cm^2^. For *Vstm2b* KO and WT mouse neuron cultures, 1 × 10^6^ WT or KO cells were transfected with pCAG-EGFP plasmid (Addgene, plasmid #11150) using the Amaxa Nucleofector system (Lonza; program O-003) prior to plating onto coverslips. Transfected and non-transfected WT or KO cells were then mixed at a 1:9 ratio before plating. For mouse neuron cultures, insulin (5 μg/ml, Invitrogen, Cat#: I5506) was added to the supplemented Neurobasal medium. For low-density cultures, the neurons were cultured on glass coverslips inverted over a feeder layer of rat astrocytes.

### Production of soluble fusion proteins

The following recombinant proteins were purchased from R&D Systems: C-terminally 6xhistidine (His)-tagged mouse Vstm2a (Vstm2a-His (Cat#: 2283-VT-050)) and human Vstm2b (Vstm2b-His, Cat#: 10061-VT-050), C-terminally Fc-tagged mouse Vstm2b (Vstm2b-Fc, Cat#: 10076-VT-050) and human Nrxn2α (Nrxn2α-Fc, Cat#: 6636-NX-025), N-terminally HA-tagged human and mouse Vstm2l (HA-Vstm2l, Cat#: 2111-VT-050 and 2152-VT-050, respectively). C-terminally Fc-tagged mouse Nrxn2α ECD (Nrxn2α-Fc), used for unbiased proteomic pull-down assays, was produced as previously described^15^. His-tagged human Neurexophilin 1 (Nxph1-His; F119–G271; UniProt ID: BC047505), the extracellular fragment of human Vstm2b with the Ig V-set domain (A^26^-R^151^; UniProt ID A6NLU5), the extracellular fragment of human Nrxn2α with domains L1-egfA-L2 (D^27^-D^488^; UniProt ID: Q9P2S2) together with an N-terminal linker TGV without splice inserts (SS1−; SS2−) or carrying splice inserts SS1 (E^244^EHPMEGPAHLTLNSEVGSLLFSEGGAGRGGAGDVHQPTK^283^) and SS2AB (H^385^AGIGHAMVNKLHYL^399^) (SS1+; SS2AB+), each followed by a C-terminal ASTSHHHHHH tag, were produced using baculovirus-mediated overexpression in HighFive insect cells (cell line: BTI-Tn-5B1-4, RRID: CVCL_C190), and purified on a Ni-NTA column. Molecular weight and purity of the protein were evaluated by SDS–PAGE and confirmed by mass spectrometry and N-terminal sequencing.

### Surface plasmon resonance

Nrxn2α LNS1–egfA–LNS2 (with or without splice inserts) was immobilized as ligands on a CM5 Series S sensor chip (Cytiva) via EDC/NHS amine coupling with ethanolamine quenching for use on a Biacore T100 instrument. Pre-concentration of the Nrxn2α splice variants on the chip surface was performed at pH 4.6–4.7. A concentration series of Vstm2b (analyte) ranging from 0.4 to 19.2 µM in running buffer (25 mM HEPES, 100 mM NaCl, pH 8.0) was injected over the sensor surface at a flow rate of 27 µL/min, with an association phase of 80 s followed by a dissociation phase of 600 s. The sensor surface was regenerated after each injection using regeneration buffer (25 mM HEPES, 500 mM NaCl, pH 8.0) applied for 30 s at 30 µL/min, followed by 60 s stabilization in running buffer. Each sensorgram was recorded in triplicate, and binding responses were analyzed using steady-state fitting.

### Heterologous synapse formation assays

Cocultures of hippocampal or cortical rat or mouse neurons with HEK293T cells (RRID: RRID:CVCL_0063) or Protein G Dynabeads (ThermoFischer Scientific, Cat#: 10004D) coated with recombinant Fc-tagged proteins were prepared as previously described^15^. For neuron-HEK293T co-culture assays, HEK293T cells were transfected with the desired plasmids using TransIT-LT1 (Mirus Bio. LLC, Cat#: MIR2305) and cultured in DMEM containing 10% fetal bovine serum (FBS). 24 h after transfection, cells were harvested by trypsinization and seeded onto the neuron cultures. For bead-based artificial synapse formation assays, HEK293T cells were transfected with plasmids encoding mouse Vstm2b-Fc, Nlgn1 A^−^B^−^-Fc, or Fc using TransIT-LT1 (Mirus Bio. LLC, Cat# MIR2305) to prepare conditioned medium. The conditioned medium was spiked with Protein G Dynabeads to bind the Fc proteins, and then the beads were washed with phosphate-buffered saline (PBS) before being applied onto neurons. After 24 h, cultures were fixed with prewarmed parafix solution (4% paraformaldehyde and 4% sucrose in PBS, pH 7.4) for 12 min and blocked with 5% normal donkey serum (NDS) and 3% BSA in PBS for 1 h at room temperature. Cells were incubated with anti-HA (1:1000, polyclonal rat, Roche, Cat#: ROAHAHA) overnight at 4 °C to label transfected surface-expressed HA-tagged synaptic organizers. Cells were then permeabilized in 0.2% Triton X-100 in PBS and incubated with anti-VGAT (1:500, polyclonal rabbit, Synaptic Systems, Cat#: 131003) or anti-VGLUT1 (1:500, polyclonal guinea pig, Synaptic Systems, Cat#: 135 304) overnight at 4 °C. Cells were then incubated with highly cross-adsorbed Alexa dye-conjugated secondary antibodies generated in donkey toward the appropriate species (1:500, Jackson ImmunoResearch). Samples were imaged using a Leica DM6 widefield microscope (Leica Microsystems) with a 40X oil objective.

### In situ surface binding assays using soluble proteins

Protein binding assays were performed as described previously^15,16^. Briefly, to assess protein binding on the surface of COS-7 cells (RRID: CVCL_0224), the cells were transfected using TransIT-LT1 (Mirus Bio. LLC, Cat#: MIR2305) with the appropriate plasmids and then cultured in DMEM containing 10% (v/v) FBS. 24 h after transfection, the transfected COS-7 cells were washed with extracellular solution (ECS) containing 168 mM NaCl, 2.4 mM KCl, 20 mM HEPES, pH 7.4, 10 mM D-glucose, 2 mM CaCl_2_, 1.3 mM MgCl_2_, and 100 μg/ml bovine serum albumin (BSA; Sigma, Cat#: A9647). The cells were then incubated for 1 h at 4 °C with the appropriate soluble proteins (Vstm2a-His, Vstm2b-Fc or HA-Vstm2l) diluted to 500 nM in ECS/BSA. Cells were washed with ECS and subsequently fixed in prewarmed parafix solution for 12 min and blocked in 5% (v/v) NDS and 3% (w/v) BSA in PBS for 1 h at room temperature. Primary antibodies were the following: anti-HA (1:1000, polyclonal rat, Roche, Cat#: ROAHAHA), anti-HA (1:2000, polyclonal rabbit, Abcam, Cat#: ab9110), anti-myc (1:1000, monoclonal mouse, clone 9E10, Santa Cruz, Cat#: sc-40), anti-6xHis (1:1000, polyclonal rabbit, Abcam, Cat#: ab213204). Cells were then incubated with highly cross-adsorbed Alexa dye-conjugated secondary antibodies generated in donkey toward the appropriate species (1:500, Jackson ImmunoResearch). Samples were imaged using a Leica DM6 widefield microscope (Leica Microsystems) with a 40X oil objective.

### Immunoblotting

For all samples, protein concentrations were measured in DC protein assays (Bio-Rad, Cat#: 500-0112). After normalizing their protein concentration, samples were run on 10 or 12% polyacrylamide gels. Gels were transferred onto PVDF membranes (Millipore Sigma, Cat#: GE10600023). Membranes were blocked in 3% BSA and 0.1% Tween 20 in Tris-buffered saline (TBS) and incubated with one of the following primary antibodies: 6xHis (1:2000, polyclonal rabbit, Abcam, Cat: ab9108), HA (1:3000, polyclonal rabbit, Abcam, Cat#: ab9110), β-actin (1:5000, polyclonal rabbit, Abcam, Cat#: ab8227), Myc (1:1000, monoclonal mouse IgG1, clone 9E10, Santa Cruz Biotechnology, Cat#: sc-40), Vstm2b (1:3000, polyclonal guinea pig, custom made, kindly provided by Dr. Amy L. Norovich; validated using *Vstm2b* KO brain samples, Supplementary Fig. 7C), Vstm2a (1:400, polyclonal rabbit, Origene, Cat#: AP54523PU-N), GAPDH (1:1000, mouse IgG1, clone 0411, Santa Cruz Biotechnology, Cat: sc-47724), PSD95 (1:2000, mouse IgG2a, clone 6G6-1C9, ThermoFischer Scientific, Cat#: MA1-045), GPHN (1:1000, mouse IgG1, clone 3B11, Synaptic Systems, Cat#: 147 111), SYP (1:10,000, mouse IgG1, clone SY38, Millipore, Cat#: MAB5258). Membranes were washed with TBS containing 0.1% Tween 20 and incubated with the appropriate horseradish peroxidase (HRP)-conjugated secondary antibodies (1:10,000, Jackson ImmunoResearch). Signals were developed using Immobilon Western Chemiluminescent HRP Substrate (Millipore, Cat#: WBKLS0500) and captured by ImageQuant LAS 4000 (GE Healthcare).

### Cell surface biotinylation of rat cortical neuron cultures

Surface biotinylation assays were performed according to the kit’s instructions (Thermo Scientific, Cat#: A44390), essentially as previously described^16^. Briefly, at 14 days in vitro (DIV), rat cortical neurons were washed with PBS and then incubated with 0.25 mg/ml EZ-Link Sulfo-NHS-SS-Biotin in PBS at room temperature for 10 min. The biotinylation reaction was quenched by washing three times with ice-cold TBS. Following lysis in RIPA buffer supplemented with a cocktail of protease inhibitors (Roche; Cat#: CO-RO), the biotinylated proteins were purified using NeutrAvidin-conjugated agarose beads, resolved by SDS-PAGE, and immunoblotted with the corresponding antibodies or submitted for mass spectrometry analysis.

### Glycosylation analysis

For glycosylation profiling, HEK293T cells were transfected with plasmids encoding Myc-tagged Vstm2 using TransIT-LT1 (Mirus Bio. LLC; Cat#: MIR2305) and then cultured in DMEM containing 10% (v/v) FBS. 24 h after transfection, cells were lysed in RIPA buffer, and protein extracts were treated with PNGaseF (New England Biolabs (NEB), Cat#: P0704S) or O-glycosidase (NEB, Cat#: P0733S) and/or neuraminidase (NEB, Cat#: P0720S) according to the manufacturer’s instructions. Alternatively, cells were treated with 2 μg/ul of Tunicamycin (Sigma, Cat#: T7765), cultured for another 24 h, lysed in RIPA buffer, and immunoblotted. For assessing glycosylphosphatidylinositol (GPI) modification of Vstm2a and Vstm2b, HEK293T cells were transfected with HA-myc-Vstm2a or HA-myc-Vstm2b. 24 h after transfection, cells were washed twice with PBS, incubated with 1 U/ml of phosphatidylinositol-specific phospholipase C (PI-PLC, Invitrogen, Cat#: P6466) for 1 h at 37 °C. Cells were lysed in RIPA buffer containing protease inhibitors. Supernatants were incubated with 2.5 μg/ml anti-HA (polyclonal rabbit, Abcam, Cat#: ab9110) for 2 h at 4 °C with rotation. Protein G Dynabeads (50 μl of beads slurry per ml of supernatant) were added, and the mixtures were incubated for another hour at 4 °C. Beads were washed three times with ice-cold PBS and eluted with 2x Laemmli buffer containing β-mercaptoethanol. Samples were resolved by SDS-PAGE and immunoblotted with anti-Myc antibody (1:1000, monoclonal mouse, clone 9E10, Santa Cruz, Cat#: sc-40).

### Subcellular fractionation

Synaptic fractionation was carried out essentially as previously described^74^. All steps were performed on ice or at 4 °C. Briefly, forebrains from 1-month-old mice were homogenized using a Douncer in homogenization buffer [0.32 M sucrose, 4 mM HEPES pH 7.4, 1 mM MgCl_2_, supplemented with a cocktail of protease inhibitors (Roche, Cat: #CO-RO) and phosphatase inhibitors (Roche, Cat#: PHOSS-RO), homogenate fraction]. The homogenate was centrifuged at 1500 × *g* for 15 min to collect the supernatant (post-nuclear fraction). The supernatant was further centrifuged at 18,000 × *g* for 20 min and the resulting supernatant (cytosol fraction) and pellet (crude synaptosome fraction) were collected. The pellet was resuspended in homogenization buffer and loaded on a discontinuous sucrose gradient (0.85 M/1.0 M/1.2 M) and centrifuged at 78,000 × *g* for 2 h. The material at the 1.0 M/1.2 M interface was carefully collected (synaptosome fraction). Triton X-100 was added to 0.5% (v/v), and the synaptosome samples were incubated at 4 °C on a rotating platform for 20 min before being centrifuged at 32,000 × *g* for 2 h. The supernatant was collected (Triton-soluble synaptosome fraction), and the pellet was resuspended in homogenization buffer, loaded on a discontinuous sucrose gradient (1.0 M/1.5 M/2.0 M), and centrifuged at 170,000 × *g* for 2 h. The material at the 1.5 M/2.0 M interface was carefully collected. Triton X-100 was added to 0.5% (v/v) and incubated at 4 °C on a rotating platform for 10 min before being centrifuged at 100,000 × *g* for 20 min. The resulting pellet was resuspended in homogenization buffer (Triton-insoluble synaptosome fraction). Proteins were resolved by SDS-PAGE and immunoblotted with appropriate antibodies.

### Pull-down assays

For pull-down assays with highly purified proteins, 3 μg of human Nrxn2α-Fc (R&D Systems, Cat#: 6636-NX-025) or Fc alone were incubated with 3 μg of human Vstm2a-His (R&D Systems, Cat#: 10037-VT-050), Vstm2b-His (R&D Systems, Cat#: 10061-VT050) or HA-Vstm2l (R&D Systems, Cat#: 2111-VT-050) in 500 μl of binding buffer (10 mM HEPES pH 7.4, 150 mM NaCl, 2 mM CaCl_2_, 1 mM MgCl_2_ and 0.1% Tween-20) for 1 h at room temperature. Then, 50 μl of Protein G magnetic bead slurry (Dynabeads, ThermoFischer Scientific, Cat#: 10004D) was added, and the samples were incubated for 1 h at 4 °C on a rotating platform. Protein complexes pulled down by the Protein-G beads were purified using magnetic separation (DynaMag-2 Magnet, ThermoFischer Scientific, Cat#: 12321D) to wash the Protein G beads once with binding buffer followed by four washes with PBS. Samples containing protein complexes attached to the beads were boiled in 2x Laemmli buffer containing β-mercaptoethanol, resolved by SDS-PAGE, and immunoblotted with the corresponding antibodies.

For mouse brain pull-down assays coupled to mass spectrometry analysis, crude synaptosome fractions were prepared as previously described^75^. Briefly, brains from 1-month-old mice were homogenized using a Douncer with homogenization buffer [0.32 M sucrose, 4 mM HEPES pH 7.4, 1 mM MgCl_2_, supplemented with a cocktail of protease inhibitors (Roche, Cat#: CO-RO)]. The homogenate was centrifuged at 1500 × *g* for 15 min, and the supernatant was further centrifuged at 18,000 × *g* for 20 min. The pellet (crude synaptosome fraction) was resuspended in homogenization buffer containing 1.5% CHAPS detergent and incubated at 4 °C for 2 h on a rotating platform before being centrifuged at 20,000 × *g* for 30 min. Protein-G magnetic beads (Dynabeads; Invitrogen; Cat#: 10004D) were coated with Nrxn2α-Fc or control Fc protein. Coated beads were added to the extracted crude synaptosome fractions, and the samples were incubated at 4 °C overnight on a rotating platform. Magnetic separation (DynaMag-2 Magnet; Invitrogen, Cat#:12321D) was used to wash the beads three times with homogenization buffer containing 1.5% CHAPS detergent and five times with 50 mM ammonium bicarbonate (AmBic).

For rat primary hippocampal neuron culture pull-down assays coupled to mass spectrometry analysis, protein-G magnetic beads coated with Vstm2b-Fc or Fc alone were added to the cultured neurons at 14 DIV. After 24 h, cultures were washed with PBS and crosslinked with 2 mM 3,3ʹ-dithiobis (sulfosuccinimidyl propionate) (DTSSP; Pierce, Cat#: 21578) in PBS for 30 min at room temperature. Neurons were then sonicated in lysis buffer [10 mM Tris-HCl (pH 8.0), 10 mM KCl, 5 mM EDTA, 5 mM EGTA, 0.6% Nonidet P-40, and protease inhibitors (cOmplete ULTRA Tablets, EDTA-free, Roche, Cat#: 5892953001)] and incubated for 1 h at 4 °C on a rotating platform. Bound proteins were purified by magnetic separation and washed three times in lysis buffer. The beads were further washed five times with 50 mM Tris-HCl (pH 7.5)/2% SDS, five times with 50 mM Tris-HCl (pH 7.5), and five times with 50 mM ammonium bicarbonate (AmBic).

### Prediction of the Nrxn2α/Vstm2b complex

A modified form of AlphaFold2 was utilized by local installation of ColabFold (v. 1.5.5) to model the complex formed by Nrxn2α-LNS2 (UniProt ID: E9Q7X7, res. 288 to 490) and Vstm2b-v-Ig (UniProt ID: Q9JME9, res. 29 to 153) using MMseqs2 on a computer with the following specifications/components: Intel(R) Xeon(R) CPU E5-2670 CPU @ 2.60 GHz, 8 cores, 16 threads, 64 GB DDR3 RAM, NVIDIA Quadro M6000 24GB. Nrxn2α-LNS2 was modeled from the following PDB templates (assorted by PDB ID and the polypeptide chain): 3poy_A, 3qcw_B, 3r05_B, 6pnq_A, 6pnp_A, 6cw1_B, 2h0b_A, 3sh5_A, 3sh4_A, 8s9p_A, 2jd4_B, 2jd4_A, 3r05_B, 3asi_A, 1pz8_A, 1pz8_B, 1pz7_B, 3qcw_B, 1pz9_B. Vstm2b was modeled from the following PDB templates (assorted by PDB ID and the polypeptide chain): 1pkq_J, 3csp_A, 1pko_A, 1py9_A, 4f8t_A, 4f9p_B, 4f80_A, 2n4i_A, 4f9l_B, 4f9p_A, 6xlq_A, 4f8q_A, 4k55_A, 4hh8_A, 6l7z_A, 4i0k_A, 6x4t_C, 4gos_A, 6np9_A, 6nnv_D. Five relaxed models were generated (4 recycles until convergence). The complex model with the best pLDDT (predicted local-distance difference test) score was chosen. The predicted complex-model, as well as the experimental structure of the Nrxn1α-LNS2^SS2A+^/Nxph1 complex, were visualized with PyMOL (v. 3.0.4).

### Mass spectrometry data analysis

The on-bead pulled-down proteins were first incubated in 4 M urea with 50 mM AmBic for 10 min and then diluted in 2 M urea with 50 mM AmBic for a tryptic digestion performed overnight at 37 °C with agitation using 0.3 μg of sequencing grade modified trypsin (Promega, Cat#: V5111). The samples were then reduced with 13 mM dithiothreitol at 37 °C for 30 min and, after cooling for 10 min, alkylated with 23 mM chloroacetamide at room temperature for 20 min in the dark. The supernatants were then acidified with trifluoroacetic acid for desalting and removal of residual detergents using an Oasis MCX 96-well Elution Plate (Waters, Cat#: 186001830BA) following the manufacturer’s instructions. After elution in 90% methanol with 10% ammonium hydroxide (v/v), samples were dried with a Speed-Vac, reconstituted under agitation for 15 min in 12 μL of 2% acetonitrile and 1% formic acid, and loaded onto a 75 μm i.d. × 150 mm Self-Pack C18 column installed in an Easy-nLC II system (Proxeon Biosystems). Peptides were eluted with a two-slope gradient at a flow rate of 280 nl/min. The buffers used for chromatography were 0.2% formic acid in water (solvent A) and 0.2% formic acid in acetonitrile (solvent B). Solvent B was first increased from 2 to 37% over 90 min and then from 37 to 80% over 10 min. The HPLC system was coupled to an Orbitrap Fusion mass spectrometer (ThermoFisher Scientific) through a Nanospray Flex Ion Source. Nanospray and S-lens voltages were set to 1.3–1.7 kV and 60 V, respectively. The capillary temperature was set to 250 °C. Full scan MS survey spectra (m/z 360–1560) in profile mode were acquired in the Orbitrap with a resolution of 120,000 with a target value of 3 × 10^5^. The 25 most intense peptide ions were fragmented in an HCD collision cell and analyzed in the linear ion trap with a target value of 2 × 10^4^ and a normalized collision energy at 29 V. Target ions selected for fragmentation were dynamically excluded for 15 s after two MS2 events.

Liquid chromatography-tandem mass spectrometry (LC-MS/MS) raw files were analyzed with the Mascot search engine using the *Mus musculus* RefSeq database (version 20151112) supplemented with “common contaminants” from the Max Planck Institute, the global proteome machine (GPM), and decoy sequences. The search parameters were set with trypsin specificity (two missed cleavage sites allowed), and variable modifications involving oxidation (M), deamidation (NQ), and carbamidomethyl (C) were set as fixed modifications. The mass tolerances for precursor and fragment ions were set to 10 ppm and 0.6 Da, respectively, and peptide charges of +2,+3, and +4 were considered. Search results were individually processed by PeptideProphet, and peptides were assembled into proteins using parsimony rules as previously described by using the trans-proteomic pipeline (TPP)^76,77^. TPP settings were the following: -p 0.05 -x20 -PPM –d “DECOY,” iProphet option pPRIME and PeptideProphet pP. Only proteins having at least one unique peptide and an iProphet probability ≥0.9 were considered.

To estimate the interaction statistics, proteins having iProphet protein probability ≥0.9 and unique peptides ≥1 were submitted to SAINTexpress (version 3.6.1 without control or bait compression)^78^. Potential hit proteins identified by the pulldown using Nrxn2α-Fc as bait were compared against pulldowns using the Fc control, and interactions displaying a Bayesian false discovery rate (BFDR) ≤ 0.03 were considered as statistically significant.

### Quantitative real-time PCR

Quantitative real-time PCR (qRT-PCR) reactions were performed in triplicate for each condition from RNA extracted from cerebellar, cortical, or hippocampal mouse tissues at indicated times. RNA was extracted using the RNeasy mini kit (Qiagen, Cat#: 74104) according to the manufacturer’s instructions, and genomic DNA was digested on column using RNase-free DNase I (Qiagen, Cat#: 79254). Isolated RNAs (2 μg) were subjected to reverse transcriptase using a high-capacity cDNA reverse transcription kit according to the manufacturer’s instructions (Applied Biosystems, Cat#: 4368814). Single strand cDNAs (6 ng) were processed for qRT-PCR using PowerUp SYBR Green Master Mix (ThermoFischer Scientific, Cat#: A25742) on a QuantStudio 5 RT-PCR system (Applied Biosystems, Cat#: A28140). Absolute quantification by qRT-PCR was performed using serial dilutions of recombinant DNA plasmids encoding mouse Vstm2a, Vstm2b, and Vstm2l. All probes for were used at 1 μM (Vstm2a: GTGGAGCTCTTACCCGACAGA/CATTGCCTTGGACTTTCACTGTAC; Vstm2b: TCTGAAACCAGCCACGAT GAC/CAGAGGATCGGCACTGTAGC; Vstm2l: GGACACGCACTCTTCACAGA/ACGTCTGCTTGTCAGTCCAG; Actb: CCTGAACCCTAAGGCCAACC/ATGGCGTGAGGGAGAGCATA). To ensure specificity of the amplification, melt curve analyses were performed for all reactions. Transcript levels were normalized to the β-actin internal control.

### Analysis of RNA-seq data for quantification of α-Nrxn alternative splicing at the SS2

Raw fastq files (GEO:GSE133291) were downloaded from NCBI’s sequence read archive^44^. The reads were aligned to the Ensembl GRCm39 reference genome with STAR v2.7.11b, and raw counts were calculated with FeatureCounts v2.0.6 based on the GRCm39 reference genome (release 110). Reads mapped onto the SS2 exons of *Nrxn1*, *Nrxn2*, and *Nrxn3* were quantified using DEXseq and classified as SS2-positive (SS2+) or -negative (SS2−). Since the SS2 motif fell within an intron for *Nrxn3*, we created an exon for this specific gene. To estimate the number of SS2+ and SS2− reads for each of the 3 genes, sashimi plots were generated for the SS2 exons and their flanking constitutive exons using rmats2sashimiplot v.3.0.0. The number of reads bypassing SS2 was then classified as SS2− for *Nrxn1*, *Nrxn2*, and *Nrxn3*. Reads were normalized as counts per million (CPM), and the read counts from cell type replicates were averaged together. The fraction of SS2+ and SS2− were then calculated to define the proportions for each gene.

### Nissl staining

Four-week-old homozygous *Vstm2b* KO mice and WT littermates were transcardially perfused with PBS followed by 4% PFA in PBS (PFA/PBS). Fixed brains were dissected, postfixed overnight at 4 °C with 4% PFA/PBS, and washed with PBS the following day. Neural tissues were then incubated in 30% sucrose solution at 4 °C with shaking for 2–3 days for cryoprotection, embedded in O.C.T. compound (ThermoFischer Scientific, Cat#: 23-730-571), and finally stored at −80 °C until sectioning. 40-μm-thick frozen coronal sections were cut and mounted on Superfrost Plus slides (VWR) and then incubated overnight in 1:1 alcohol/chloroform solution. The slides were then rehydrated in sequential baths containing 100%, 95%, 70% ethanol and finally distilled water. The sections were stained by immersion into warm 0.1% cresyl violet solution for 10 min, rinsed in distilled water, and differentiated in 95% ethanol. Slides were then dehydrated in 100% ethanol, cleared in xylene, and mounted with Eukitt® Quick-hardening mounting medium (Sigma-Aldrich, Cat#: 03989).

### X-gal staining

Four-week-old heterozygous *Vstm2b* LacZ mice (*Vstm2b*^tm1a^ line) were transcardially perfused with PBS followed by 4% PFA/PBS. Fixed brains were dissected, postfixed 2 h at 4 °C with 4% PFA/PBS, and then embedded in 3% agarose. 100-μm-thick brain sections were cut in the coronal or sagittal plane using a vibratome before fixation with 0.2% glutaraldehyde/PBS for 5 min at room temperature. Following three washes with PBS, sections were incubated in 5-bromo-4-chloro-3-indolyl β-D-galactopyranoside (X-gal) working solution (1 mM MgCl_2_, 3 mM K_4_[Fe(CN)_6_]•3H_2_O, 3 mM K_3_[Fe(CN)_6_], 0.1% Triton X-100, 1 mM X-gal) at 37 °C for 24 h in the dark. Sections were washed with 1 mM EDTA/PBS solution to stop the reaction and mounted using a mounting medium (5% DABCO (wt/vol) and 90% glycerol in 0.02 M Na_2_HPO_4_). Images were acquired in tile scanning mode using a Leica DM6 widefield microscope with a 10× objective and a DFC480 camera.

### Immunohistochemistry

For synapse detection, *Vstm2b* KO mice and sex-matched WT littermates were transcardially perfused with PBS followed by either glyoxal fixative for glutamatergic synapses (9% glyoxal/8% acetic acid dissolved in double-distilled water, pH 4.0) or 4% PFA/PBS for GABAergic synapses. Brains were dissected, postfixed overnight at 4 °C with glyoxal (glutamatergic synapses) or 4% PFA/PBS (GABAergic synapses), and washed with PBS the following day. Fixed brains were then incubated in 30% sucrose solution at 4 °C with shaking for 2–3 days for cryoprotection, embedded in O.C.T. compound, and finally stored at −80 °C until sectioning. Frozen brains were cut on a cryostat, and free-floating 40-μm coronal sections were blocked for 1 h at room temperature in PBS containing 5% NDS, 3% BSA, and 0.1% Triton X-100 and incubated with primary antibodies overnight at 4 °C. Primary antibodies were the following: anti-VGLUT1 (1:1000, polyclonal rabbit, Synaptic Systems, Cat#: 135 308), anti-PSD95 (1:1000, monoclonal mouse, clone 6G6-1C9, ThermoFisher Scientific, Cat#: MA1-045), anti-VGAT (1:500, polyclonal rabbit, Synaptic Systems, Cat#: 131 003) and anti-GPHN (1:500, monoclonal mouse, clone mAb7a, Synaptic Systems, Cat#: 147 011). Sections were subsequently washed three times with PBS containing 0.1% Triton X-100 and incubated for 2 h with highly cross-adsorbed Alexa dye-conjugated secondary antibodies generated in donkey toward the appropriate species (1:500; Jackson ImmunoResearch) and DAPI for nuclear staining at room temperature. Sections were then washed three times with PBS containing 0.1% Triton X-100 and mounted on Superfrost Plus slides (VWR). Sections were imaged using a Zeiss LSM980 confocal microscope with a 63X oil-immersion objective. Single-plane 8-bit images (763 × 763 pixels) were captured with a 2.5× digital zoom, providing 0.073 × 0.073 µm/pixel resolution.

For fluorescent β-galactosidase (β-gal) detection, 4-week-old *Vstm2b*^tm1a^ heterozygous mice were transcardially perfused with PBS followed by 4% PFA/PBS. Fixed brains were dissected, postfixed 1 h at 4 °C with 4% PFA/PBS, and then embedded in 3% agarose. 70-μm-thick coronal sections were cut on a vibratome then blocked for 1 h at room temperature in PBS containing 10% NDS and 0.5% Triton X-100 and incubated with primary antibodies overnight at 4 °C. Primary antibodies were the following: anti-β-gal (1:500, monoclonal mouse; Promega, Cat#: Z3781), anti- parvalbumin (PVALB) (1:1000, polyclonal guinea pig, Swant, Cat#: GP72) and anti- calretinin (CR) (1:1000, polyclonal rabbit, Millipore, Cat#: ABN2191). Sections were subsequently washed three times with PBS containing 0.1% Triton X-100 and incubated for 2 h with highly cross-adsorbed Alexa dye-conjugated secondary antibodies generated in donkey toward the appropriate species (1:500, Jackson ImmunoResearch) and DAPI at room temperature. Sections were then washed three times with PBS containing 0.1% Triton X-100 and mounted on Superfrost Plus slides (VWR). Sections were imaged using a Zeiss LSM980 confocal microscope with a 40X oil-immersion objective. Single-plane 8-bit images (3005 × 3005 pixels) were captured, providing 0.073 × 0.073 µm/pixel resolution.

### Fluorescence image analysis

For in situ binding assays, the average intensity of bound soluble proteins was normalized to the average surface intensity of the membrane-tethered receptor signal. COS-7 cells expressing similar levels of surface receptors were selected to quantify bound soluble proteins. Analyses were performed using Volocity 6.0. For heterologous synapse formation assays, HEK293T cells displaying similar surface HA levels (corresponding to HA-Vstm2b or HA-CD4) were imaged without considering the other fluorescence channels. To assess presynaptic assembly, VGLUT1 and VGAT signals were thresholded, and the total thresholded intensity within the regions occupied by HEK293T cells was measured. Analysis was performed using Metamorph 7.8 (Molecular Devices). For analysis of β-gal fluorescence, the number of β-gal puncta was divided by the number of cells (assessed by DAPI staining) within a region of interest. Analysis was performed using QuPath. For spine density analysis, head-positive and head-negative spines on GFP-positive primary dendritic segments were counted and normalized to the analyzed dendritic length using Metamorph 7.8. For analysis of glutamatergic and GABAergic synapse density, images were analyzed with the SynBot macro. The settings were as follows: Channels: 2-channel colocalization (RG); Preprocessing: noise reduction (Subtract background rolling ball radius: 50; Gaussian Blur Sigma: 0.570); Thresholding: manual; ROI type: whole image; VGLUT1/VGAT min pixel size: 7 pixels; PSD95/GPHN min pixel size: 7 pixels; Analysis type: pixel-overlap. Each image was separately thresholded to extract synaptic puncta. All imaging and analyses were performed while blind to experimental conditions. For in vitro cell-based experiments, *n* represents the number of analyzed cells collected from multiple independent experiments, with a balanced contribution of cells from each experiment. For synapse density quantification in the ventral hippocampus, *n* represents a single mouse; 4–6 images per mouse were acquired from at least three different brain slices and averaged to generate a single value per mouse.

### Electrophysiology

For fEPSP recording, *Vstm2b* KO mice and sex-matched WT littermates were deeply anesthetized and decapitated. The brains were rapidly removed and placed in ice-cold slicing buffer containing (in mM): 2.5 KCl, 0.2 CaCl_2_, 4 MgCl_2_, 1.25 NaH_2_PO_4_, 26 NaHCO_3_, 10 D-glucose, and 25.2 sucrose, bubbled with the pH equilibrated to 7.35 by 95% O_2_/5% CO_2_. Acute 350-µm-thick coronal slices containing ventral hippocampus were obtained using a VT1000S vibratome (Leica Biosystems) and then incubated in carbogenated (5% CO_2_/95% O_2_) artificial cerebral spinal fluid (ACSF) containing (in mM): 125 NaCl, 2.5 KCl, 2 CaCl_2_, 1 MgCl_2_, 1.25 NaH_2_PO_4_, 26 NaHCO_3_, and 25 d-glucose at pH 7.35. Slices recovered in the incubation chamber at 32 °C for 1 h, followed by incubation at room temperature before recordings. Subsequently, slices were transferred to a recording chamber and were continuously perfused with carbogenated ACSF for the duration of the experiments. Extracellular field excitatory postsynaptic potentials (fEPSPs) were evoked in the CA1 stratum radiatum by stimulating the Schaffer collaterals or in the CA1 SLM by stimulating SLM afferents with a concentric bipolar microelectrode (FHC Inc.) and recorded using a glass pipette filled with ACSF. fEPSPs were amplified (MultiClamp 700B amplifier, Molecular Devices) and digitized (Digidata-1550B interface, Molecular Devices) for analysis. Input/output (I/O) curves were obtained by plotting the fiber volley (FV) amplitudes against fEPSP slopes. The paired-pulse ratio was measured at varying interpulse intervals (25, 50, 100, 200, and 500 ms) and calculated as the slope of the second fEPSP divided by the slope of the first fEPSP. Extracellular recordings were acquired and digitized by Clampex 11.2, and analysis was done through Clampfit 11.2. All recordings and analyses were performed while blind to experimental conditions. For quantification, *n* represents the number of mice. For each mouse, recordings were obtained from 3–5 brain slices, with 3–5 trials per stimulation condition (3 trials for SR and 5 trials for SLM) averaged within each slice. Input–output and paired-pulse protocols were performed at the same recording sites, and measurements were averaged across slices to yield a single value per mouse.

For mEPSC recording, the right hemisphere was separated in ice-cold cutting solution composed of (in mM): 210 sucrose, 11 D-glucose, 26.2 NaHCO_3_, 2.5 KCl, 1 NaH_2_PO_4_, 0.5 CaCl_2_, and 5 MgSO_4_ with the pH equilibrated to 7.3–7.4 by 95% oxygen and 5% carbon dioxide and the osmolarity adjusted to 290–300 mOsm/L (all chemicals and drugs were purchased from Sigma-Aldrich). The cutting solution was bubbled with 95% oxygen and 5% carbon dioxide. Acute 400 μm slices containing ventral hippocampus were obtained using a VT1000S vibratome (Leica Biosystems, Germany) and then transferred to an incubation chamber containing a standard artificial cerebral spinal fluid (ACSF) comprised of (in mM): 124 NaCl, 10 D-glucose, 26 NaHCO_3_, 2.5 KCl, 1.25 NaH_2_PO_4_, 2 CaCl_2_ and 1 MgSO_4_ at pH of 7.3–7.4 and osmolarity of 290–300 mOsm/L. ACSF was bubbled continuously with carbogen (95%O2/5%CO2). Slices recovered in the chamber at 32 °C for 40 min prior to experiments. For recordings, slices were transferred to a submerged recording chamber (Scientifica Instruments) mounted on an upright microscope (Olympus U-AN-2, Japan), and continuously superfused with oxygenated ACSF at 2–3 ml/min. Whole-cell patch-clamp recordings were obtained from CA1 pyramidal neurons, visually identified using a ×40 water-immersion objective and a CCD camera (Prime BSI Express IR camera, Canada). Patch pipettes were pulled from borosilicate glass (1.5 mm outer diameter, 0.86 mm inner diameter; Sutter Instrument) using a P-1000 puller (Sutter Instrument) and had a resistance of 4–6 MΩ. For voltage-clamp recordings, pipettes were filled with an intracellular solution containing (in mM): 120 Cs-gluconate, 10 CsCl, 0.3 Na-GTP, 4 Mg-ATP, 0.5 EGTA, 10 phosphocreatine-Na, 10 HEPES, and 5 QX-314-Cl, adjusted to pH 7.3–7.4 and 290–300 mOsm/L. Signals were recorded using a Multiclamp 700B amplifier, digitized at 10 kHz via a Digidata 1550B interface, and acquired with pClamp 11 software (Molecular Devices). Series resistance and electrode capacitance were compensated, and recordings were excluded if series resistance varied by more than 25%. Junction potentials were not corrected. For mEPSC measurements, CA1 pyramidal neurons were voltage-clamped at −70 mV. Tetrodotoxin (TTX; 1 μM) and bicuculline (10 μM) were included in the ACSF to block action potentials and GABA_A_ receptor-mediated inhibitory currents, respectively. mEPSC frequency and amplitude were analyzed using MiniAnalysis software. All recordings and analyses were performed while blind to experimental conditions. For quantification, *n* represents the number of analyzed neurons.

### Behavior analyses

All behavioral experiments were carried out on age- and sex-matched *Vstm2b*^tm1d^ WT and KO mice (2–4 months old) during similar daytime periods in an isolated behavior room with the room lighting set as described in each test. To minimize potential litter effects, each behavioral test included mice derived from at least six independent litters. In each cohort, animals from both genotypes were tested in parallel, with balanced representation of WT and KO mice from each litter. Starting a week prior to behavioral testing, mice were handled by the experimenter over two to three 5-min habituation sessions. Before every experiment, mice were transferred and habituated to the test room for at least 30 min. For all experiments and analyses, the experimenters were blind to genotype. For quantification, *n* represents a single mouse, with each mouse tested once in each behavioral paradigm. Mouse behaviors were video-recorded and analyzed using EthoVision XT17.5 software (Noldus). Behavioral tests were performed in the following order: open field, Y-maze, elevated plus maze, light-dark box, three-chamber social interaction, and contextual fear conditioning.

### Open field test

The experimental setup included an open square field (LxWxH: 50 × 50 × 38 cm) under a 100-lux overhead light. Mice were placed in the center of the arena and allowed to freely explore for 30 min. Time spent in the center (25% of the area) and the periphery, as well as total travel distance, were reported for the first 10 min of the test. Grooming behavior was reported for the last 15 min of the test.

### Y-maze test

The experimental setup included a three-arm, Y-shaped maze (LxWxH: 35 × 5 × 20 cm) with a 120° angle between the arms under a 100-lux overhead light. One of the arms was denoted as the start arm throughout all trials, where all mice were placed in the beginning of each trial. Mice were allowed to freely explore the Y-maze for 8 min, with all three arms open. Alternation behavior was defined as consecutive entries into each of the three arms without repetition. Maximum alternations (total arm entries − 2) were scored as an index of ambulatory activity in the Y-maze. The spontaneous alternation index was defined as the actual alternations divided by the maximum alternations (alternations/max. alternations) × 100.

### Elevated plus maze

The elevated plus maze consists of a maze 50 cm above the floor, formed in the shape of a plus (+) sign, with two arms having walls (closed arms), and two being without walls (open arms). The maze was homogeneously illuminated with overhead lighting at 100 lux. Each mouse was placed in the central square facing the open arm opposite the investigator and was allowed to freely explore the maze for 5 min. Time spent in the open arms, closed arms, and the center of the maze was measured.

### Light-dark box test

The experimental setup consisted of a two-compartment plexiglass arena (L x W × H: 44 × 27 x 27 cm) under a 250-lux overhead light. The lit compartment (L × W: 27 × 27 cm) was constructed of white plexiglass with no ceiling. The dark compartment (L × W: 18 × 27 cm) was made of black plexiglass, covered with a black plexiglass ceiling. A small opening (7.5 × 7.5 cm) in the bottom center of the wall between the two compartments allows access to both compartments. The mouse was placed in the dark compartment and allowed to freely explore the arena for 10 min. Latency to first enter the lit compartment and total duration of time spent in the lit compartment were measured.

### Three-chamber social interaction test

The experimental setup included a three-compartment plexiglass arena (LxWxH: 60 × 40.5 × 22 cm) under a 100-lux overhead light. Each mouse trial was run in three stages: habituation (5 min), social preference (10 min), and social novelty (10 min). At the beginning of each stage, the test mice were placed in the center chamber. During habituation, the mice were allowed to freely explore the three empty chambers. For social preference assessment, a strain-, age-, and sex-matched unfamiliar mouse was placed in a wired cage in one chamber while an empty similar cage was placed in the opposite chamber. Experimental mice were allowed to freely explore the three chambers for 10 min. The location of the cage mouse across trials was equally distributed. For social novelty assessment, a novel strain, age- and sex-matched unfamiliar mouse was placed in the previously empty cage, and the positions of the cages were swapped. Experimental mice were allowed to freely explore the three chambers for 10 min. Results are presented as the time spent sniffing in close vicinity and facing the cages. Results for social preference are reported for 10 min, while results for social novelty preference are reported for the initial 5 min of the session.

### Contextual fear conditioning

Fear conditioning was conducted using a standard operant box (Ugo Basile 46000 Fear Conditioning System) in two different contexts (A and B). Context A consisted of Plexiglas walls, a grid floor and was washed with 10% ethanol to provide a background odor; White noise at 55 dB provided an auditory background. Context B consisted of white and black striped walls, and a white Plexiglas floor; vanilla scent was applied to the conditioning chamber, and the white noise was turned off. On day 1, mice were placed in context A for 2 min before fear conditioning using 3 pairings of the conditioned stimulus (CS duration 30 s, 85 dB, 2 kHz) with a co-terminating unconditioned stimulus (US, 2 s foot-shock, 0.75 mA) at 1 min intervals. The mice remained in the training chamber for another 30 s before being returned to home cages. On day 2, mice were placed in context A for 5 min without exposure to the CS or US. On day 3, mice were placed in context B for 5 min before being exposed to the CS (1 min, 85 dB, 2 kHz).

### Statistical analysis

Statistical analyses were performed using GraphPad Prism version 10.2.0 (GraphPad Software, Inc.). The Shapiro–Wilk test was used to assess normal distributions, and the Brown-Forsythe test was used to check whether standard deviations (SDs) were significantly different across groups. When the datasets did not meet normality assumptions, non-parametric tests were used, including Mann–Whitney test and Kruskal–Wallis test with Dunn’s *post hoc* analysis. Details of statistical analysis, including tests, sample size, definition of center and dispersion, are found in the figure legends. Significance was determined using an alpha value of 0.05. For the cell culture experiments, each *n* represents a cell that was imaged. For mouse tissue sections and electrophysiology experiments, each *n* represents the average measure of a mouse that was included in the experiment. For β-gal density, at least 3 independent images taken from 2 coronal slices were used. For glutamatergic synapse density, at least 4 independent images taken from 2 to 3 coronal slices were used.

### Reporting summary

Further information on research design is available in the Nature Portfolio Reporting Summary linked to this article.

## Supplementary information

Supplementary Information

Reporting Summary

Transparent Peer Review file

## Source data

Source data

## Data Availability

The data that support the findings of this study are available within the article and its Supplementary Information file, or from the corresponding author upon request. The MS proteomics data have been deposited in the ProteomeXchange Consortium via the jPOST partner repository with the dataset identifiers **PXD079897** (https://proteomecentral.proteomexchange.org/cgi/GetDataset?ID=PXD079897) for Fig. 1A–D, **PXD079898** (https://proteomecentral.proteomexchange.org/cgi/GetDataset?ID=PXD079898) for Fig. 4E and **PXD079896** (https://proteomecentral.proteomexchange.org/cgi/GetDataset?ID=PXD079896) for Supplementary Fig. 12. Source data are provided with this paper.
