# Supplementary material for: Sexually dimorphic control of excitatory synapse maintenance in ventral hippocampal circuits by Neurexin2α ligand Vstm2b
Source: Nat Commun. 2026 Oct 5;17:11344. doi: 10.1038/s41467-026-78199-0 (PMC13728146; doi:10.1038/s41467-026-78199-0)
Supplement: Supplementary file 1 [file 41467_2026_78199_MOESM1_ESM.pdf]

# Supplementary Information

## Sexually Dimorphic Control of Excitatory Synapse Maintenance in Ventral Hippocampal Circuits by Neurexin2α Ligand Vstm2b

Nicolas Chofflet, Mathilde Chofflet, Mai Inagaki, Anthony John Pastore, Parisa Zarei, Manni Wang, Edan Shpigel, Maia Fukuyama-Kaplan, Martin Resua Rojas, Jeremie Van Prooijen, Ana Novacic, Daham Hettiarachchi, Shanghua Fan, Benjamin Feller, Nayoung Yi, Eric Lecuyer, Gabby Rudenko & Hideto Takahashi

This document contains:

- Supplementary Figures 1 to 20
- Uncropped blot images presented in Supplementary Figures

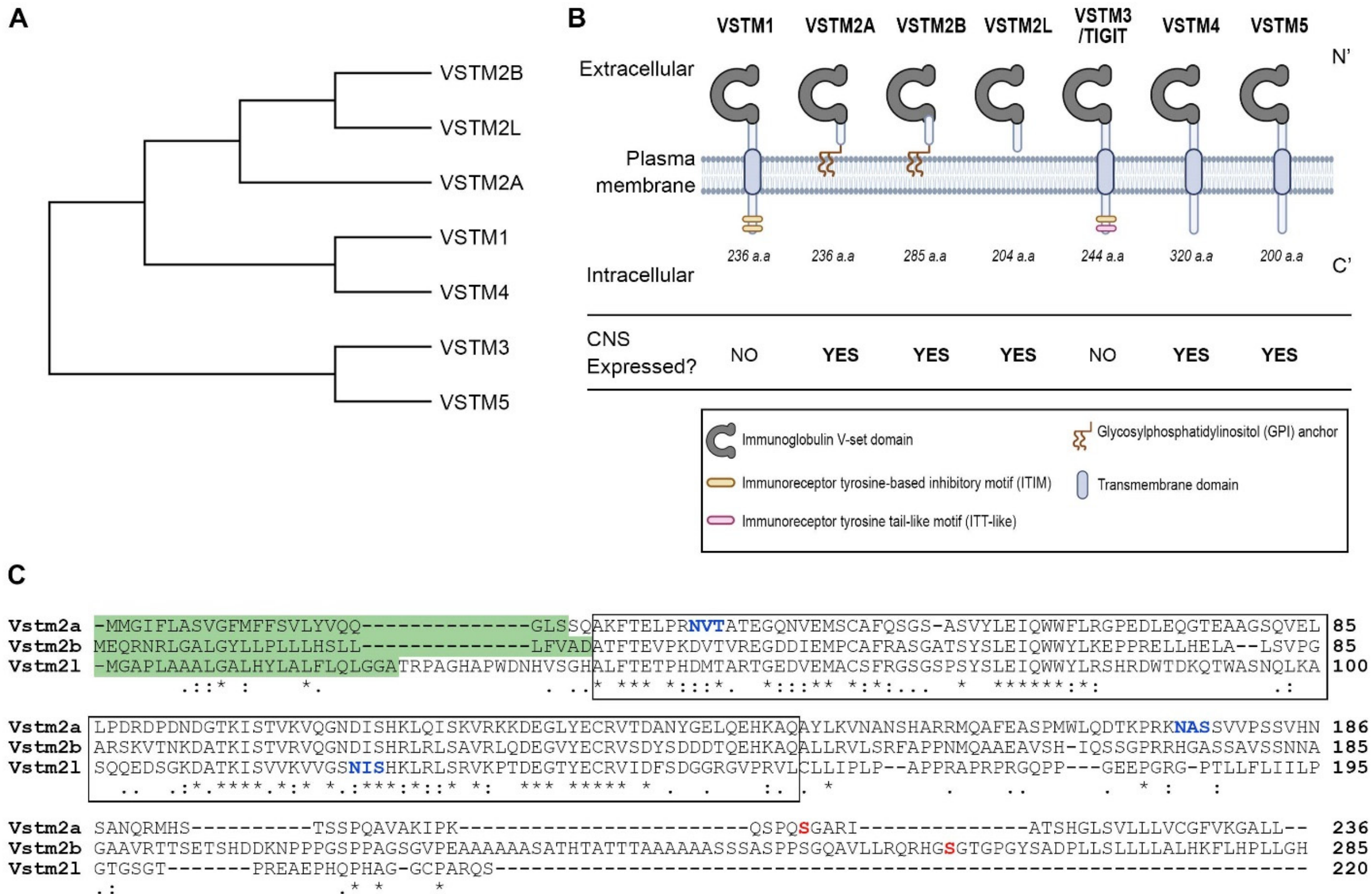


## Supplementary Figure 1: General characteristics of Vstm family members

**(A)** Phylogenetic tree of mouse Vstm family members.
**(B)** Predicted structure and expression of Vstm members in the mouse CNS. Cartoons, not to scale, show the putative mechanism by which each Vstm is tethered to the cell membrane. The presence of significant expression of each Vstm in the CNS is indicated as reported by either the Allen Brain Atlas or The Human Protein Atlas. Created in BioRender. Chofflet, N. (2026) https://BioRender.com/ipt24v2
**(C)** Sequence alignment of mouse Vstm2a (Uniprot ID: Q8R0A6), Vstm2b (Uniprot ID: Q9JME9) and Vstm2l (Uniprot ID: Q6PDS0). The signal peptides are highlighted in green, Ig-like domains are boxed, predicted N-linked glycosylation sites are shown in blue and predicted omega sites for GPI attachment are shown in red. Vstm2l appears to lack a GPI anchor attachment site and bears no obvious transmembrane segment. The degree of conservation is designated below as follows: * conserved, : semi conserved, . partially conserved.

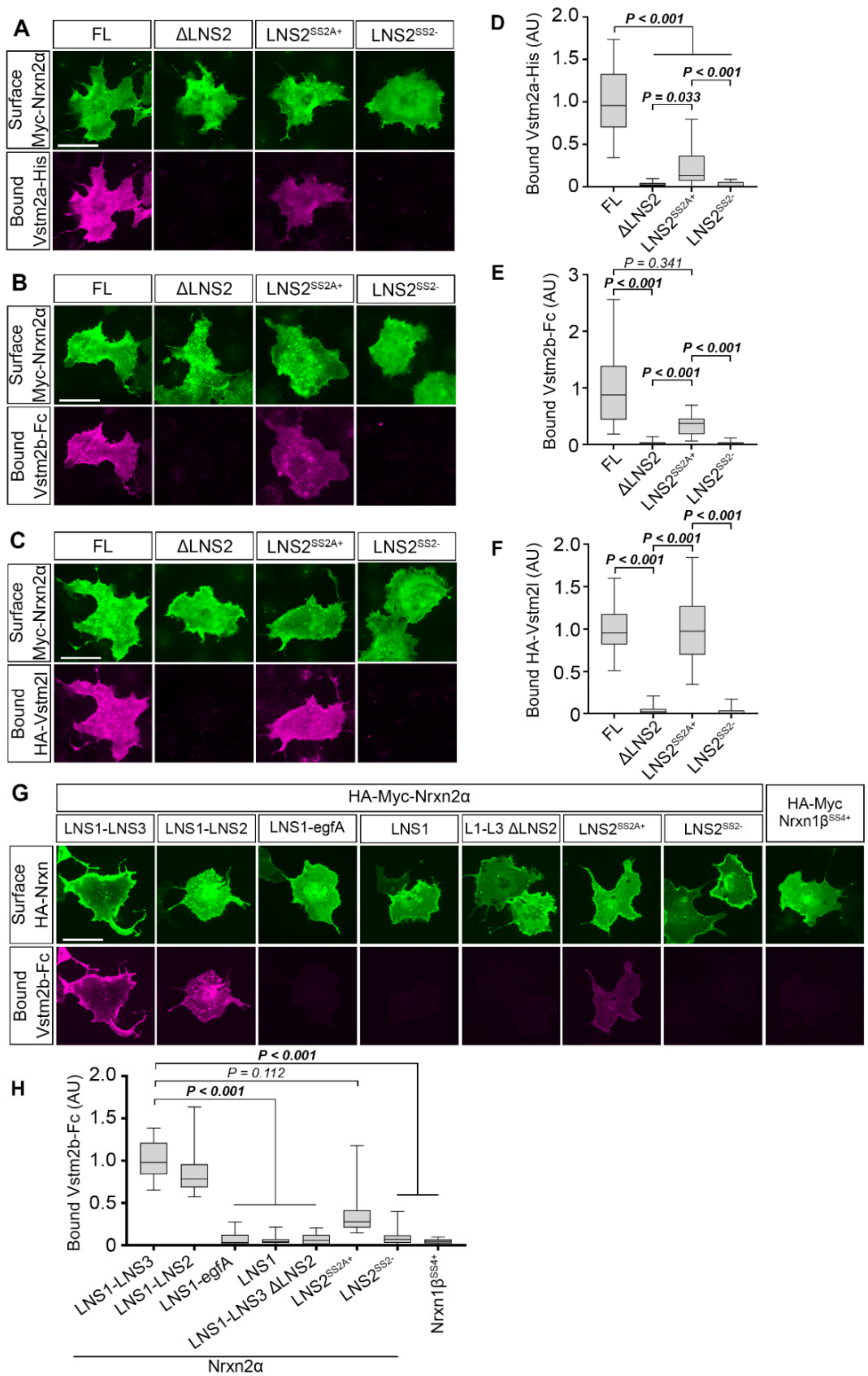


## Supplementary Figure 2: Vstm2 members interact with the second LNS (LNS2) domain of Nrxn2α in an SS2-dependent manner

**(A-C)** Immunofluorescence images of soluble Vstm2a-His (**A**), Vstm2b-Fc (**B**) and HA-Vstm2l

(**C**) bound to cell-surface-expressed Myc-Nrxn2α variants. FL: full length Nrxn2α$^{SS2A+}$, ΔLNS2: Nrxn2α lacking the LNS2 domain, LNS2$^{SS2A+}$ and LNS2$^{SS2-}$: membrane-anchored Nrxn2α LNS2 domain containing the SS2A insert or lacking the SS2 insert, respectively.

**(D-F)** Quantification of Vstm2a-His (**D**), Vstm2b-Fc (**E**) and HA-Vstm2l (**F**) bound to cell-surface-expressed Myc-Nrxn2α variants as described in (**A**-**C**). For Vstm2a, *n* = 33, 17, 30, and 30 cells; for Vstm2b, *n* = 32, 40, 22, and 35 cells; and for Vstm2l, *n* = 36, 29, 30, and 300 cells were analyzed for FL, ΔLNS2, LNS2$^{SS2A+}$, and LNS2$^{SS2A-}$, respectively.

**(G)** Immunofluorescence images of soluble Vstm2b-Fc bound to cell-surface-expressed HA-Myc-tagged Nrxn2α variants and Nrxn1β encoded in pDisplay cDNA vectors, in which the extracellular domains of Nrxns are tethered to the plasma membrane via the platelet-derived growth factor receptor (PDGFR) transmembrane domain. LNS1–LNS3: Nrxn2α LNS1 to LNS3 domains containing the SS2A insert; LNS1–LNS2: Nrxn2α LNS1 to LNS2 domains containing the SS2A insert; LNS1–egfA: Nrxn2α LNS1 domain fused to the egfA domain; LNS1: Nrxn2α LNS1 domain alone; LNS1–LNS3 ΔLNS2: Nrxn2α LNS1 to LNS3 domains lacking LNS2; LNS2$^{SS2A+}$ and LNS2$^{SS2-}$: membrane-anchored Nrxn2α LNS2 domain containing or lacking the SS2A insert, respectively.

**(H)** Quantification of Vstm2b-Fc bound to bound Nrxn2α variants and Nrxn1β as described in (**G**). *n* = 30 for each indicated construct. Statistical significance was examined by Kruskal-Wallis tests with Dunn's *post hoc* analysis (α = 0.05).

Data are presented as medians with interquartile range (IQR) and minimum to maximum values. Data were collected from three independent experiments. Scale bars, 50 μm.

A

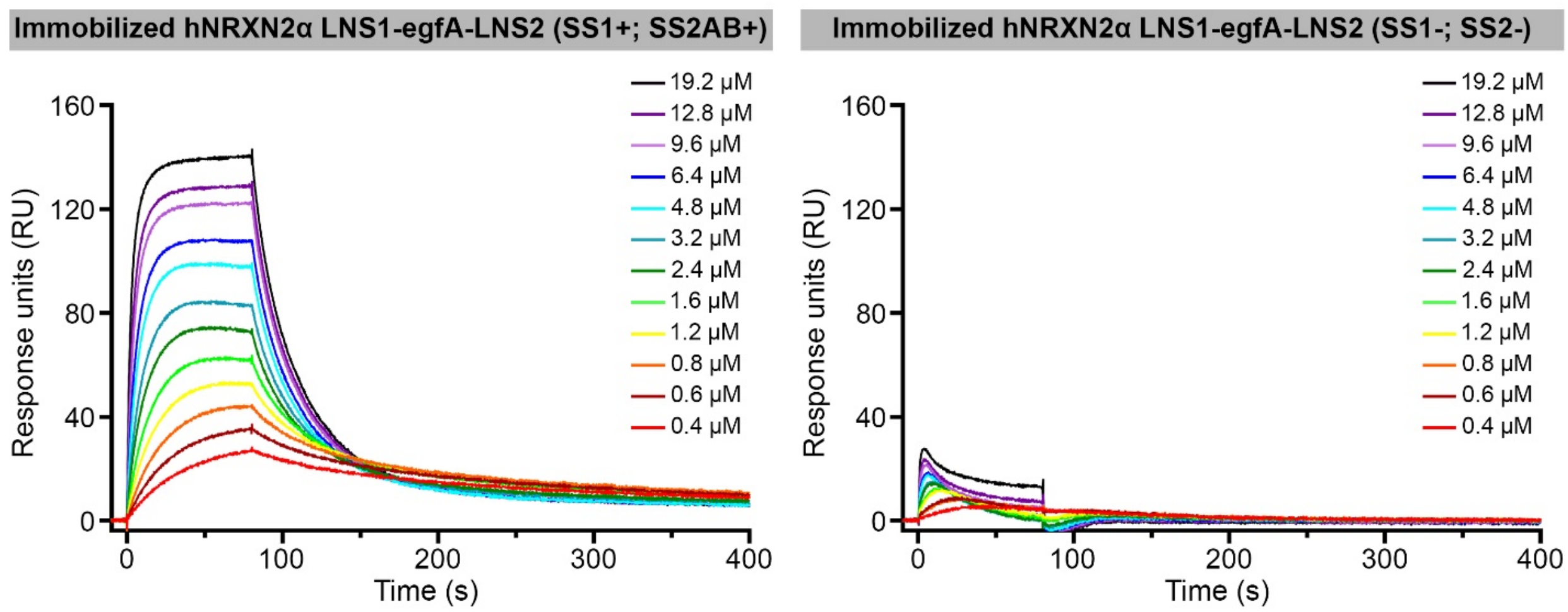


B

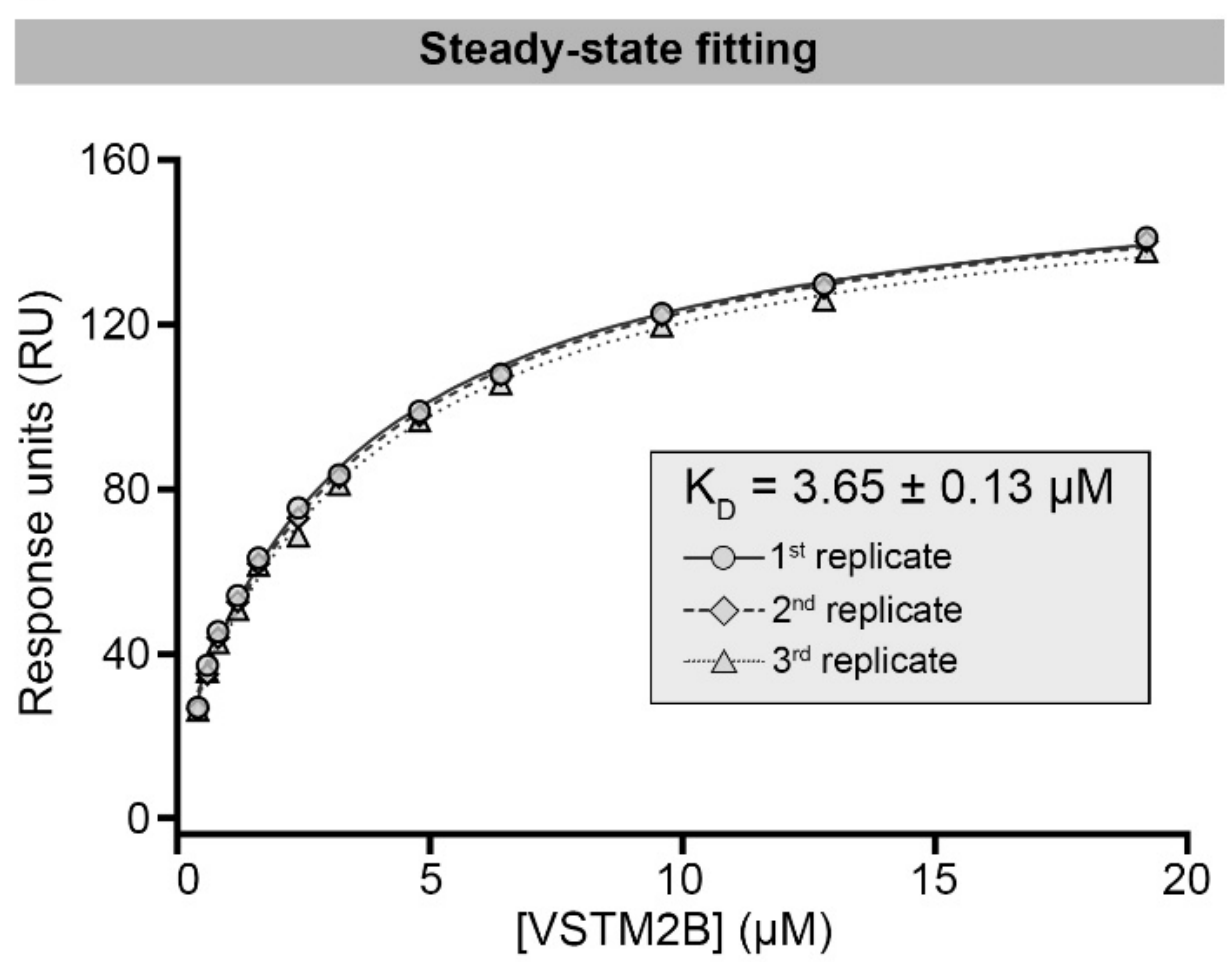


C

**CM5 chip parameters**

| Ligand | Immobilized response (RU) | Range (RU) | Chi² (RU²) | Max (RU) |
|---|---|---|---|---|
| hNRXN2α LNS1-egfA-LNS2 (SS1+; SS2AB+) | 1497.1 | 27 - 140 | 3.8 ± 0.7 | 146.3 ± 1.3 |
| hNRXN2α LNS1-egfA-LNS2 (SS1-; SS2-) | 1144.3 | 4 - 27 | N/A | N/A |

SS1 : E[244]EHPMEGPAHLTLNSEVGSL
LFSEGGAGRGGAGDVHQPTK[283]

SS2AB : H[385]AGIGHAMVNKLHYL[399]

## Supplementary Figure 3: Vstm2b binding to Nrxn2α LNS1-egfA-LNS2 splice forms measured by surface plasmon resonance

**(A)** Binding of the soluble, $(His)_6$-tagged Ig V-set domain of Vstm2b to immobilized Nrxn2a LNS1-egfA-LNS2 (SS1+; SS2AB+) (left) and Nrxn2a LNS1-egfA-LNS2 (SS1-; SS2-) (right)
**(B)** Steady-state fitting of the soluble, (His)6-tagged Ig V-set domain of Vstm2b binding to the Nrxn2α LNS1-egfA-LNS2 (SS1+; SS2AB+) biosensor chip. The experiment was carried out in triplicate on the biosensor, and each steady-state fitted binding curve is shown.
**(C)** Experimental parameters for the CM5 biosensor chips used.

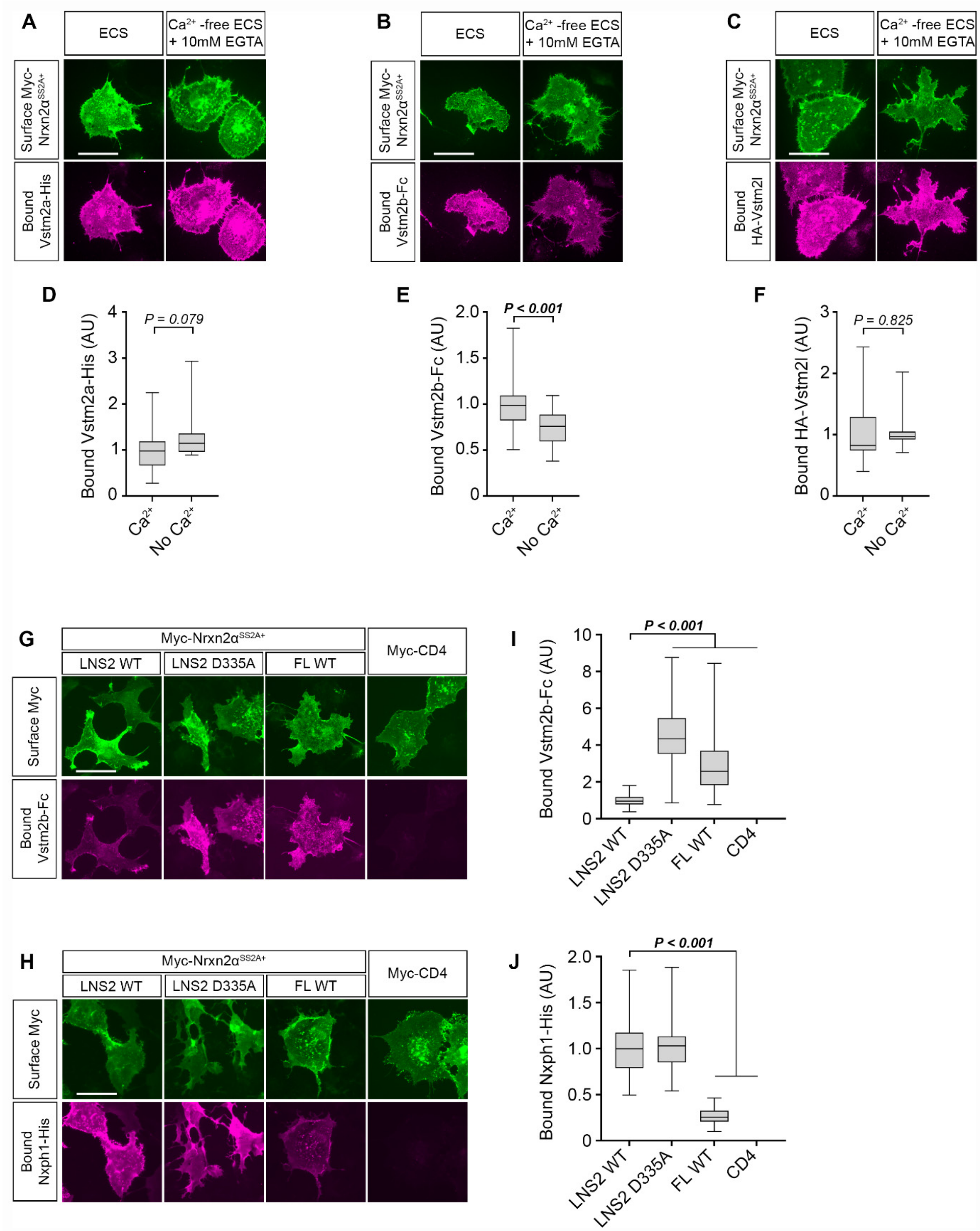


## Supplementary Figure 4: The interaction between Vstm2 family members and Nrxn2α does not require calcium

**(A-C)** Immunofluorescence images of soluble Vstm2a-His (**A**), Vstm2b-Fc (**B**) and HA-Vstm2l **(C)** bound to cell-surface-expressed Myc-Nrxn2α$^{SS2A+}$ under conditions with or without $Ca^{2+}$

and in the presence or absence of 10 mM EGTA.

**(D-F)** Quantification of Vstm2a-His (**D**), Vstm2b-Fc (**E**) and HA-Vstm2l (**F**) bound to Nrxn2α$^{SS2A+}$ as described in (**A**-**C**). For Vstm2a, *n* = 20 and 15 cells; for Vstm2b, *n* = 30 and 30 cells; and for Vstm2l, *n* = 35 and 14 cells were analyzed for $Ca^{2+}$ and no $Ca^{2+}$, respectively.

**(G-H)** Immunofluorescence images of soluble Vstm2b-Fc (**G**) and Nxph1-His (**H**) bound to Myc-tagged Nrxn2α$^{SS2A+}$ FL WT, LNS2$^{SS2A+}$ WT, LNS2$^{SS2A+}$ D335A (a $Ca^{2+}$-binding-deficient mutant) or CD4 as a negative control.

**(I-J)** Quantification of Vstm2b-Fc (**I**) and Nxph1-His (**J**) bound to Nrxn2α variants as described in (**G**-**H**).

Statistical significance was examined by using unpaired two-tailed t-tests (**D**-**F**) or Kruskal-Wallis tests with Dunn's *post hoc* analysis (**I**-**J**) ($\alpha = 0.05$). Data are presented as medians with interquartile range (IQR) and minimum to maximum values. Data were collected from three independent experiments except for (**F**) with two independent experiments. Scale bars, 50 μm.

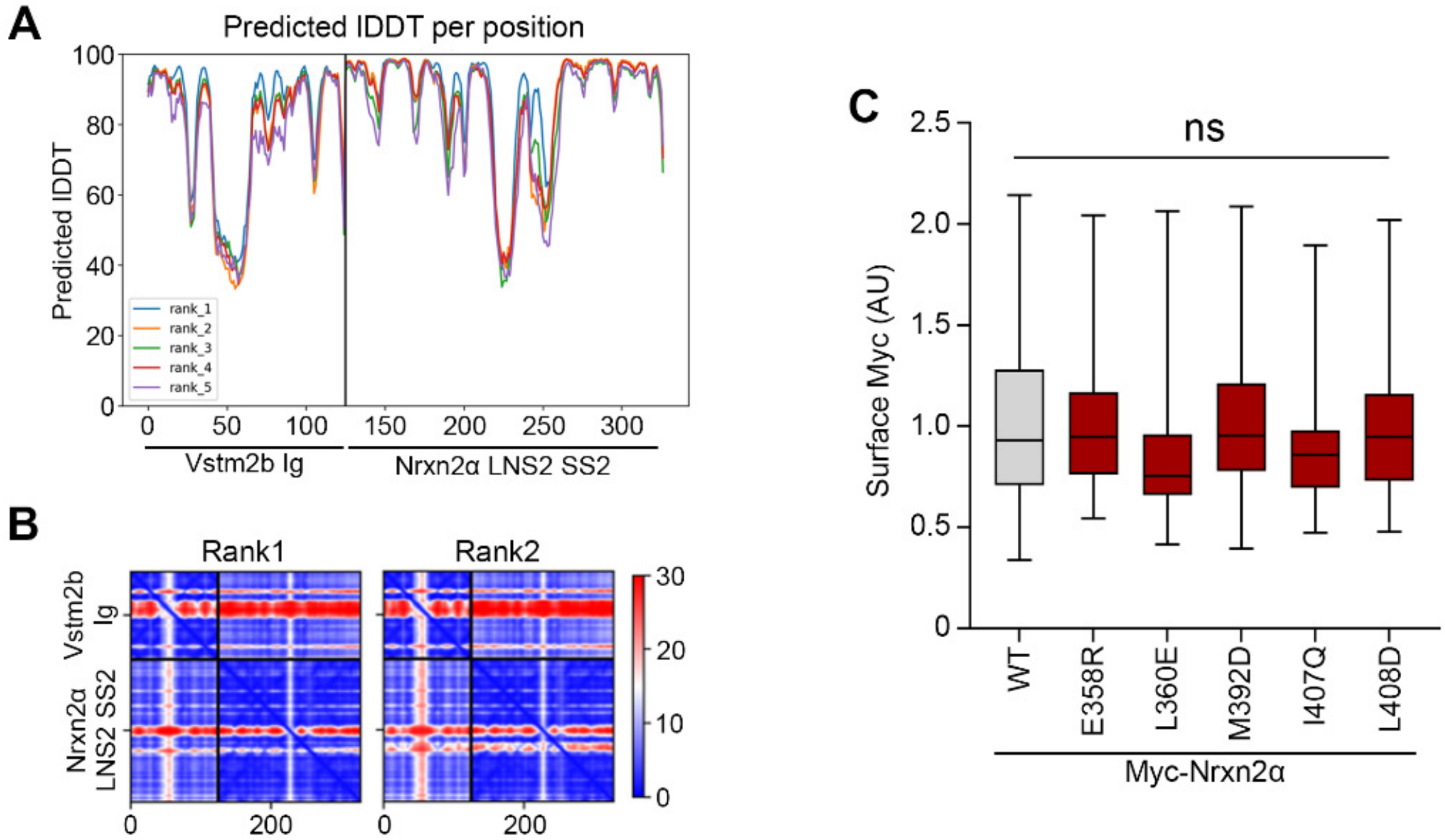


**Supplementary Figure 5: Structural predictions of the Vstm2b–Nrxn2α complex and surface expression analysis of Nrxn2α constructs**

**(A)** Predicted Local Distance Difference Test (pLDDT) results as a measure of prediction confidence for the top five models of the complex between the Vstm2b Ig and Nrxn2α LNS2$^{SS2A+}$ domains generated by ColabFold, a modified form of AlphaFold2. The rank 1 model (blue) with the highest pLDDT score was selected to guide our mutagenesis strategy.

**(B)** The inter Predicted Aligned Error (PAE) results as a measure of prediction confidence of the two highest ranked models of the Vstm2b Ig domain in complex with the Nrxn2α LNS2$^{SS2A+}$ domain. The PAE heatmap is shown with the indicated color scale and is represented in angstrom (0-30).

**(C)** Quantification of the expression of extracellularly Myc-tagged Nrxn2α$^{SS2A+}$ WT and mutants on the surface of COS-7 cells as described in main Figure 3G. Statistical significance was examined by Kruskal-Wallis test with Dunn's *post hoc* analysis ($\alpha = 0.05$). ns: not significant. Data are presented as medians with interquartile range (IQR) and minimum to maximum values. n = 76, 40, 44, 71, 41 and 38 cells were analyzed for WT, E358R, L360E, M392D, I407Q and L408D, respectively. Data were collected from three independent experiments.

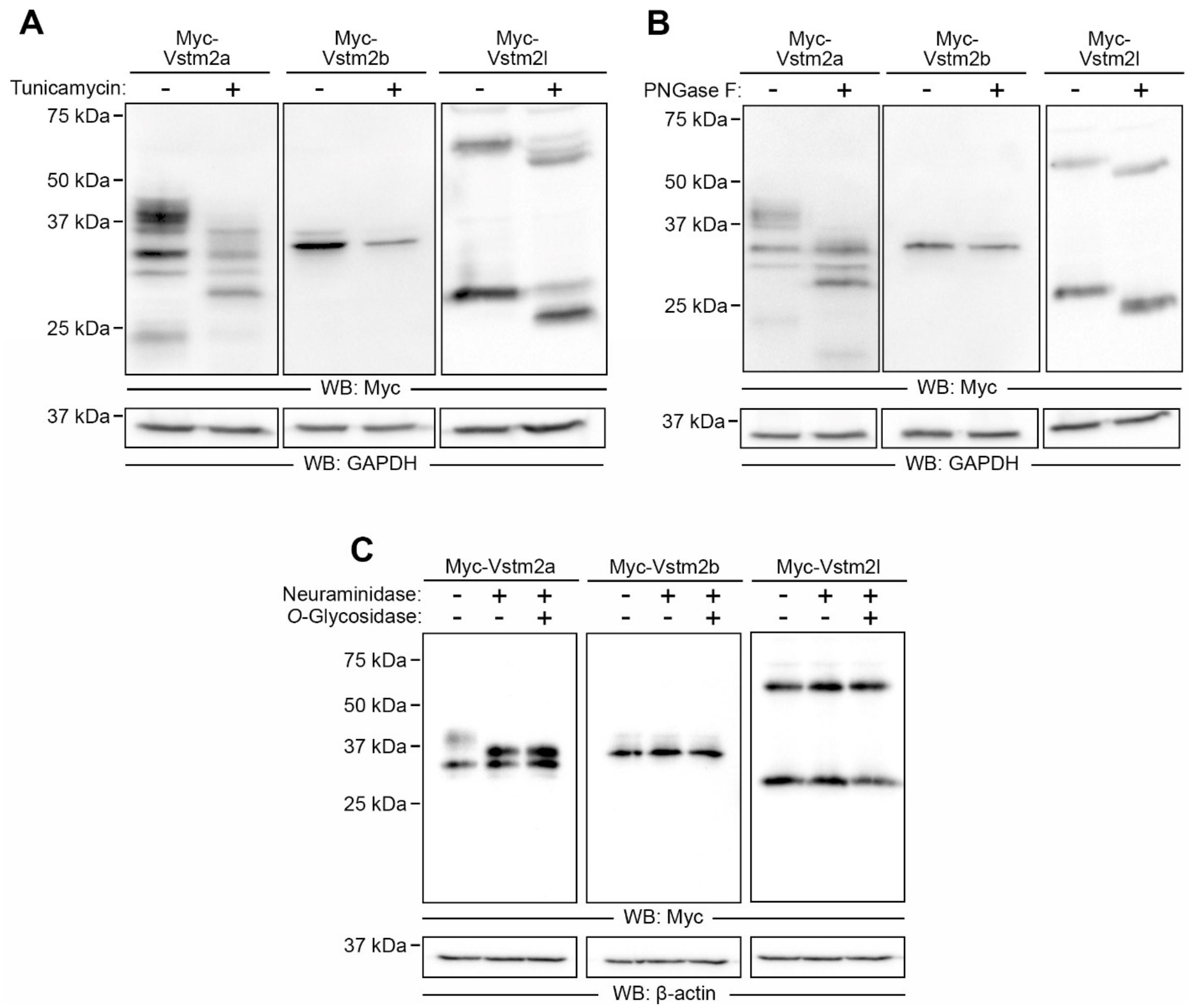


## Supplementary Figure 6: N-linked glycosylation of Vstm2a and Vstm2l and additional sialic acid modification of Vstm2a

**(A-C)** Immunoblots of cell lysates of HEK293T cells expressing Myc-tagged Vstm2a, Vstm2b or Vstm2l with or without treatment with tunicamycin, an inhibitor of N-glycosylation (**A**), PNGase F, an enzyme that removes N-linked oligosaccharides (**B**) or neuraminidase and O-glycosidase, enzymes that cleave sialic acid and O-glycans (**C**). Treatment with Tunicamycin or PNGase F severely affects the migration patterns of Vstm2a and Vstm2l, but not that of Vstm2b. Neuraminidase treatment resulted in a band shift of Vstm2a, but not Vstm2b or Vstm2l. Dual treatment with NA and O-glycosidase did not further affect the Vstm2a migration pattern nor did it change the apparent molecular weight of Vstm2b and Vstm2l upon SDS-PAGE. Similar results were obtained in three independent experiments for (A-B).

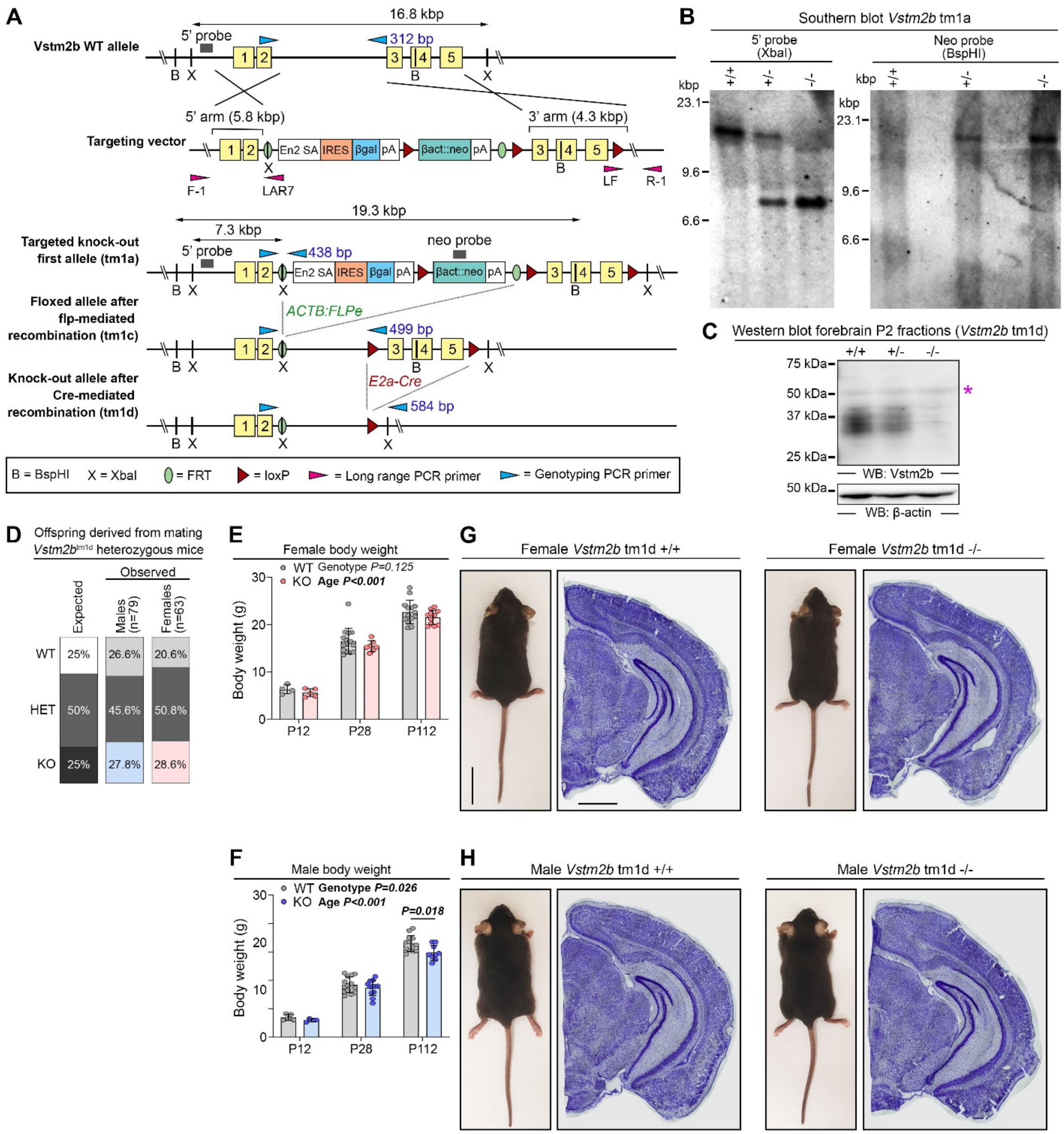


## Supplementary Figure 7: Generation and validation of *Vstm2b* mutant mouse lines

**(A)** Gene targeting strategy to generate *Vstm2b* mutant mouse lines by homologous recombination of a targeting vector containing the engrailed-2 splice acceptor (En2 SA) upstream of a β-gal reporter (LacZ) positioned before *Vstm2b* exons 3 to 5 which are flanked by LoxP sites. Subsequent flp- and Cre-mediated recombination results in generation of the tm1d allele in which *Vstm2b* exons 3 to 5 are excised. IRES, internal ribosome entry site; pA, SV40 polyadenylation site; βact::neo, neomycin-resistant gene cassette under the control of the β-actin promotor.

**(B)** Southern blot analysis of mouse genomic DNA extracted from *Vstm2b*$^{tm1a}$ WT, heterozygous and homozygous animals. Correct on-site homologous recombination without random insertion was verified using the 5' and neo probes.
**(C)** Immunoblots of mouse forebrain crude synaptosome fractions from *Vstm2b*$^{tm1d}$ wildtype, heterozygous, and homozygous mice following incubation with anti-Vstm2b antibody. β-actin was used as a loading control. The results simultaneously validated the loss of endogenous Vstm2b at the protein level and the specificity of the anti-Vstm2b antibody.
**(D)** Mendelian ratio of offspring derived from mating *Vstm2b*$^{tm1d}$ heterozygous mice.
**(E, F)** Body weight of female (**E**) and male (**F**) *Vstm2b*$^{tm1d}$ WT and homozygous KO mice at P12, P28, and P112. Data are shown as mean ± SEM, and each point represents the result from one mouse. Statistical significance was examined by two-way ANOVA with Sidak's *post hoc* analysis (α = 0.05). Sample numbers**:** female WT - P12 (*n* = 5), P28 (*n* = 14), P112 (*n* = 13); female KO - P12 (*n* = 4), P28 (*n* = 12), P112 (*n* = 9); male WT - P12 (*n* = 4), P28 (*n* = 15), P112 (*n* = 14); male KO - P12 (*n* = 5), P28 (*n* = 7), P112 (*n* = 11).
**(G, H)** Representative pictures of *Vstm2b*$^{tm1d}$ mice and their brain morphology by Nissl staining. Scale bars, 2 cm for mouse pictures and 1 mm for brain images. Similar results were obtained in three and two independent experiments for (**C**).

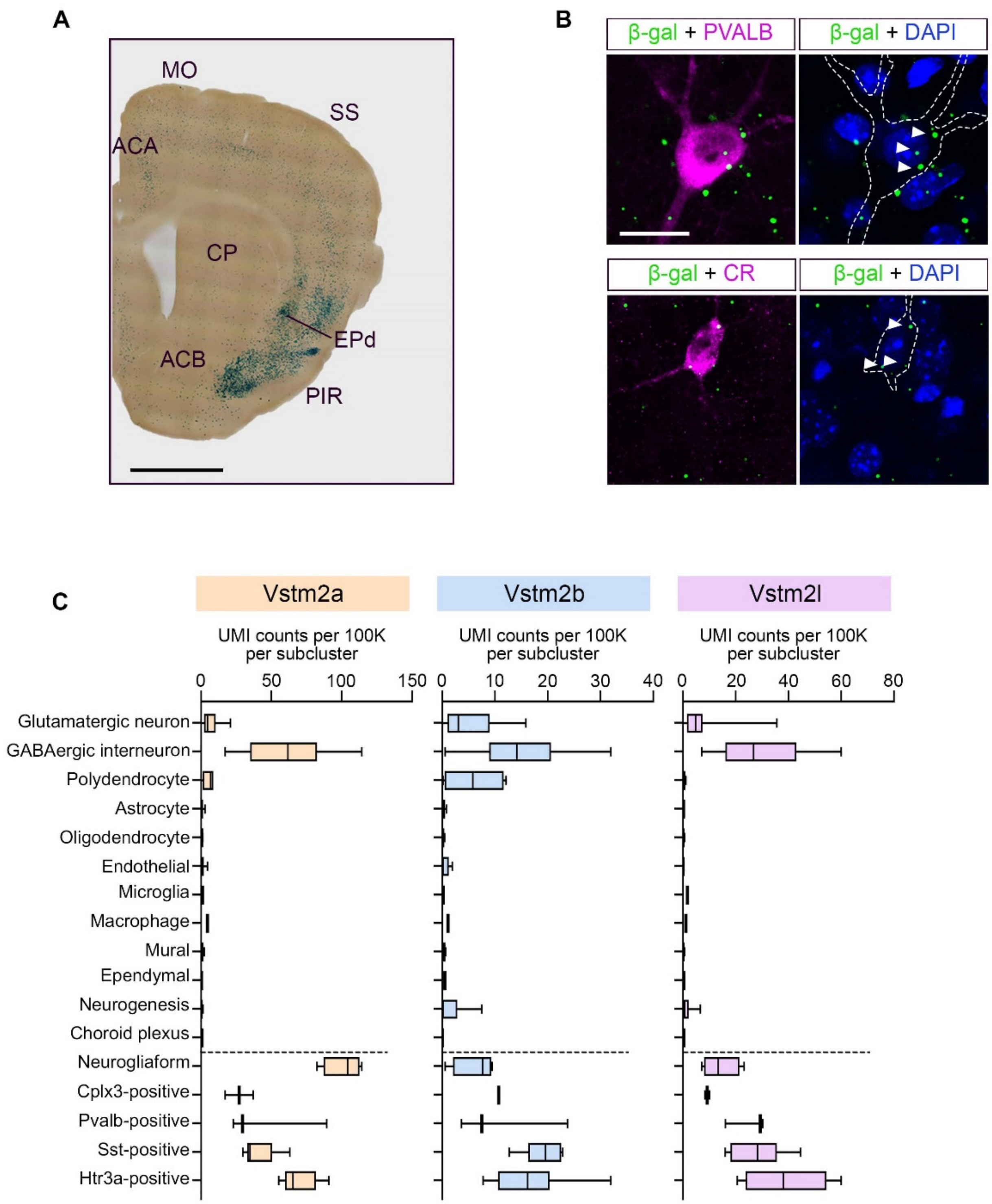


## Supplementary Figure 8: Region- and cell type-dependent expression patterns of Vstm2 members

**(A)** X-galactosidase staining of coronal brain sections from *Vstm2b*$^{tm1a}$ heterozygous mice (*Vstm2b* LacZ reporter mice) at P28. Scale bar, 2 mm. ACA, anterior cingulate cortex; ACB,

nucleus accumbens; CP, caudoputamen; EPd, endopiriform nucleus dorsal; MO, somatomotor areas; PIR, piriform area; SS, somatosensory areas.

**(B)** Representative immunofluorescence images of β-galactosidase (β-gal) in parvalbumin (PVALB)- and calretinin (CR)-expressing neurons in the ventral hippocampal CA1 of *Vstm2b* LacZ reporter mice at P28. Scale bar, 20 μm.

**(C)** Results of single-cell RNA sequencing of Vsmt2a (left), Vsmt2b (middle) and Vsmt2l (right) transcripts from the frontal cortex and hippocampus of adult male C57BL/6N mice (dataset from 42). Data are presented as box and whisker plots (min to max) of the absolute number of observed transcripts (UMI; unique molecular identifier) normalized by total number of reads per subcluster.

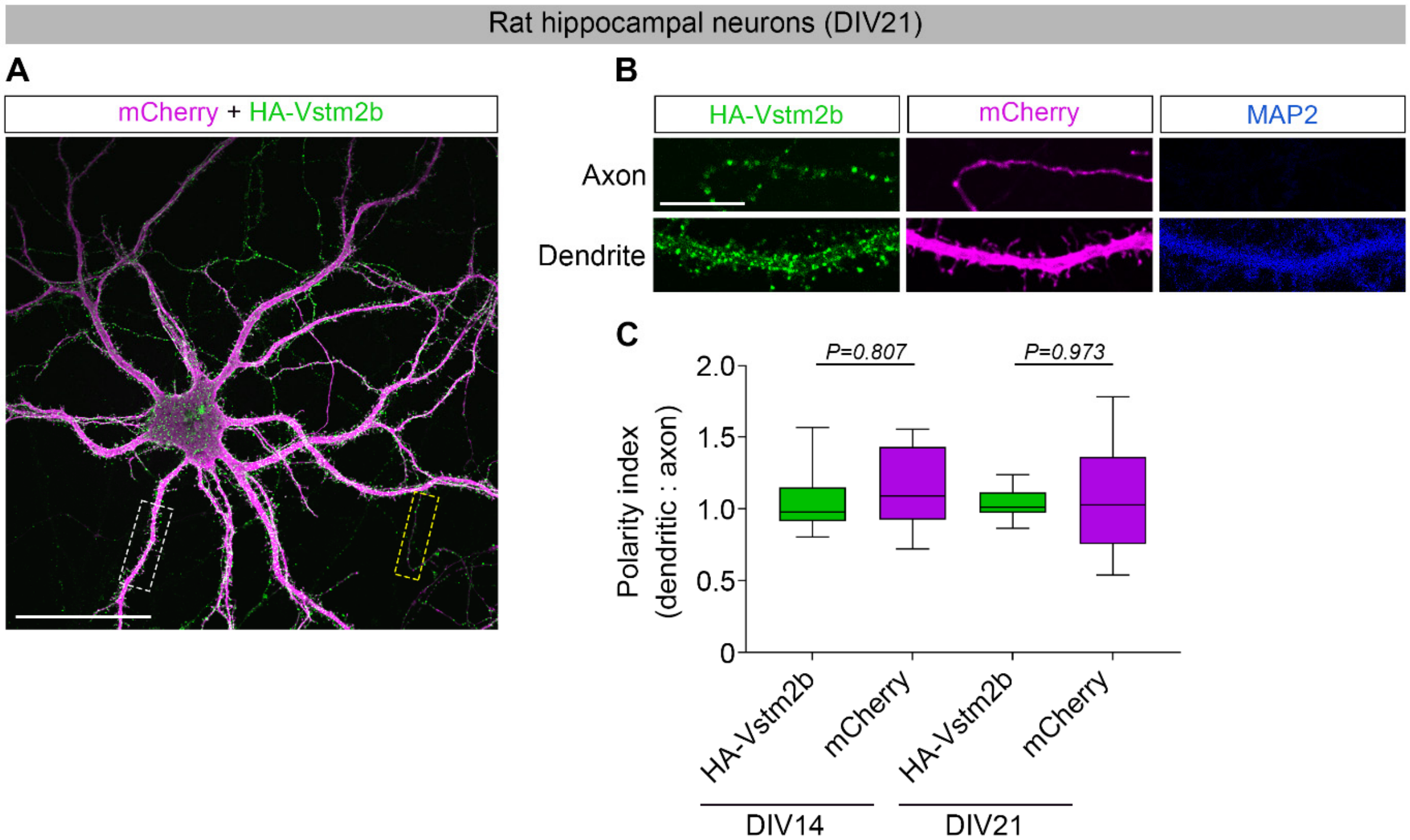


## Supplementary Figure 9: Subcellular localization of exogenous Vstm2b in cultured hippocampal neurons

**(A)** Representative fluorescence images of DIV21 rat hippocampal neurons immunostained for exogenous HA-Vstm2b and mCherry, and for the endogenous dendritic marker MAP2. Scale bar, 50 μm

**(B)** High-magnification images show the distribution of HA-Vstm2b in the dendritic and axonal compartments (dotted white and yellow boxes from (**A**), respectively). Scale bar, 10 μm.

**(C)** Quantification of the polarity index of HA-Vstm2b and mCherry as a measure of dendritic versus axonal distribution at 14 and 21 DIV. An index of 1 represents an equal distribution between the two compartments. Statistical significance was examined by two-way ANOVA with Sidak's *post hoc* analysis (α = 0.05). Data are presented as medians with interquartile range (IQR) and minimum to maximum values. *n* = 10 cells from three independent experiments.

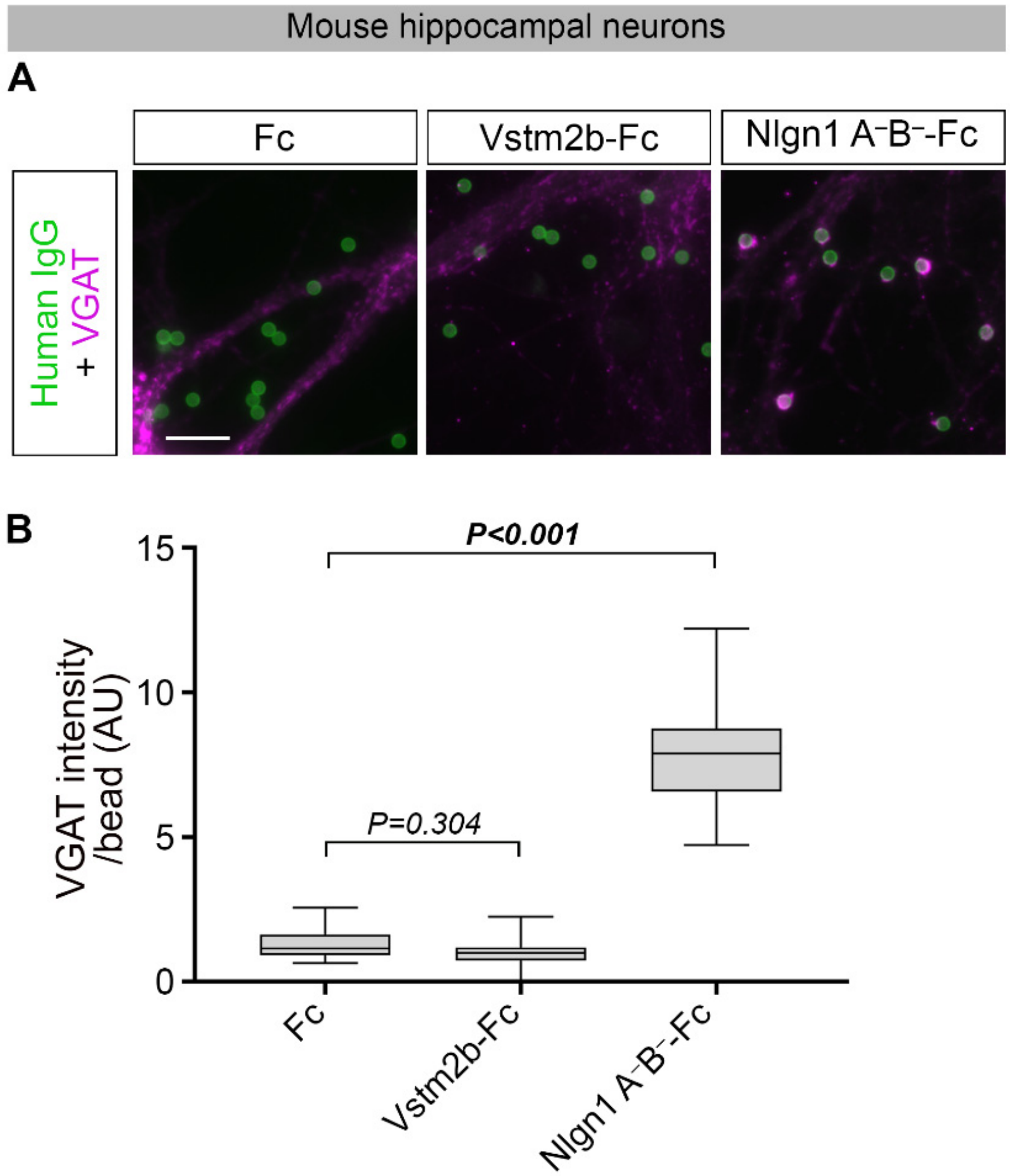


**Supplementary Figure 10: Vstm2b does not induce GABAergic presynaptic assembly in bead-based heterologous synapse formation assays**

**(A)** Representative fluorescence images of VGAT (magenta) in primary mouse hippocampal neurons incubated with Fc-, Vstm2b-Fc- or Nlgn1 $A^{-}B^{-}$-Fc-coated beads (green). Scale bar, 10 µm.

**(B)** Quantification of the average intensity of VGAT on beads as described in **A**. Statistical significance was examined using a Kruskal-Wallis test with Dunn's post hoc analysis ($\alpha = 0.05$). Data are presented as medians with interquartile range (IQR) and minimum to maximum values. *n* = 30 images with 10 beads averaged per image from three independent experiments.

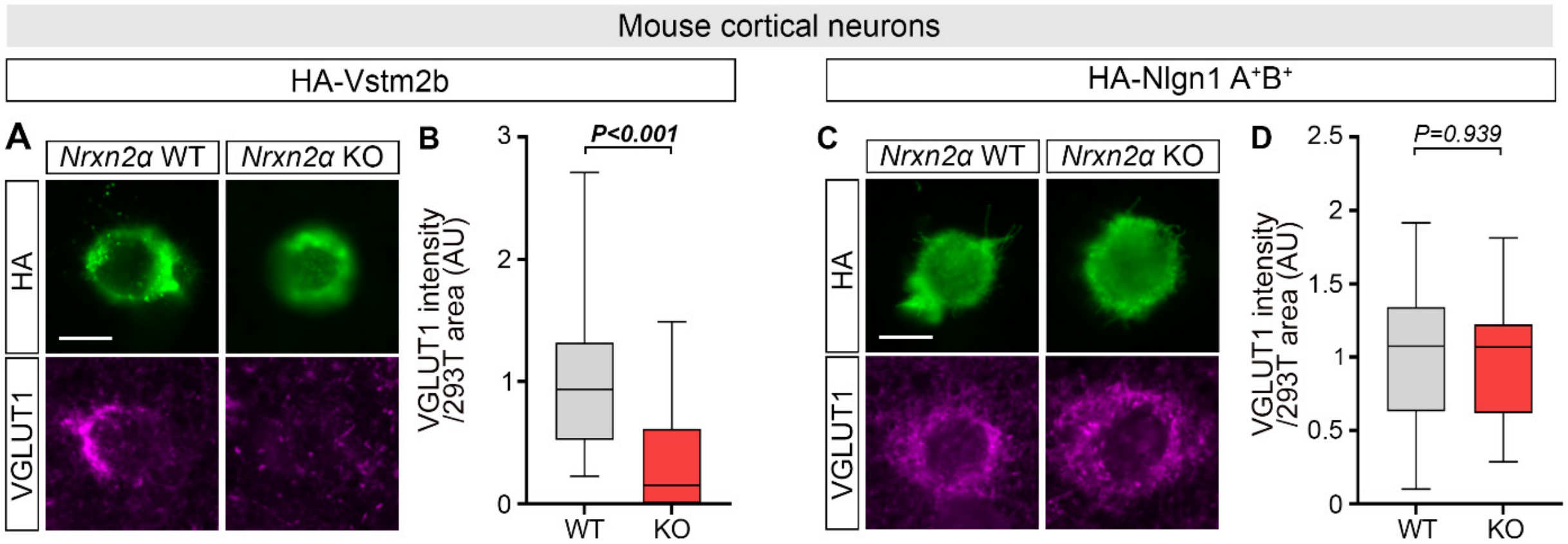


## Supplementary Figure 11: Vstm2b induces glutamatergic presynaptic assembly via Nrxn2α in HEK293T-based heterologous synapse formation assays

**(A, C)** Representative fluorescence images of VGLUT1 clustering induced by HEK293T cells expressing HA-Vstm2b (**A**) or HA-Nlgn1 $A^{+}B^{+}$ (**C**) cocultured with primary mouse cortical neurons derived from *Nrxn2α* wild-type (WT) and knockout (KO) littermates. Scale bars, 15 μm.

**(B, D)** Quantification of the integrated intensity of VGLUT1 accumulated on HEK293T cells expressing HA-Vstm2b (**B**) or HA-Nlgn1 $A^{+}B^{+}$ (**D**) as described in **A** and **C**. Statistical significance was examined with a Mann–Whitney test (B) or an unpaired two-tailed t-test (D) ($\alpha = 0.05$). Data are presented as medians with interquartile range (IQR) and minimum to maximum values. ns: not significant. For VGLUT1, $n$ = 23 (WT) and 18 (KO) cells were analyzed; for VGAT analyses, $n$ = 26 (WT) and 30 (KO) cells were analyzed. Data were collected from two independent experiments.

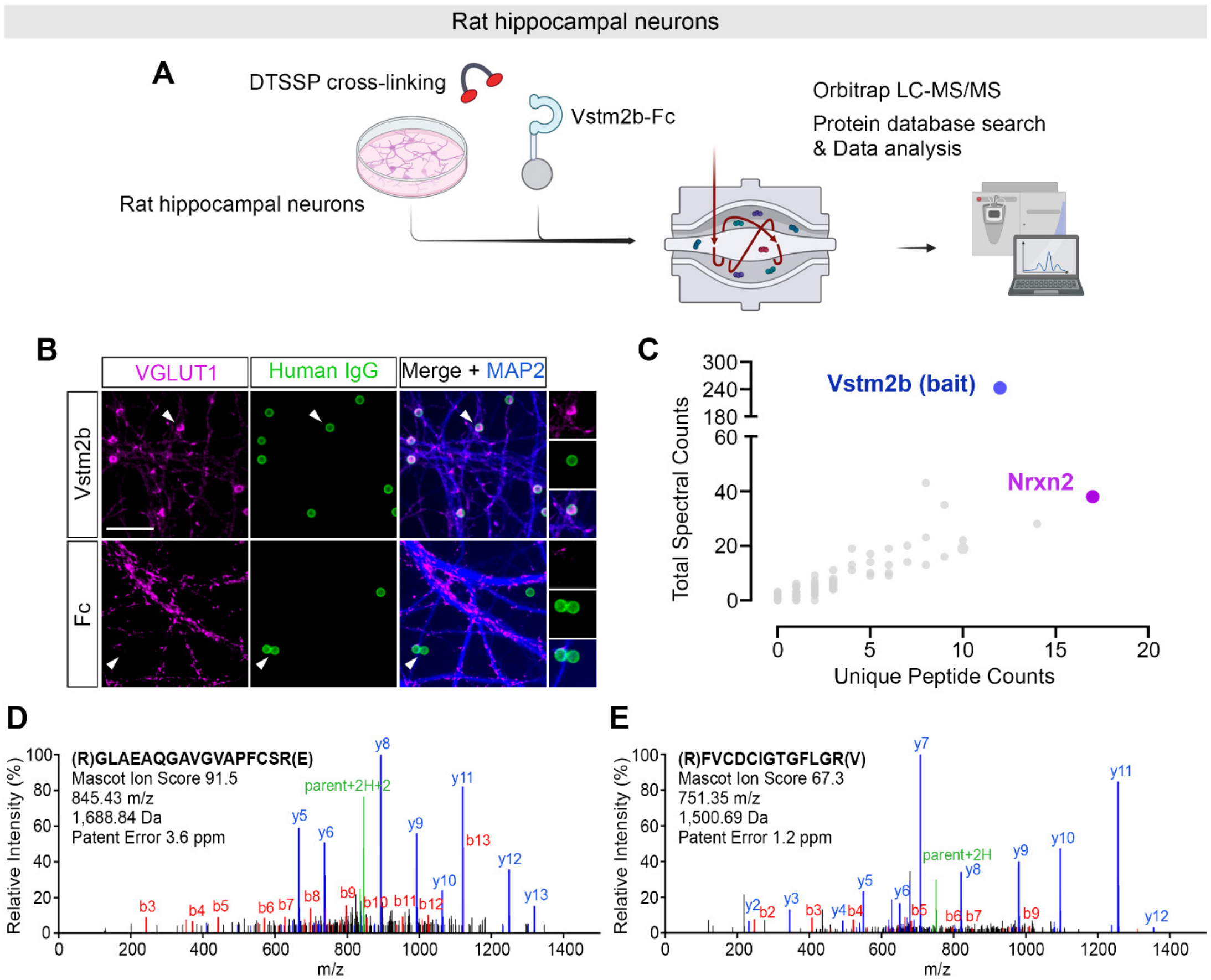


**F**

Nrxn2α (Q63374) - *Rattus norvegicus* (Rat)

MALGSRWRPP PQLPPLLLLL ALVAGVRGLE FGGGPGQWAR YARWAGAAST GELSFSLRTN ATRALLLYLD
DGGDCDFLEL LLVDGRLRLR FTLSCAEPAT LQLDTPVADD RWHMVLLTRD ARR**TALAVDG EAR**AAEVRSK
RREMQVASDL FVGGIPPDVR **LSALTLSTVK YEPPFR**GLLA NLKLGERPPA LLGSQGLRGA AADPLCAPAR
NPCANGGLCT VLAPGEVGCD CSHTGFGGKF CSEGEHPMEG PAHLTLNSPV GSLLFSEGGA GRGGAGDVHQ
PTKGKEEFVA TFKGNEFFCY DLSHNPIQSS TDEITLAFRT LQRNGLMLHT GKSADYVNLS LKSGAVWLVI
NLGSGAFEAL VEPVNGKFND NAWHDVRVTR NLRQHAGIGH AMVNKLHYLV TISVDGILTT TGYTQEDYTM
LGSDDFFYIG GSPNTADLPG SPVSNNFMGC LKDVVYKNND FKLELSRLAK EGDPKMK**LQG DLSFR**CEDVA
ALDPVTFESP EAFVALPRWS AKR**TGSISLD FR**TTEPNGLL LFSQGRRAGA GVGSHSSSQR ADYFAMELLD
GYLYLLLDMG SGGIKLRASS RKVNDGEWCH VDFQRDGRKG SISVNSRSTP FLATGESEVL DLESELYLGG
LPEGGRVDLP LPPEVWTAAL R**AGYVGCVRD LFIDGR**SRDL R**GLAEAQGAV GVAPFCSR**ET LKQCASAPCR
NGGICREGWN R**FVCDCIGTG FLGR**VCEREA TVLSYDGSMY MKIMLPNAMH TEAEDVSLRF MSQR**AYGLMM**
**ATTSR**ESADT LRLELDGGQM K**LTVNLDCLR** VGCAPSAAGK GPETLFAGHK LNDNEWHTVR VVRRGKSLQL
SVDNVTVEGQ MAGAHTRLEF HNIETGIMTE RRFISVVPSN FIGHLSGLVF NGQPYMDQCK DGDITYCELN
ARFGLR**AIVA DPVTFK**SRSS YLALATLQAY ASMHLFFQFK TTAPDGLLLF NSGNGNDFIV IELVKGYIHY
VFDLGNGPSL MKGNSDKPVN DNQWHNVVVS R**DPGNVHTLK** IDSRTVTQHS NGAR**NLDLKG ELYIGGLSK**N
MFSNLPKLVA SRDGFQGCLA SVDLNGRLPD LIADALHRIG QVERGCDGPS TTCTEESCAN QGVCLQQWDG
FTCDCTMTSY GGPVCNDPGT TYIFGKGGAL ITYTWPPNDR PSTRMDR**LAV GFSTHQR**SAV LVRVDSASGL
GDYLQLHIDQ GTVGVIFNVG TDDITIDEPN AIVSDGKYHV VRFTRSGGNA TLQVDSWPVN ERYPAGNFDN
ERLAIARQRI PYRLGRVVDE WLLDKGR**QLT IFNSQAAIK**I GGRDQGRPFQ GQVSGLYYNG LK**VLALAAES**
**DPNVRTEGHL R**LVGEGPSVL LSAETTATTL LADMATTIME TTTTMATTTT RRGRSPTMRD STTQNTDDLL
VASAECPSDD EDLEECEPST GGELILPIIT EDSLDPPPVA TRSPFVPPPP TFYPFLTGVG ATQDTLPPPA
ARRPSSGGPC QAERDDSDCE EPVEASGFAS GEVFDSSLPP TDDEDFYTTF PLVTDRTTLL SPRKPAPRPN
LRTDGATGAP GVLLAPSAPA PNLPAGKMNH RDPLQPLLEN PPLGPGVPTA FEPRRPPPLR PGVTSVPGFP
RLPTANPTGP GERGPPGAVE VIRESSSTTG MVVGIVAAAA LCILILLYAM YKYRNRDEGS YQVDQSR**NYI**
**SNSAQSNGAV VK**EKAPAAPK TPSKAKKNKD KEYYV

**Supplementary Figure 12: Mass spectrometry detection of endogenous Nrxn2α as an interactor of recombinant Vstm2b proteins applied onto primary hippocampal neurons**

**(A)** Experimental workflow to globally isolate Vstm2b-interacting proteins through affinity-

purification from cultured rat hippocampal neurons. Vstm2b-Fc-coated magnetic beads were incubated with rat hippocampal neurons for 24 h followed by cross-linking with membrane impermeable DTSSP. Proteins bound to the Vstm2b-Fc-coated beads were analyzed by label-free LC-MS/MS. Created in BioRender. Chofflet, N. (2026) https://BioRender.com/qiom0h9.

**(B)** Immunofluorescence images of Vstm2b-Fc-coated beads that induce VGLUT1 (magenta) clustering on contacting axons. Beads coated with Fc were used as a negative control. Dendrites were immunostained for MAP2 (blue), and Vstm2b-Fc beads were detected using anti-human IgG (green). Scale bar, 15 μm.

**(C)** Identification of endogenous Nrxn2α as a Vstmb2-Fc interactor by LC-MS/MS. The identified prey is represented as their total spectral counts as a function of unique peptide counts (one independent experiment, with Fc alone serving as the negative control, for a total of 2 analyzed samples).

**(D, E)** Spectra of the two peptides with the highest Mascot ion score unique to Vstm2b-Fc bait obtained by LC-MS/MS.

**(F)** Amino acid sequences of rat Nrxn2α peptides (red) identified by LC-MS/MS following pulldown of Vstm2b and associated protein complexes. The sequences underlined are shared between Nrxn2α and Nrxn2β isoforms. The red sequences represent all peptides detected in the experiment; no Nrxn2β-specific peptides were identified.

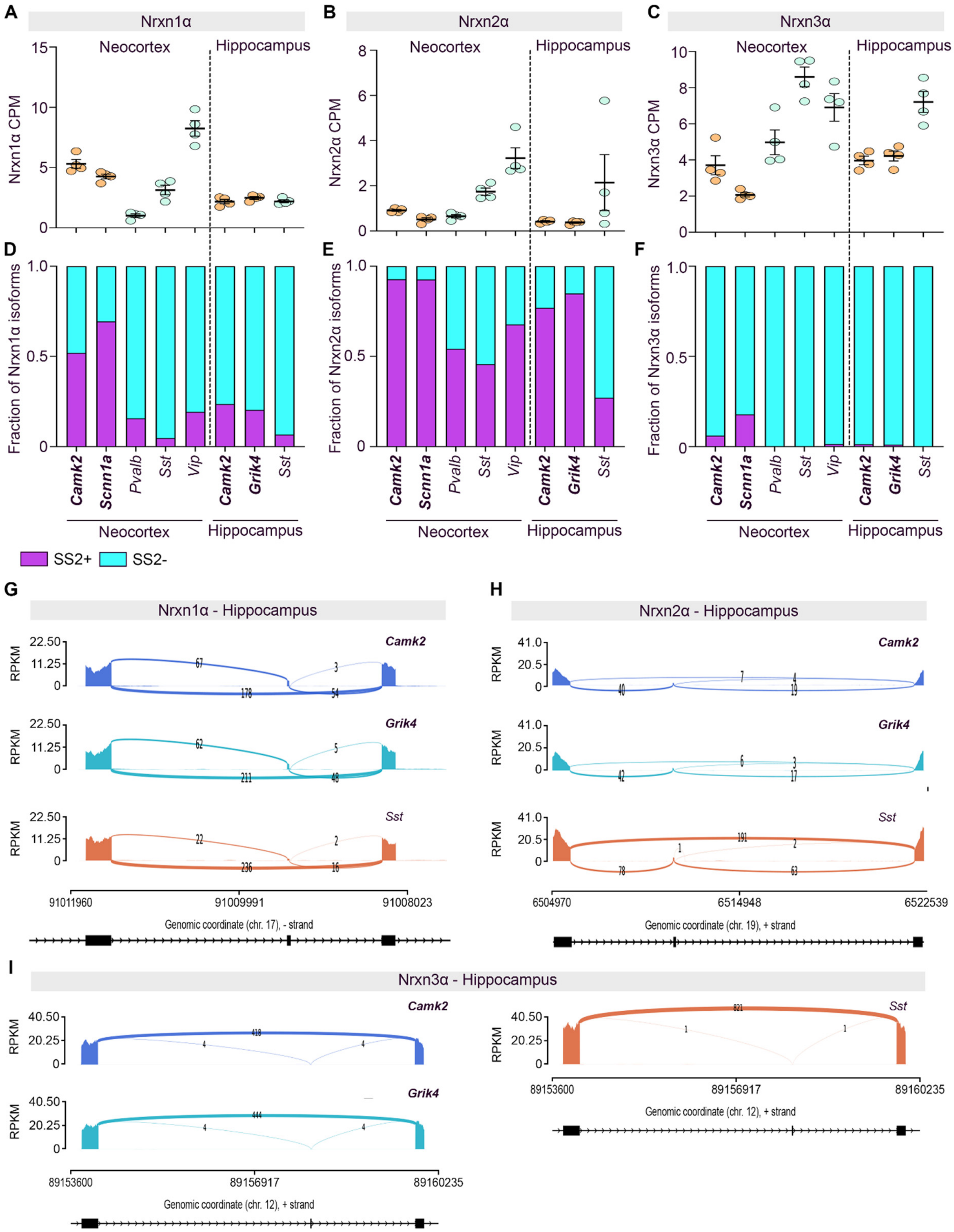


## Supplementary figure 13: Predominant expression of SS2-containing Nrxn2α isoforms in glutamatergic neurons

**(A-C)** Expression of Nrxn1α (**A**), Nrxn2α (**B**) and Nrxn3α (**C**) displayed as counts per million

(CPM) in different mouse neocortical and hippocampal cell types (dataset from 44). Orange and cyan ovals represent glutamatergic and GABAergic cell types, respectively. The x-axis represents the different cell types shown in panels **D–F**. Data are shown as mean ± SEM.

**(D-F)** Fractions of Nrxn1α (**D**), Nrxn2α (**E**) and Nrxn3α (**F**) isoforms that contains the SS2 insert corresponding to either splice variant A or AB for each cell type.

**(G-I)** Sashimi plots illustrating the read distribution and splice junctions at the SS2 insert for Nrxn1α (**G**), Nrxn2α (**H**) and Nrxn3α (**I**) in Camk2- and Grik4-positive glutamatergic and Sst-positive GABAergic neurons in the mouse hippocampus. Genomic coordinates, chromosome number and strand are indicated underneath the plots.

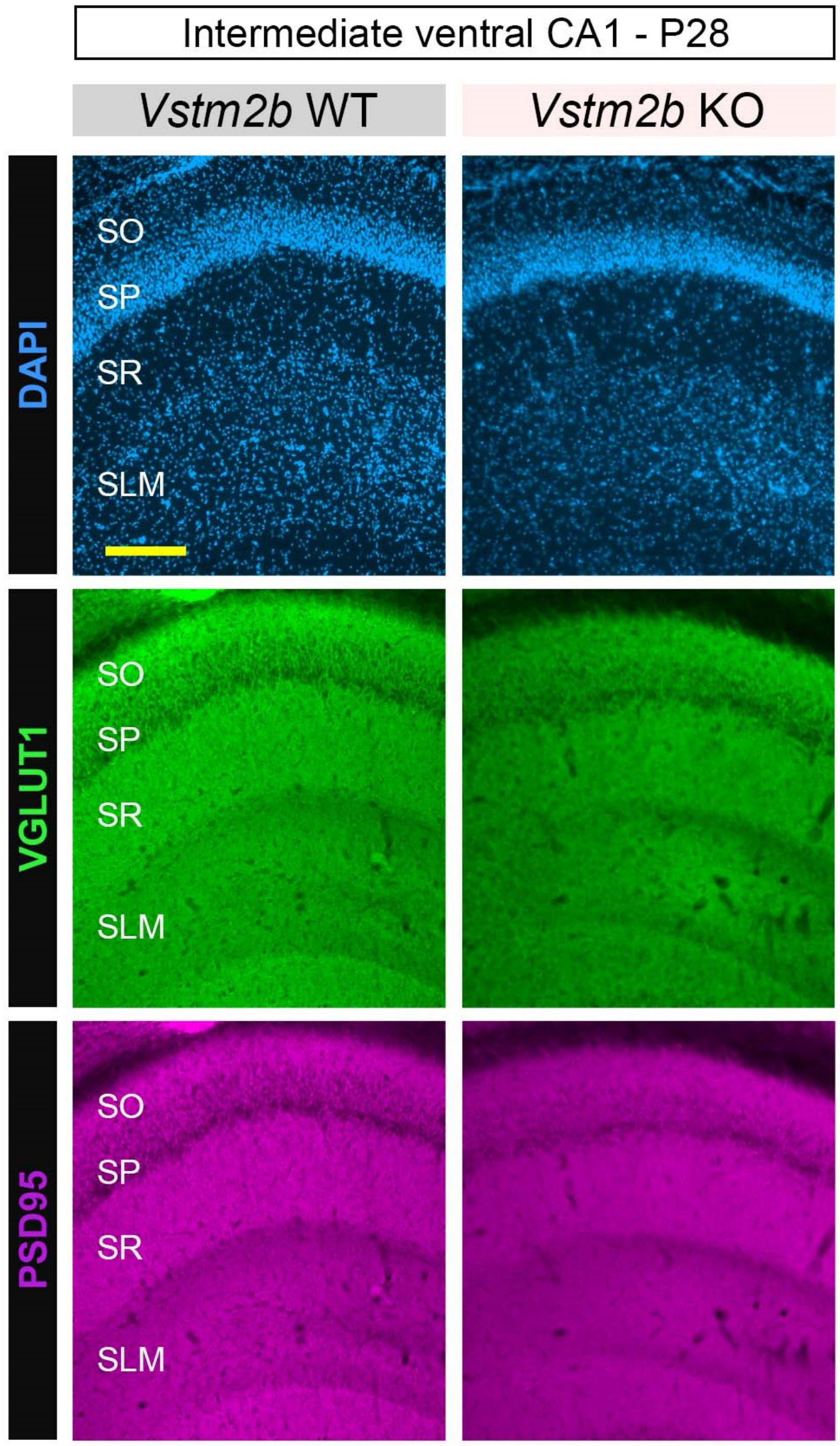


**Supplementary Figure 14: Excitatory synaptic marker immunoreactivity and laminar organization in the ventral hippocampal CA1 of *Vstm2b* KO mice**

Low-magnification confocal immunofluorescence images of the intermediate ventral hippocampal CA1 from female *Vstm2b* WT and KO mice at P28. Cryosections were labeled with DAPI, VGLUT1, and PSD95. No overt differences in marker immunoreactivity or laminar architecture were detected between genotypes. Scale bar, 200 µm. SO, stratum oriens; SP, stratum pyramidale; SR, stratum radiatum; SLM, stratum lacunosum-moleculare.

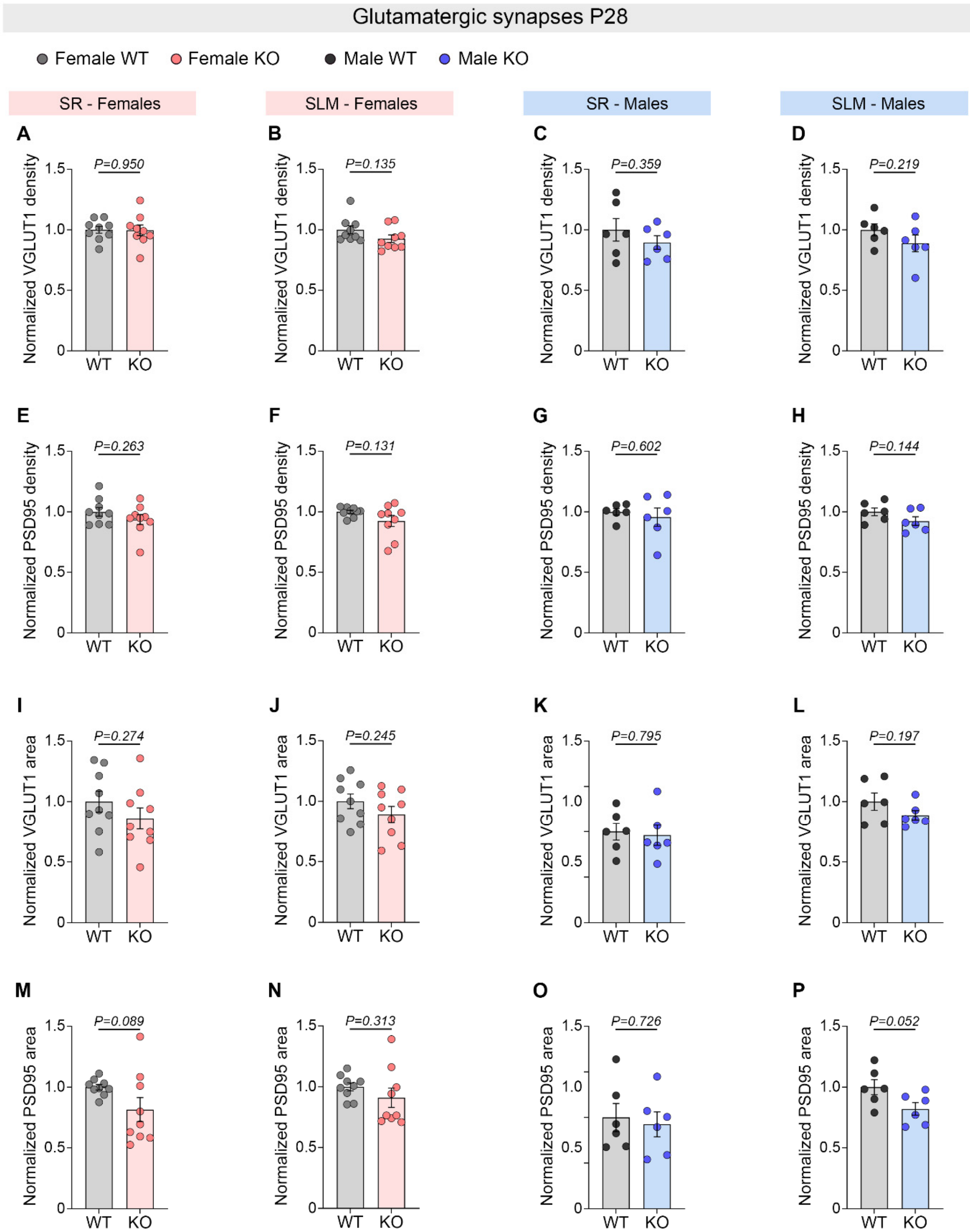


**Supplementary Figure 15: Quantification of VGLUT1 and PSD95 puncta in the ventral hippocampus of *Vstm2b* WT and KO mice at P28**

**(A-H)** Quantification of the density of VGLUT1 (**A-D**) and PSD95 (**E-H**) puncta in the ventral

hippocampal SR and SLM regions of *Vstm2b* WT and KO female and male mice at P28.
**(I-P)** Quantification of the area of VGLUT1 (**I-L**) and PSD95 (**M-P**) puncta in the ventral hippocampal SR and SLM regions of *Vstm2b* WT and KO female and male mice at P28. Statistical significance was determined by unpaired two-tailed *t*-tests ($\alpha = 0.05$). Data are shown as mean ± SEM, and each point represents the averaged result from one mouse; $n = 9$ female WT mice; $n = 9$ female KO mice; $n = 6$ male WT mice; $n = 6$ male KO mice.

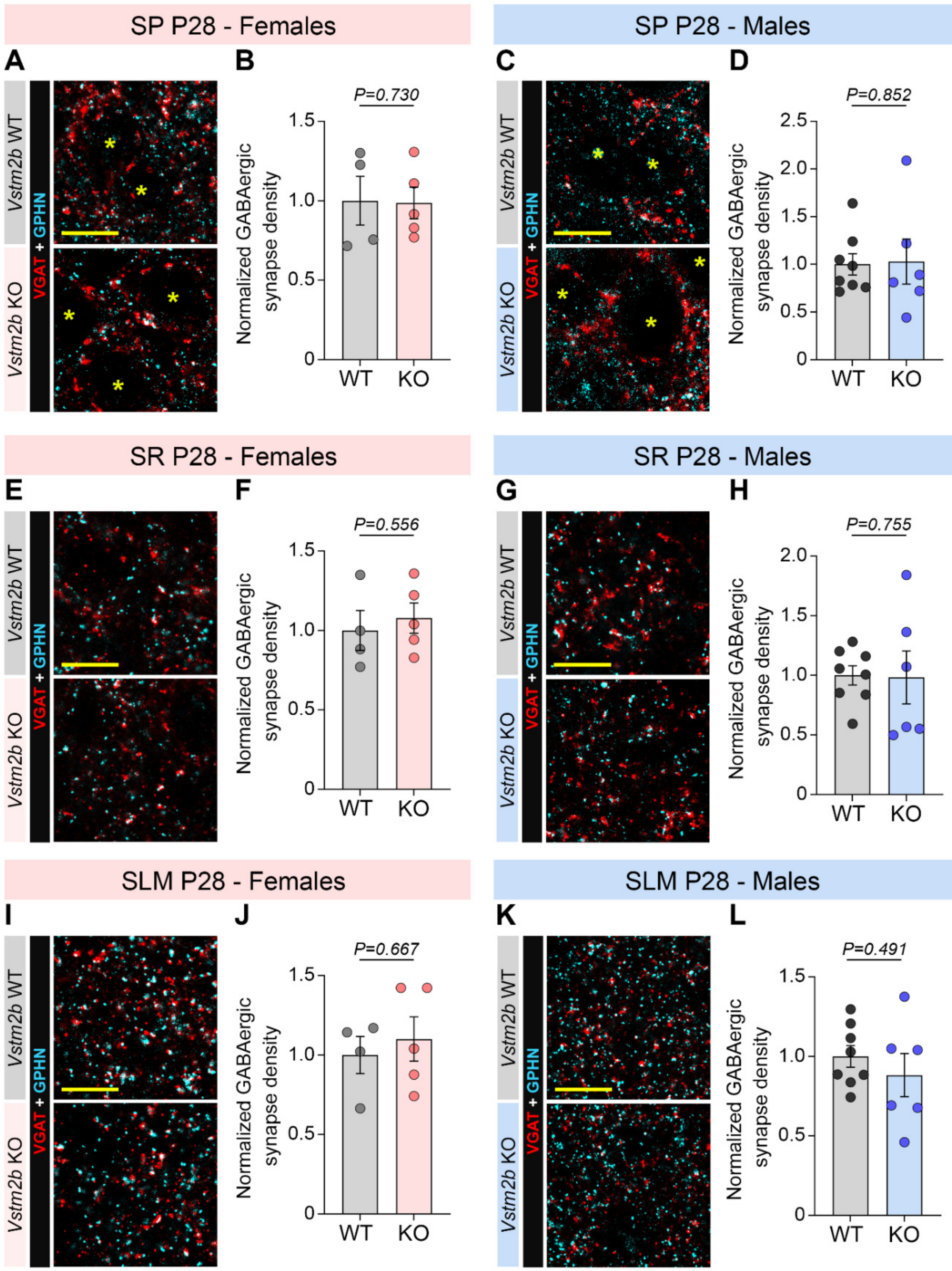


**Supplementary Figure 16: Quantification of GABAergic synapse density in the ventral CA1 of *Vstm2b* WT and KO mice at P28**

High-magnification immunofluorescence images of VGAT and Gephyrin (GPHN) and quantification of VGAT–GPHN colocalization puncta, corresponding to GABAergic synapses,

in the vCA1 SP of female (**A**, **B**) and male (**C, D**), SR of female (**E, F**) and male (**G**, **H**), and SLM of female (**I**, **J**) and male (**K**, **L**) *Vstm2b* WT and KO mice at P28. Yellow asterisks indicate nuclei in the SP. Scale bars, 5 μm. Statistical significance was determined by using Mann–Whitney tests (α = 0.05). Data are shown as mean ± SEM, and each point represents the averaged result from one mouse; *n* = 4 female WT mice, *n* = 5 female KO mice, *n* = 8 male WT mice, and *n* = 6 male KO mice.

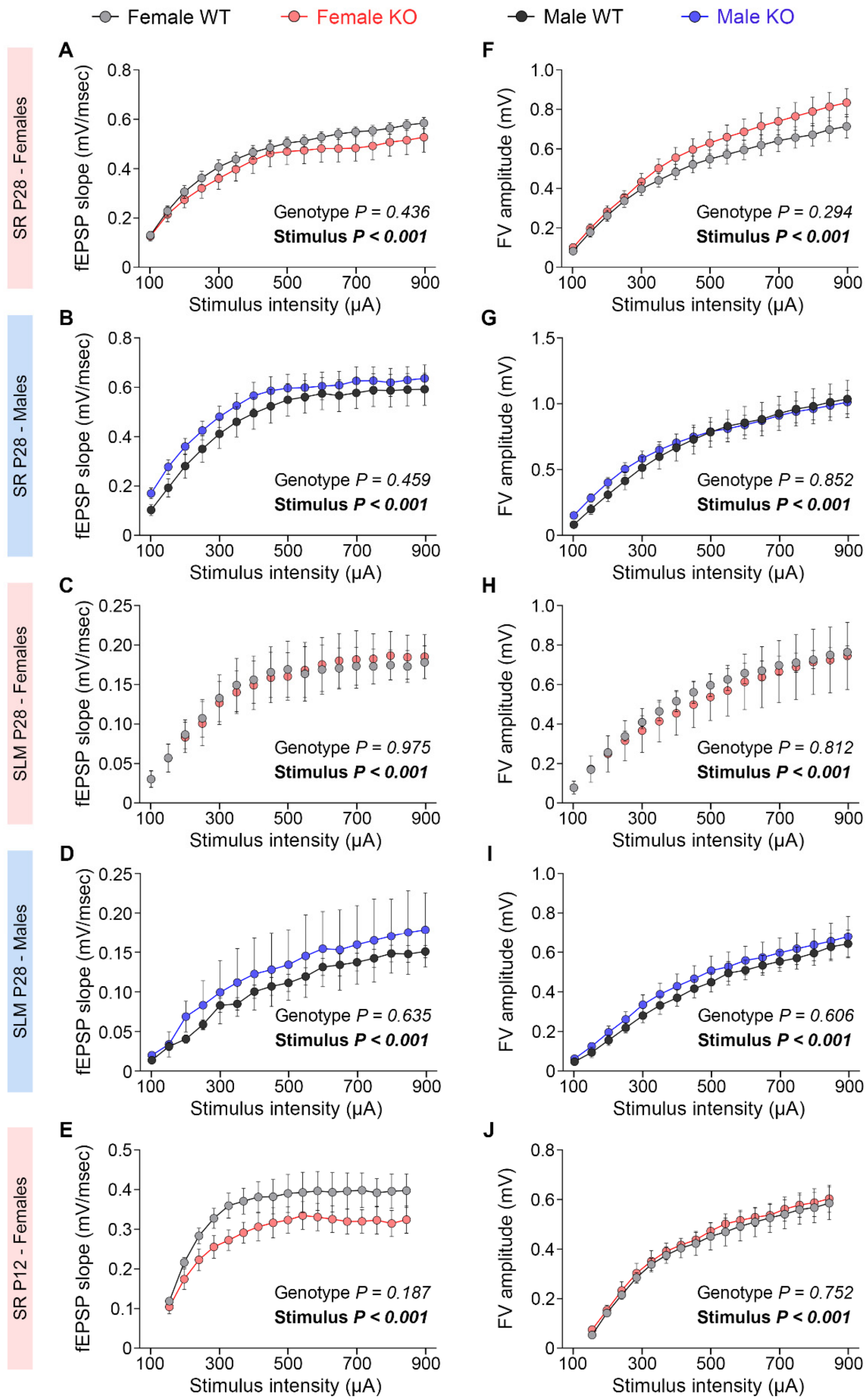


## Supplementary Figure 17: Input/output curves in the ventral hippocampus of *Vstm2b* WT and KO mice at P28 and P12

**(A-E)** fEPSP slope produced by different stimulus intensities at P28 and P12 in the SR and

SLM for *Vstm2b* WT and KO mice.

**(F-J)** Fiber volley amplitude elicited by different stimulus intensities at P28 and P12 in the SR and SLM for *Vstm2b* WT and KO mice.

Statistical significance was assessed by two-way RM ANOVA (α = 0.05). Sample numbers for the SC-vCA1 synapses at P28 (**A**, **B**, **F**, and **G**): *n* = 7 mice (24 recordings) for female WT; *n* = 7 mice (25 recordings) for female KO; *n* = 3 mice (12 recordings) for male WT; *n* = 4 mice (17 recordings) for male KO. Sample numbers for the TA-vCA1 synapses at P28 (**C**, **D**, **H**, and **I**): *n* = 3 mice (9 recordings) for female WT; *n* = 3 mice (9 recordings) for female KO; *n* = 3 mice (12 recordings) for male WT; *n* = 3 mice (11 recordings) for male KO. Sample numbers for the SC-vCA1 synapses at P12 (**E** and **J**): *n* = 4 mice (15 recordings) for female WT; *n* = 4 mice (16 recordings) for female KO.

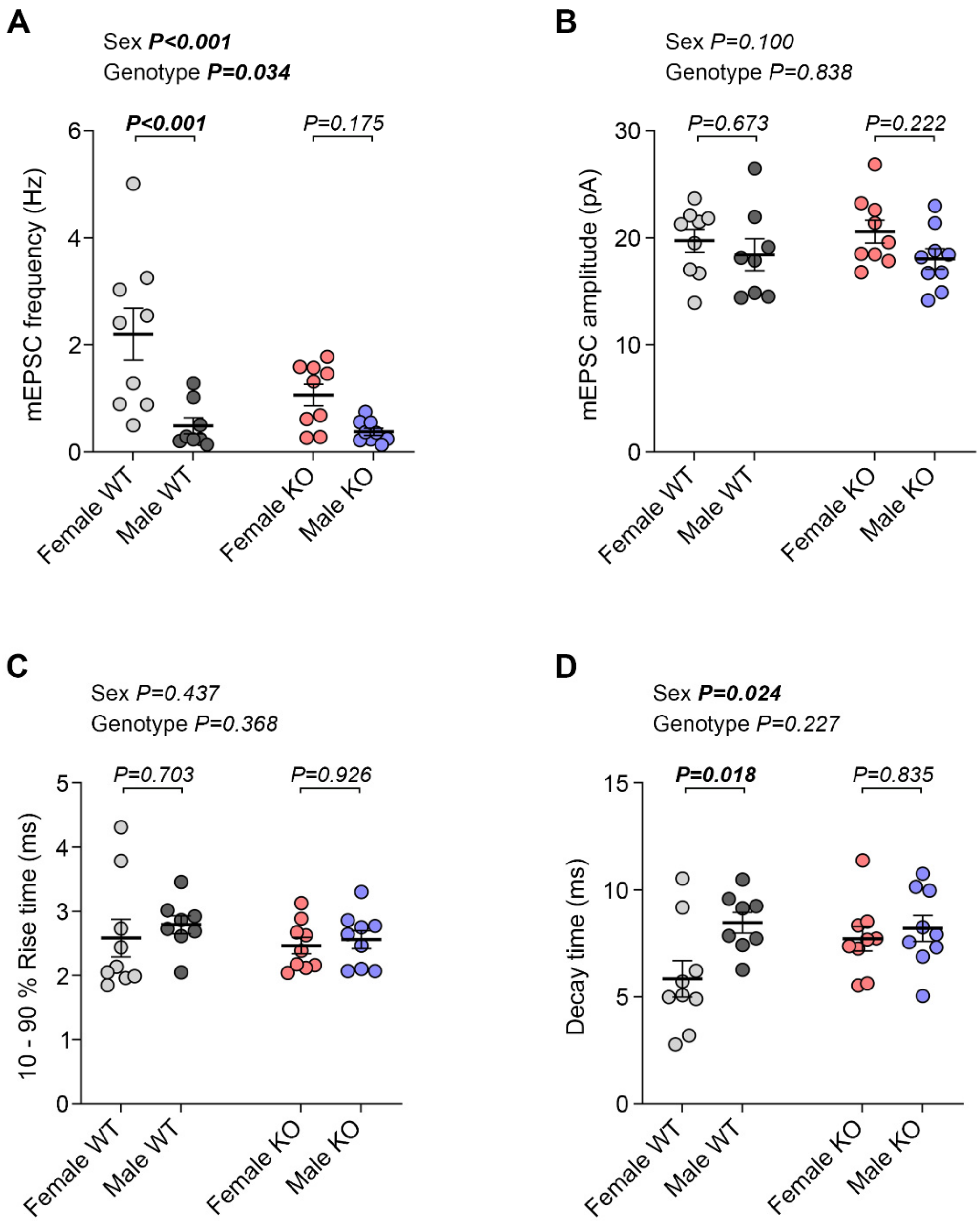


## Supplementary Figure 18: Intrinsic sex differences in mEPSC frequency and decay time in vCA1 pyramidal neurons are diminished by *Vstm2b* deletion

Comparison of mEPSC frequency (**A**), amplitude (**B**), 10-90 % rise time (**C**) and decay time (**D**) among *Vstm2b* WT female, *Vstm2b* WT male, *Vstm2b* KO female and *Vstm2b* KO male mice at P28. Statistical significance was determined by two-way ANOVA with Šídák's multiple comparisons post hoc test (α = 0.05). Data are shown as mean ± SEM, and each point represents one recorded cell. Sample numbers: *n* = 9 cells from 6 female WT mice; *n* = 8 cells from 5 male WT mice; *n* = 9 cells from 6 female KO mice; *n* = 9 cells from 5 male KO mice.

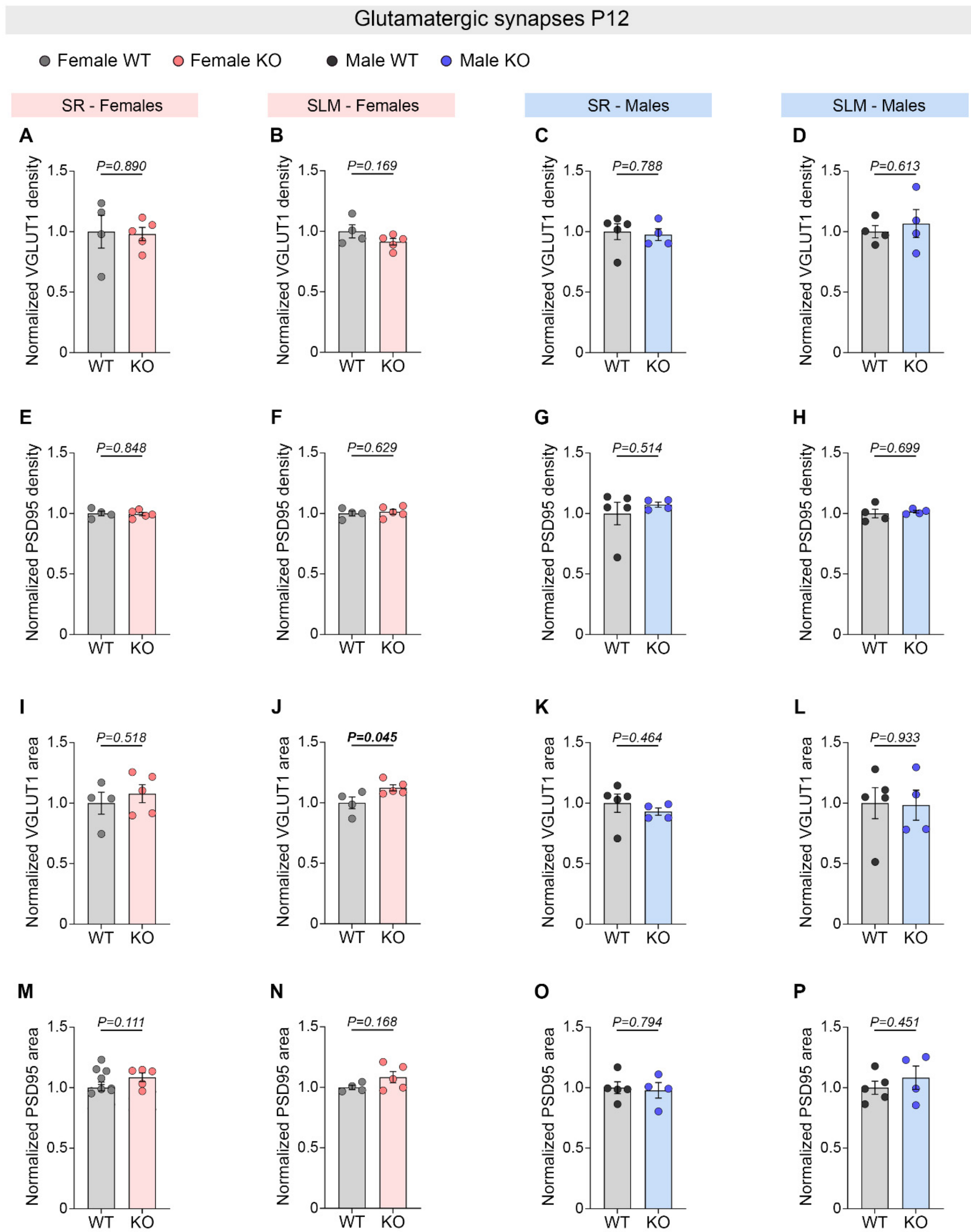


## Supplementary Figure 19: Quantification of VGLUT1 and PSD95 puncta in the ventral hippocampus of *Vstm2b* WT and KO mice at P12

**(A-H)** Quantification of the density of VGLUT1 (**A-D**) and PSD95 (**E-H**) puncta in the ventral hippocampal SR and SLM regions of *Vstm2b* WT and KO female and male mice at P12.
**(I-P)** Quantification of the area of VGLUT1 (**I-L**) and PSD95 (**M-P**) puncta in the ventral

hippocampal SR and SLM regions of *Vstm2b* WT and KO female and male mice at P12. Statistical significance was determined by unpaired two-tailed *t*-tests (α = 0.05). Data are shown as mean ± SEM, and each point represents the averaged result from one mouse; *n* = 4 female WT mice; *n* = 5 female KO mice; *n* = 5 (**C**, **G**, **K**, **L**, **O**, and **P**) and 4 (**D** and **H**) male WT mice; *n* = 4 male KO mice.

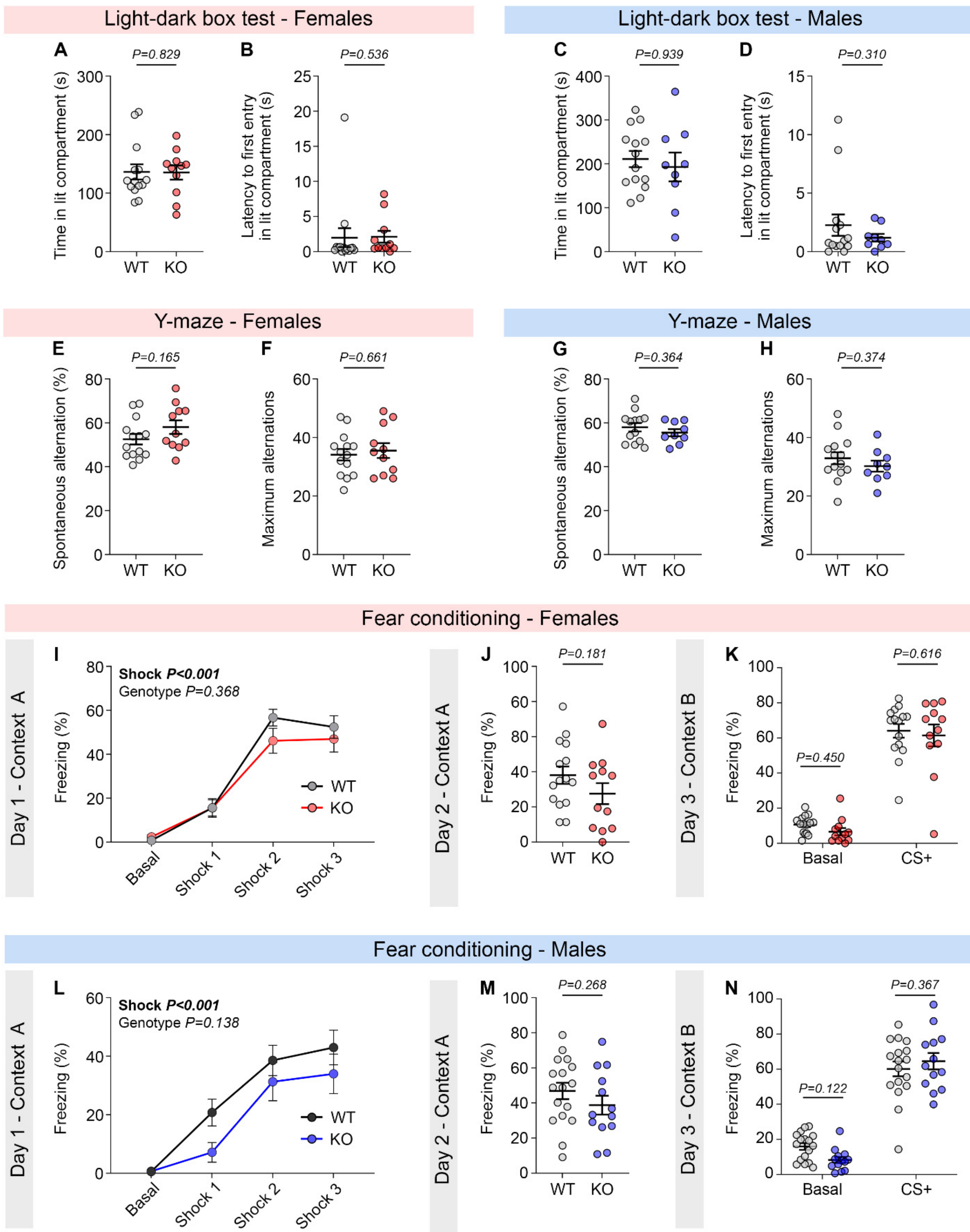


**Supplementary Figure 20: Behavioral phenotyping of *Vstm2b* KO mice**

**(A-D)** Light-dark test. Time spent in the lit compartment (**A, C**) and latency to first entry into

the lit compartment (**B**, **D**) of *Vstm2b* WT and KO littermates tested in the light-dark box paradigm. Statistical significance was examined with unpaired Mann-Whitney tests ($\alpha$ = 0.05).

**(E-H)** Y-maze. Percentage of spontaneous alternation (**E** and **G**) and number of maximum possible alternations of *Vstm2b* WT and KO littermates. Statistical significance was examined with unpaired two-tailed t-tests ($\alpha$ = 0.05).

**(I-N)** Fear conditioning test (CFC). Freezing behavior of *Vstm2b* WT and KO littermates during fear acquisition on day 1 (**I** and **L**), during fear contextual memory retrieval on day 2 (**J** and **M**) and during cued fear memory retrieval in an altered context on day 3 (**K** and **N**). Statistical significance was examined with two-way ANOVA with post hoc Fisher's least significant difference (LSD) (**I**, **L**, **K** and **N**) or unpaired two-tailed t-tests (**J** and **M**) ($\alpha$ = 0.05).

Sample number: n = 14 (LDB), 14 (Y-maze), 15 (CFC) mice for female WT; 11 (LDB), 11 (Y-maze), 12 (CFC) mice for female KO; n= 14 (LDB), 14 (Y-maze), 17 (CFC) mice for male WT and 9 (LDB), 9 (Y-maze), 13 (CFC) mice for male KO. Data are shown as mean ± SEM.

**Uncropped blot images**

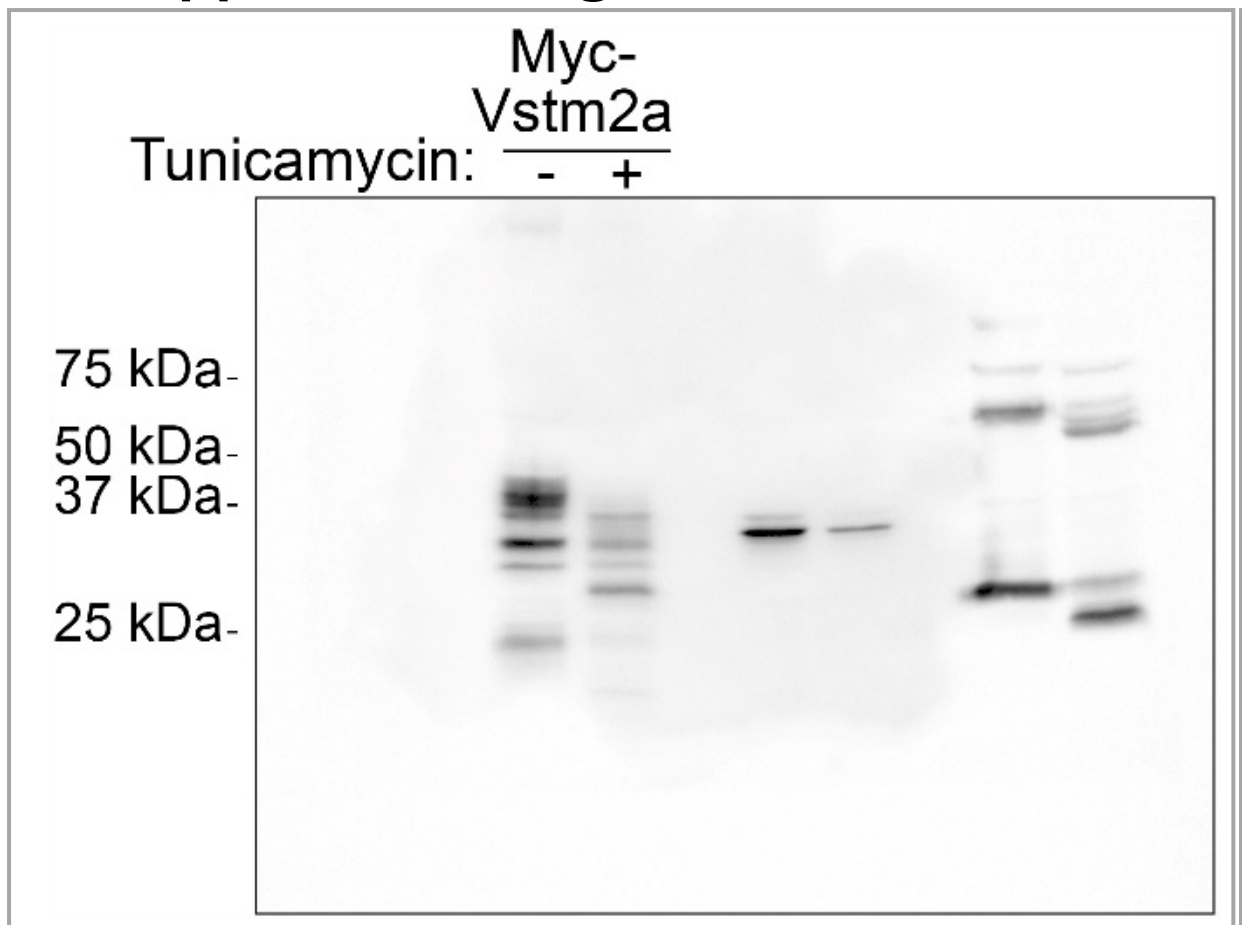


**Sup Fig. 6A_myc-Vstm2a**

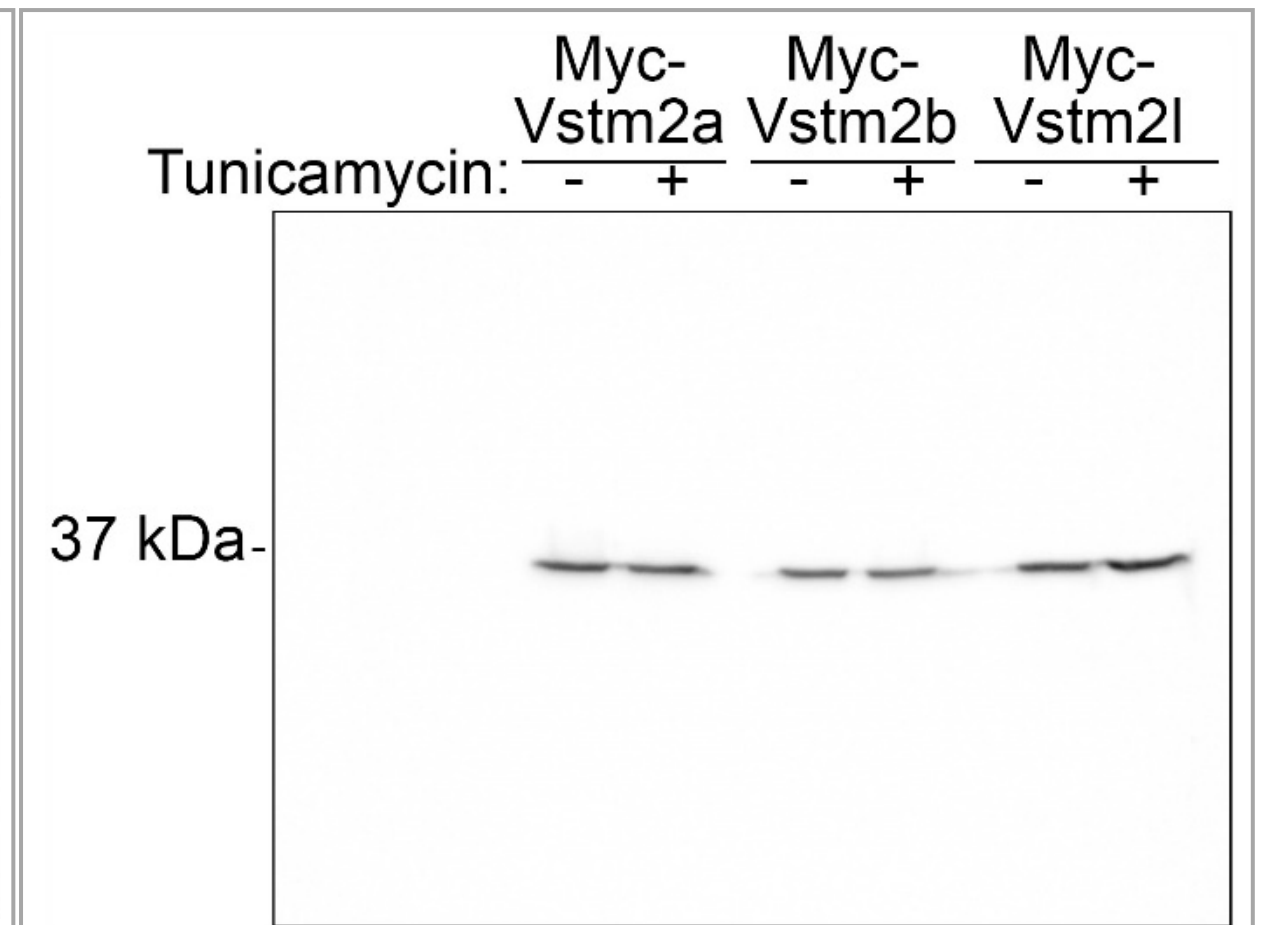


**Sup Fig. 6A_GAPDH**

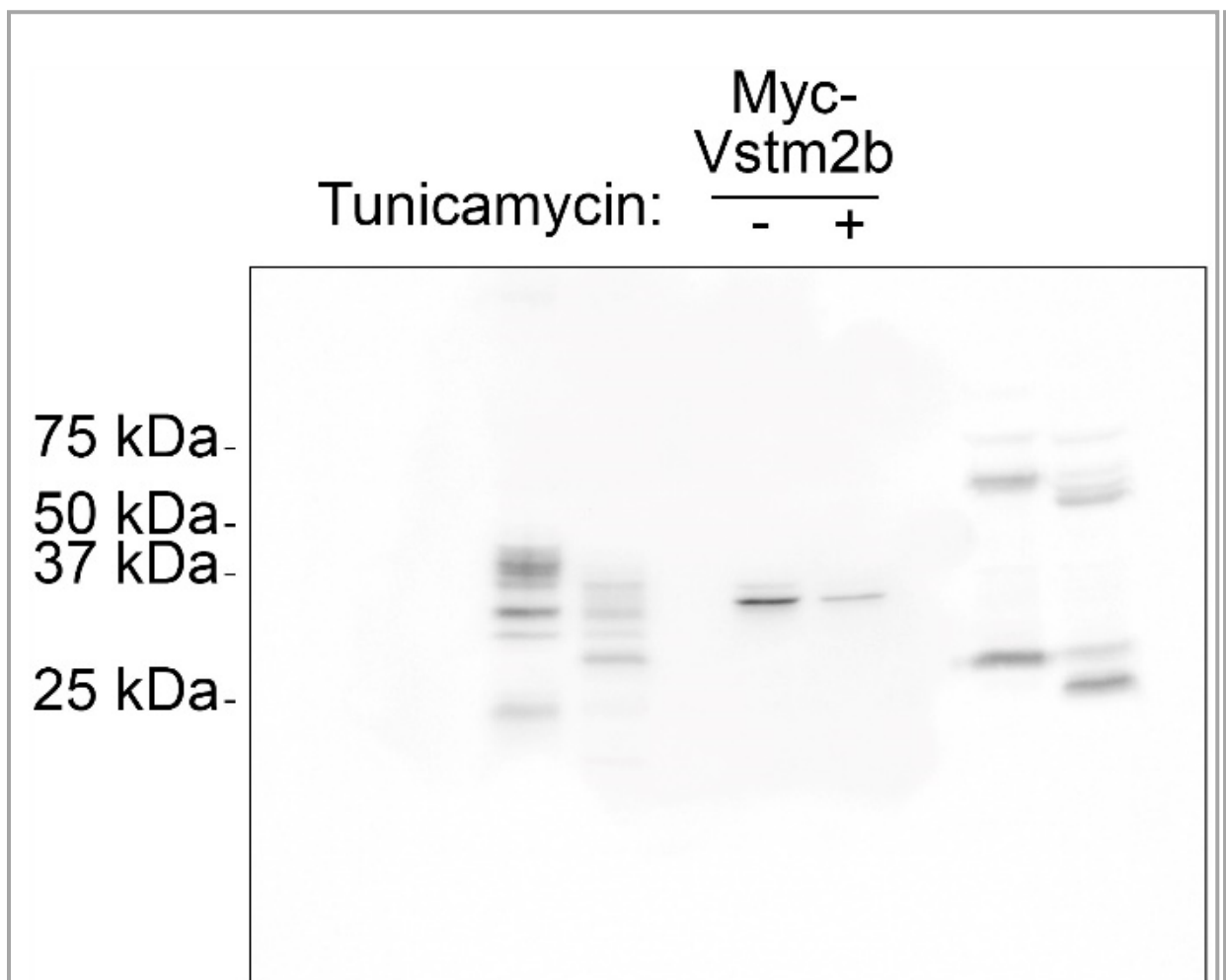


**Sup Fig. 6A_myc-Vstm2b**

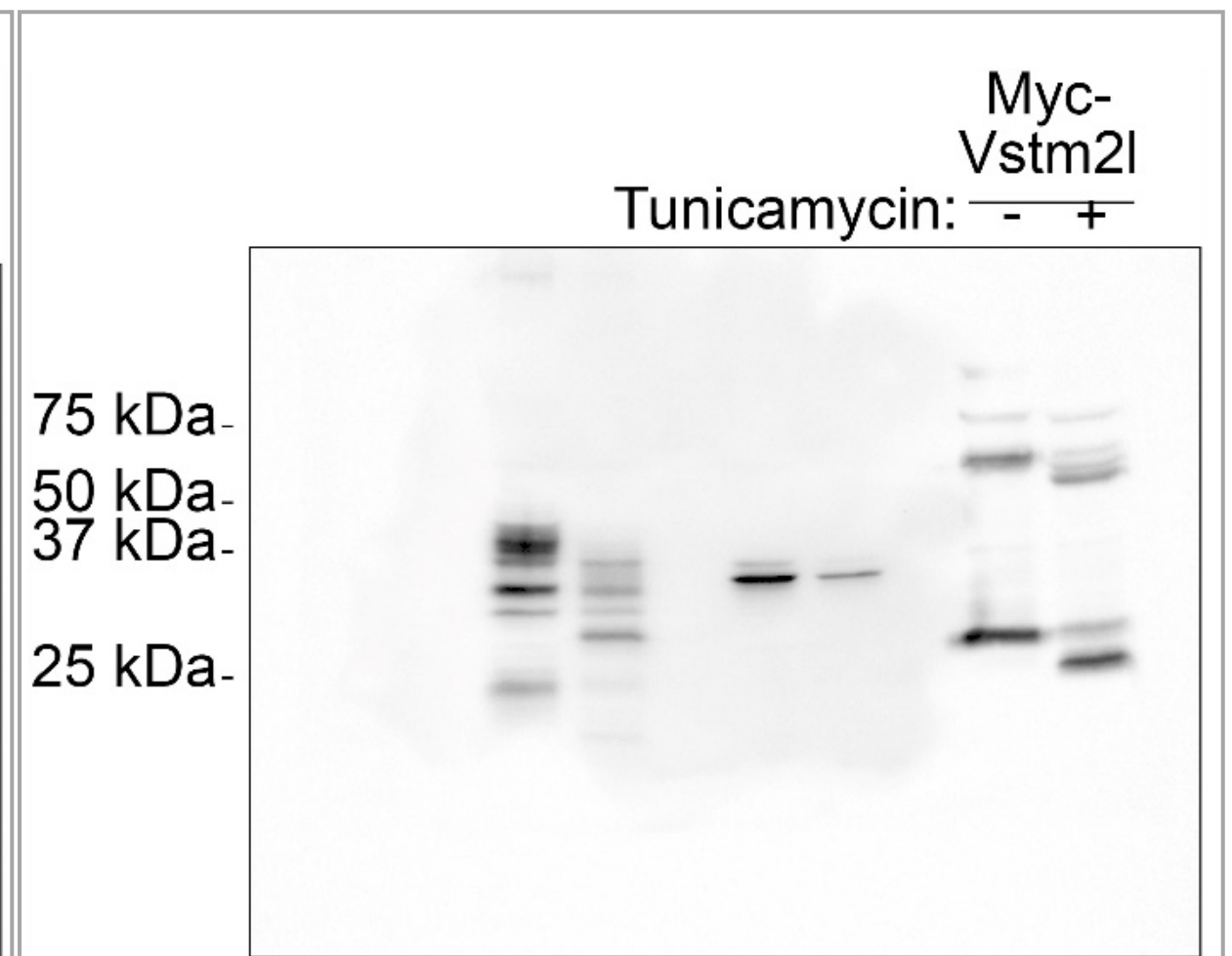


**Sup Fig. 6A_myc-Vstm2l**

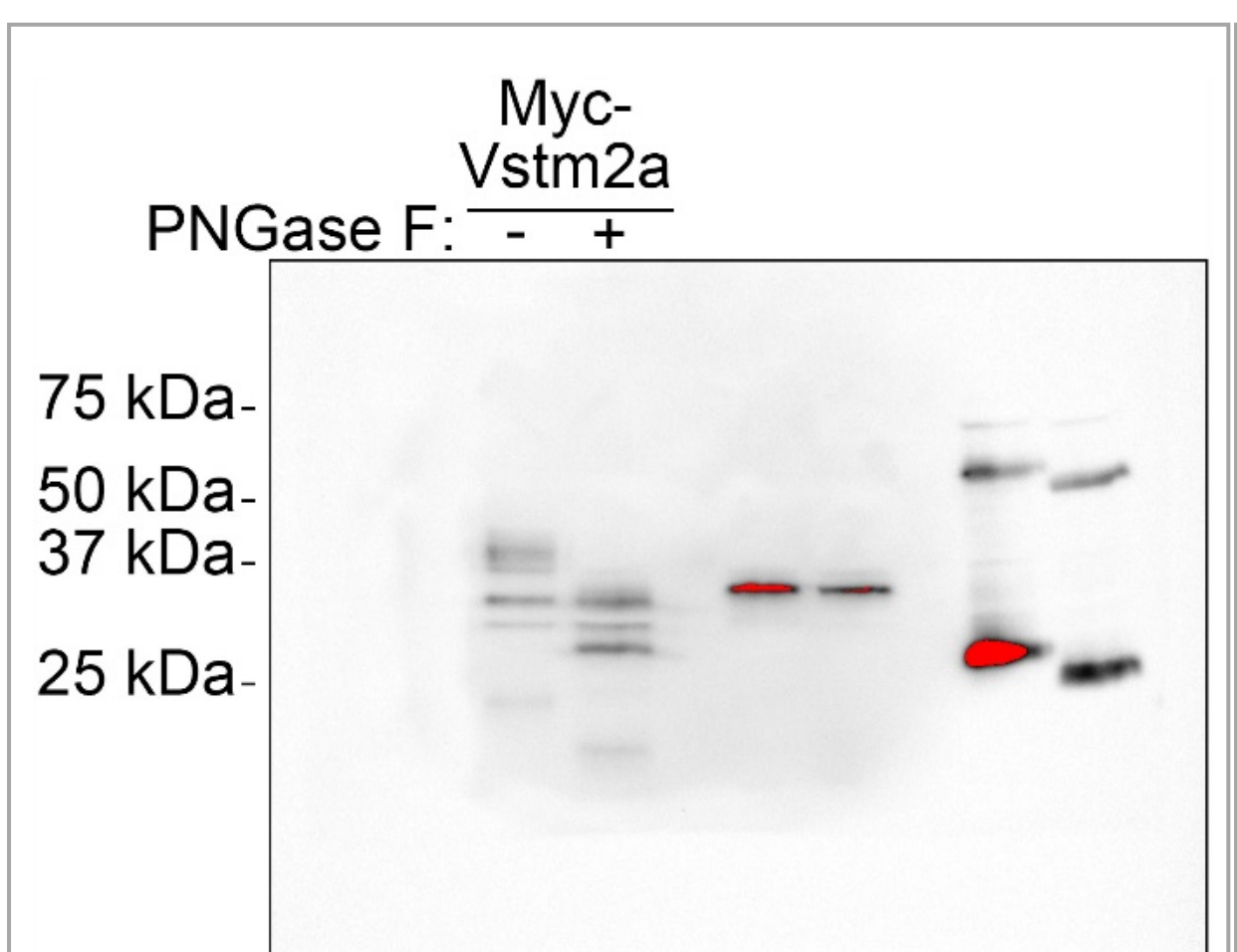


**Sup Fig. 6B_myc-Vstm2a**

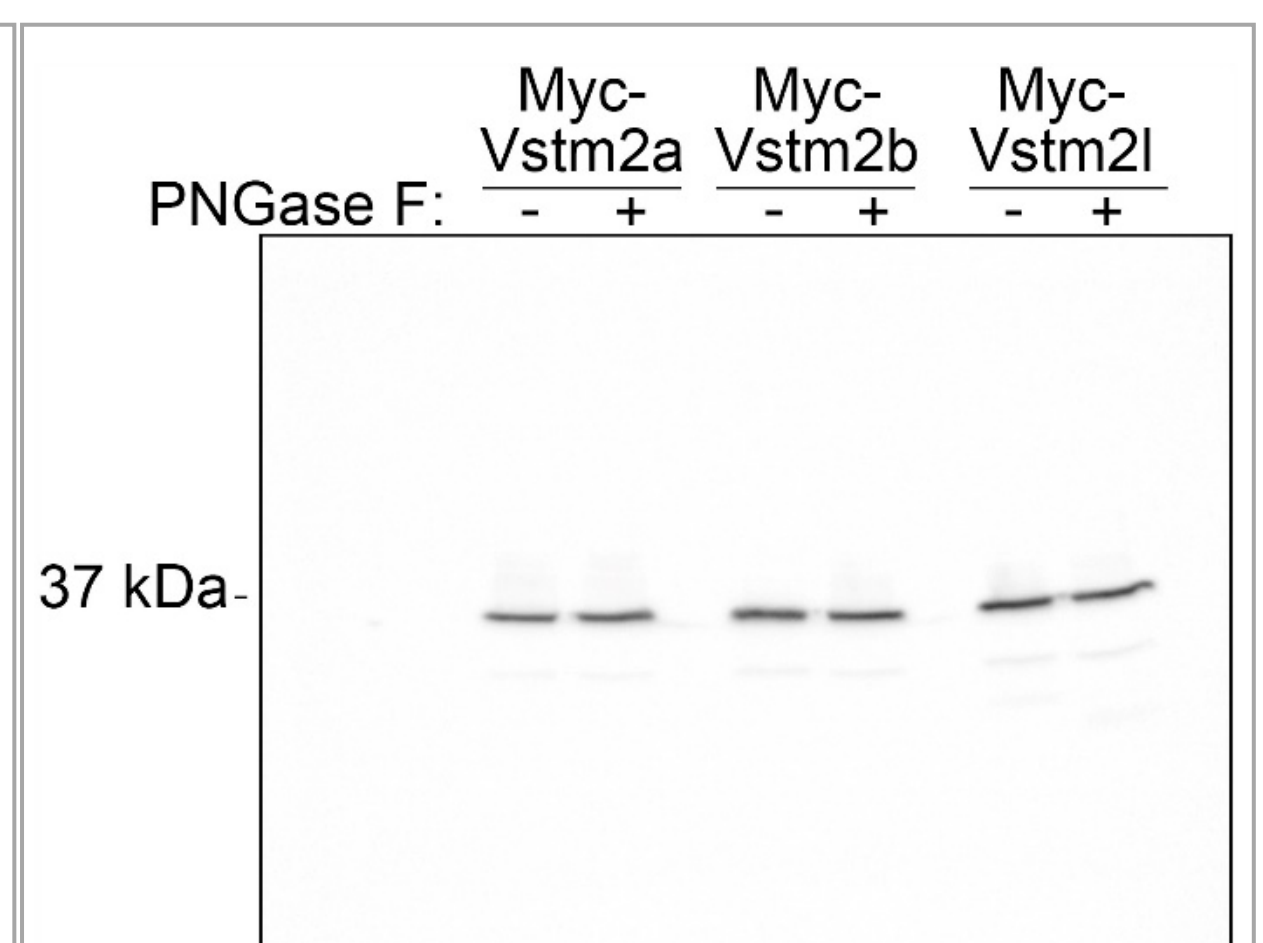


**Sup Fig. 6B_GAPDH**

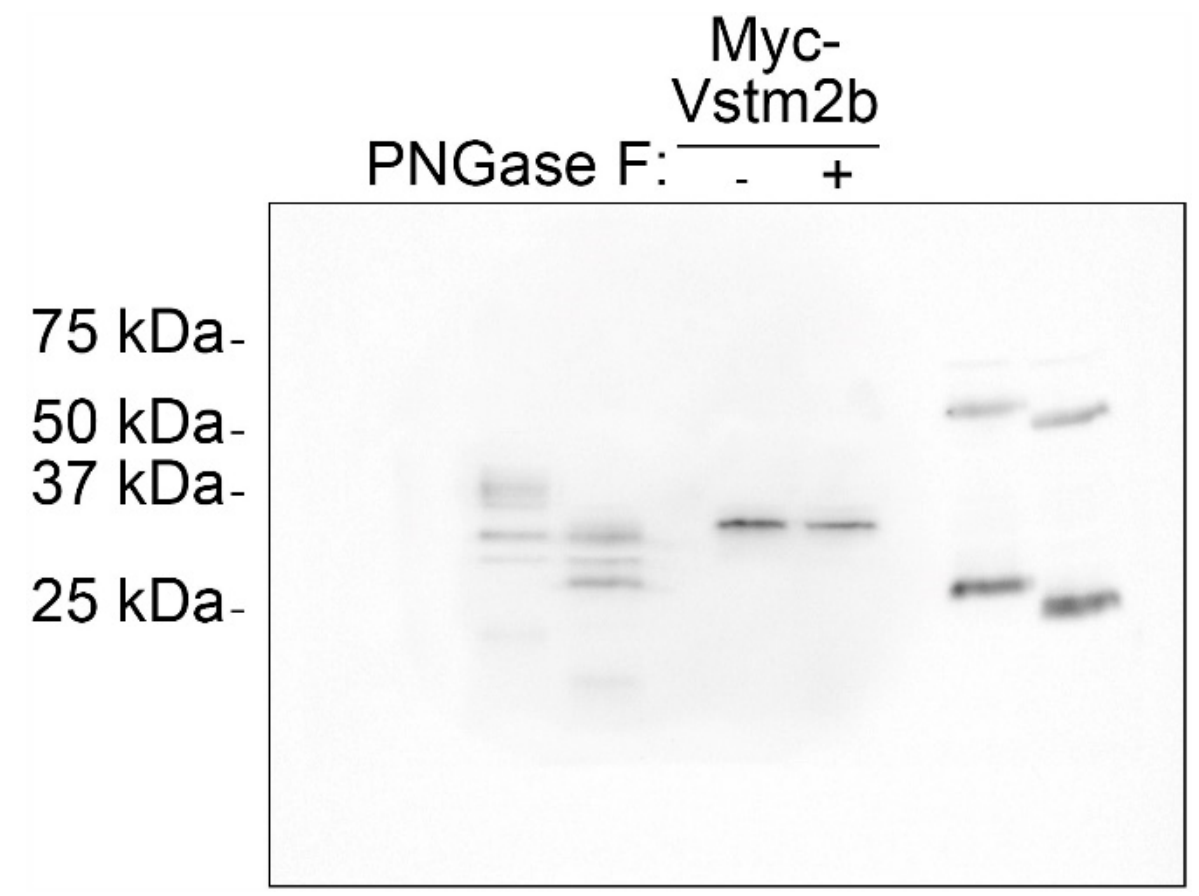


**Sup Fig. 6B_myc-Vstm2b**

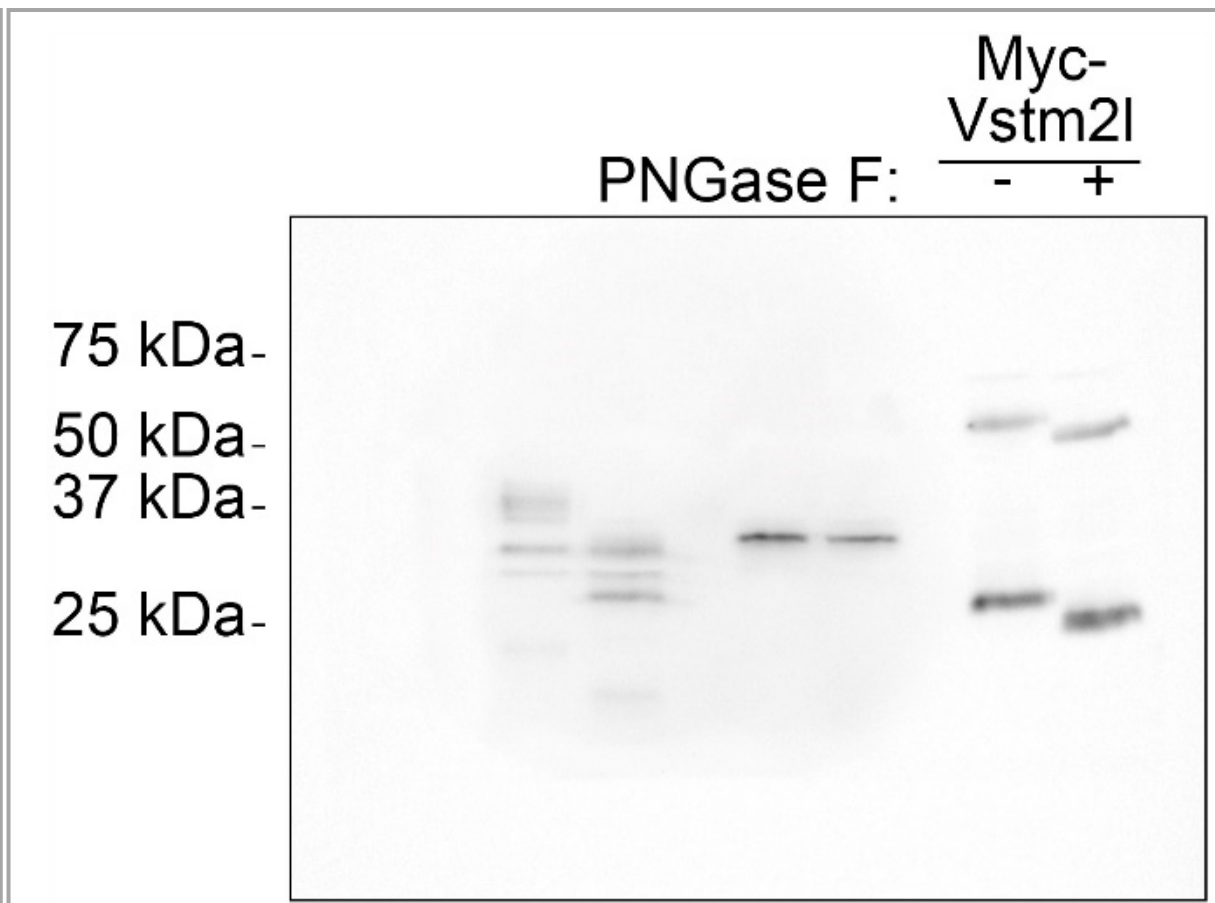


**Sup Fig. 6B_myc-Vstm2l**

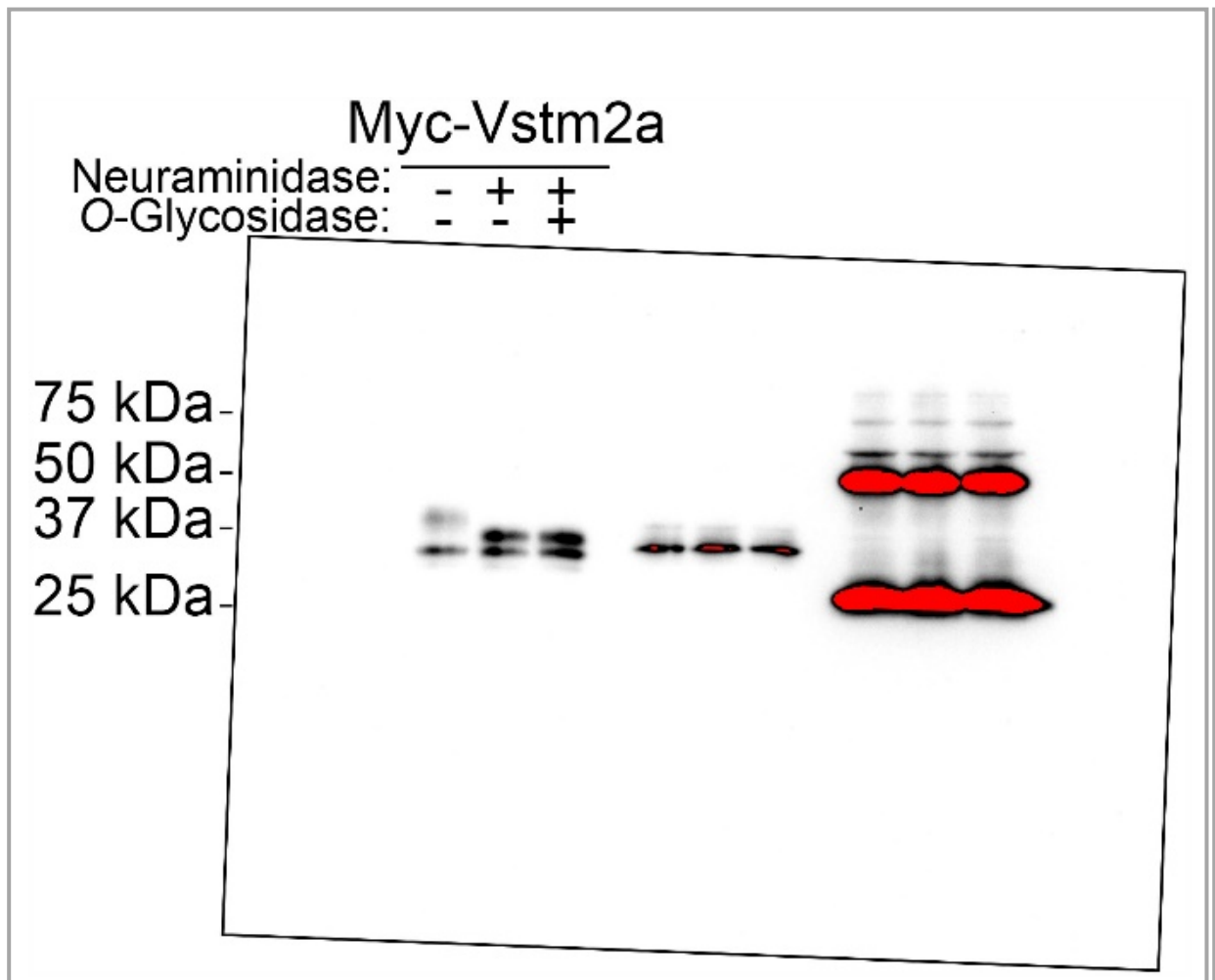


**Sup Fig. 6C_myc-Vstm2a**

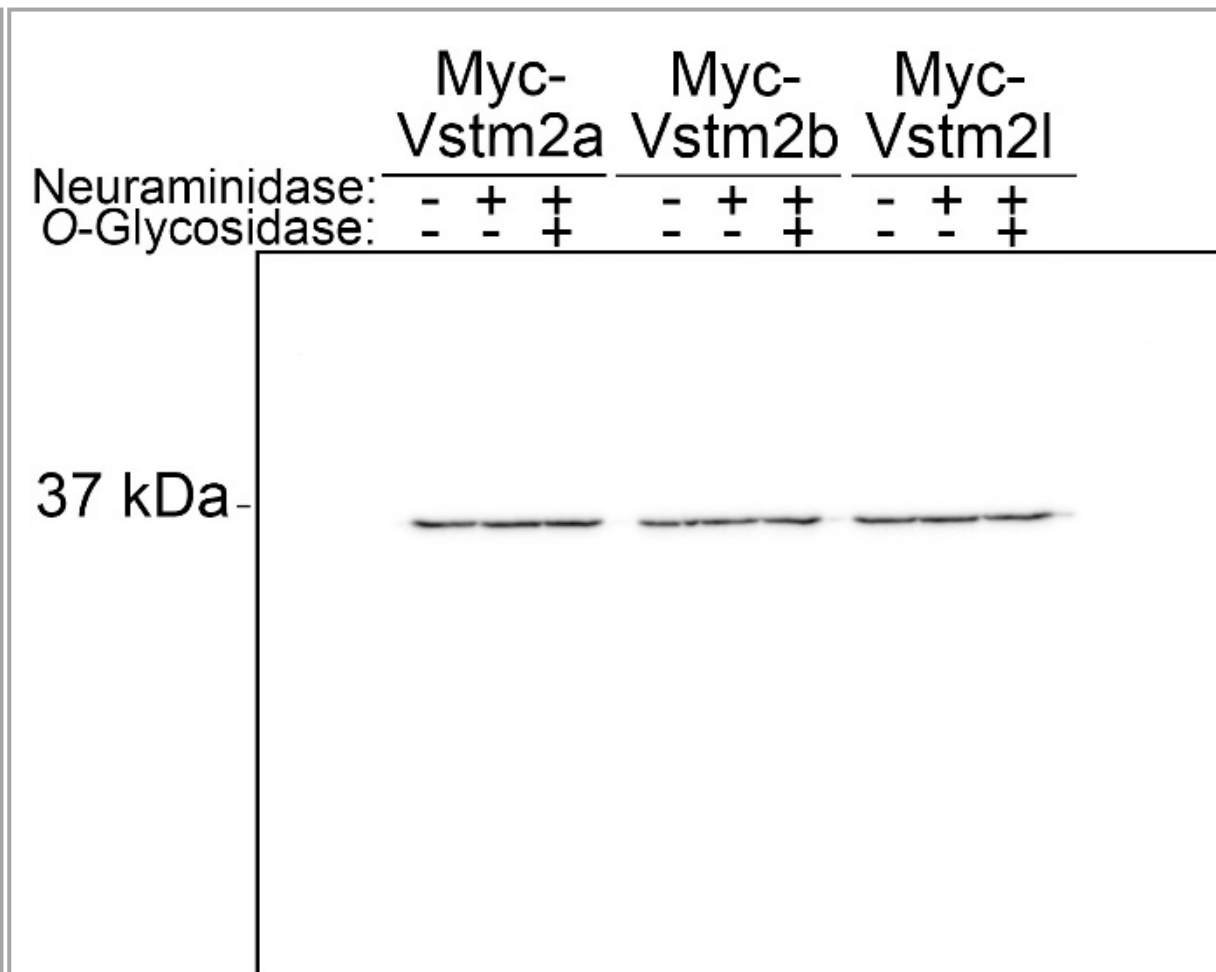


**Sup Fig. 6C_β-actin**

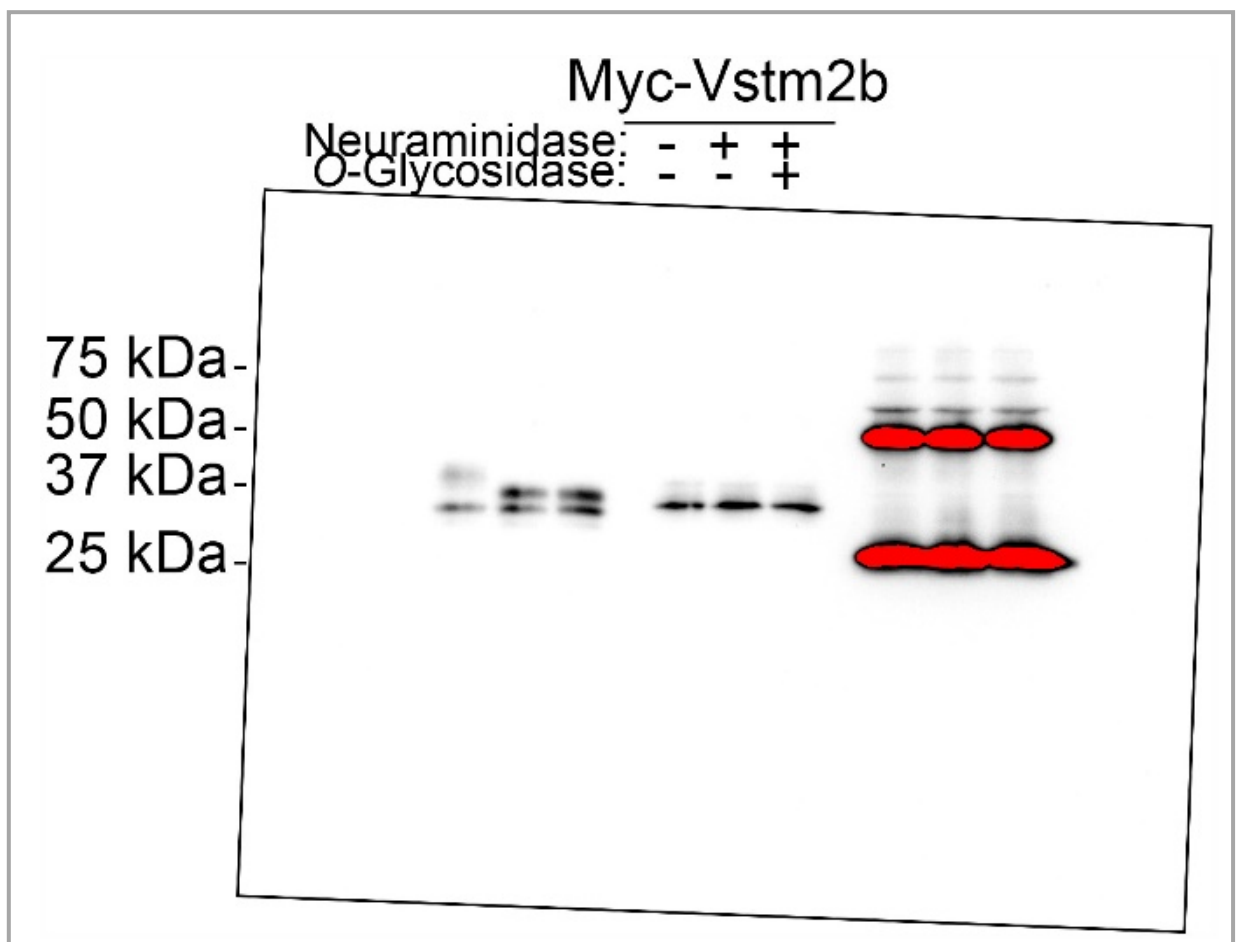


**Sup Fig. 6C_myc-Vstm2b**

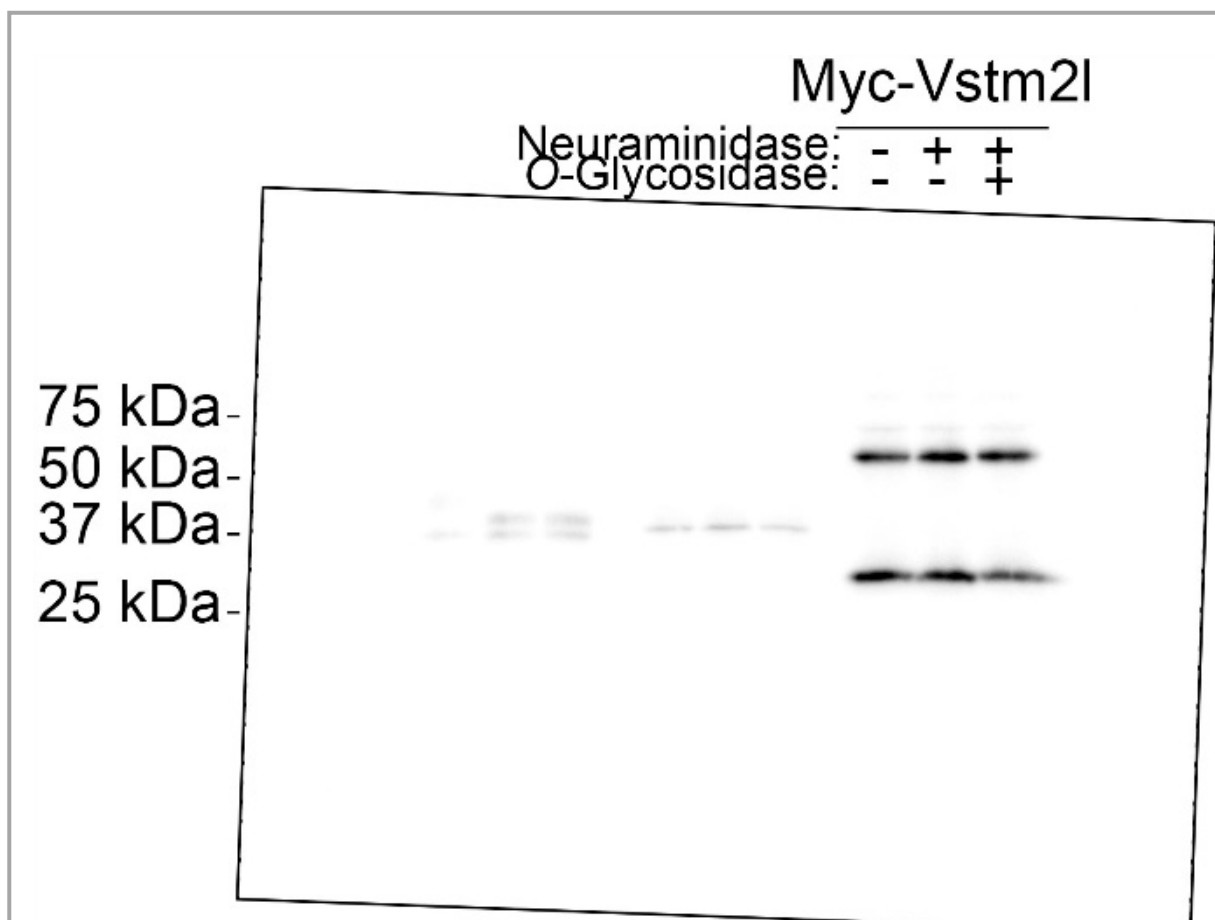


**Sup Fig. 6C_myc-Vstm2l**

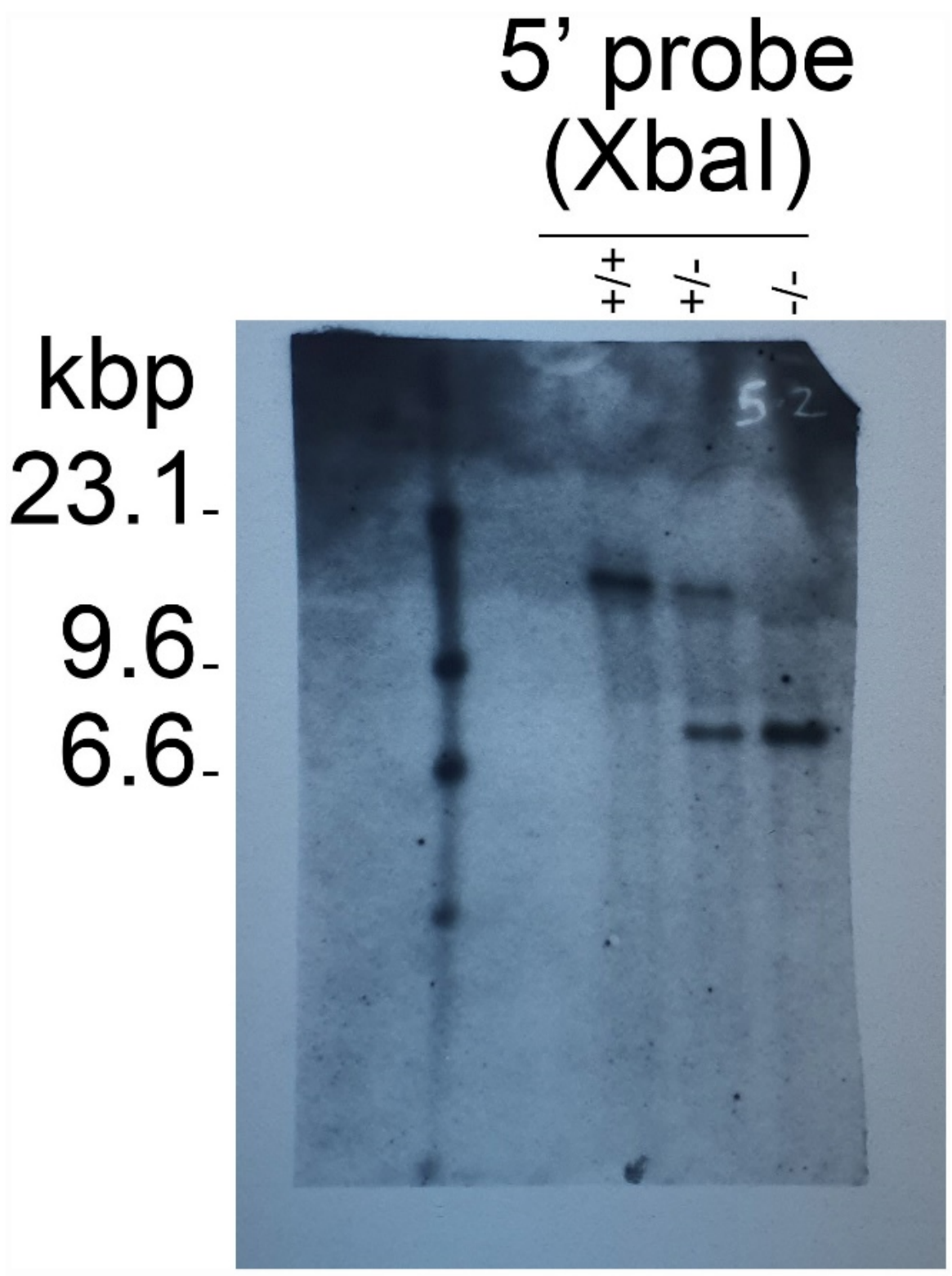


**Sup Fig. 7B_5’ probe (XbaI)**

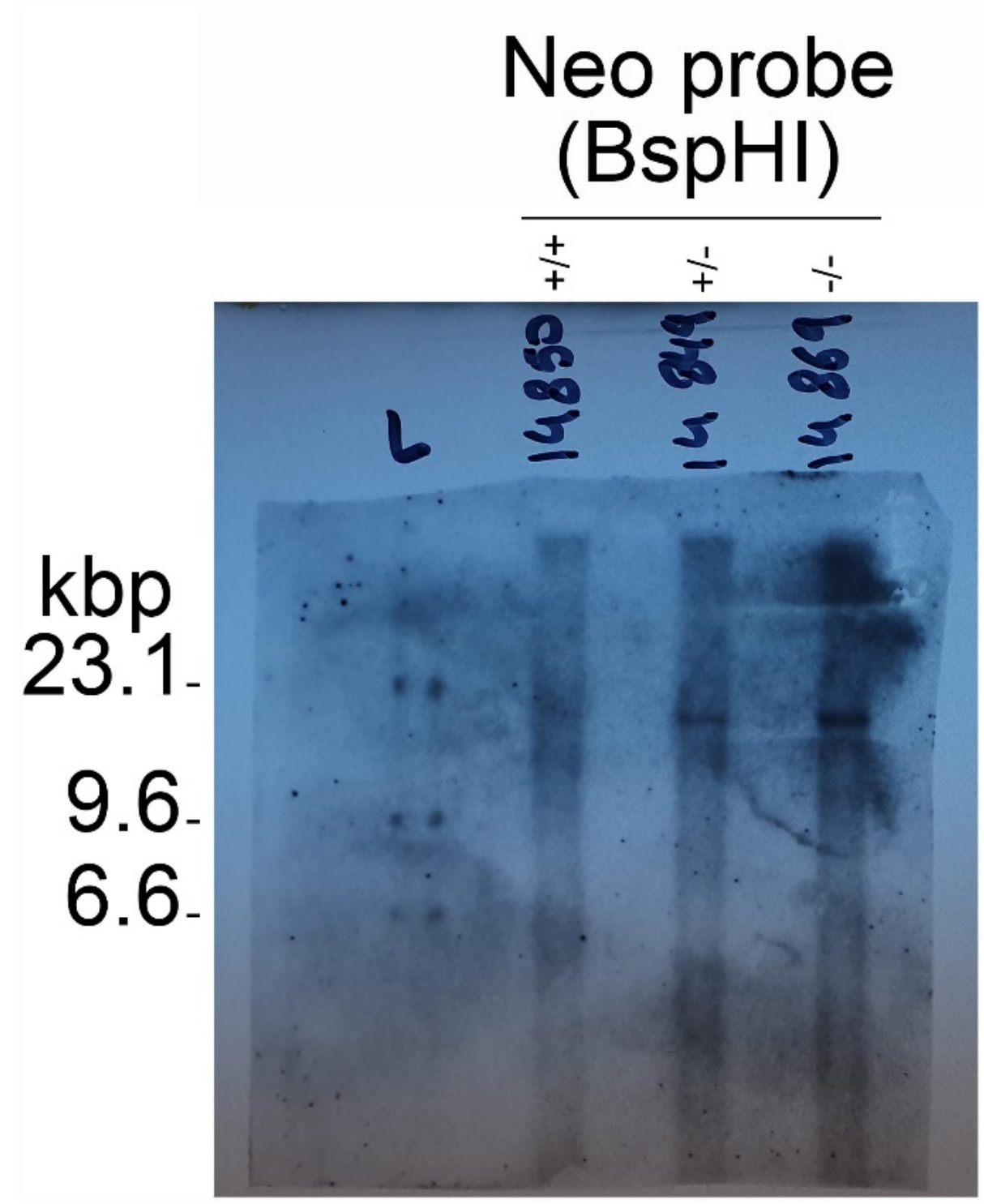


**Sup Fig. 7B_neo probe (BspHI)**

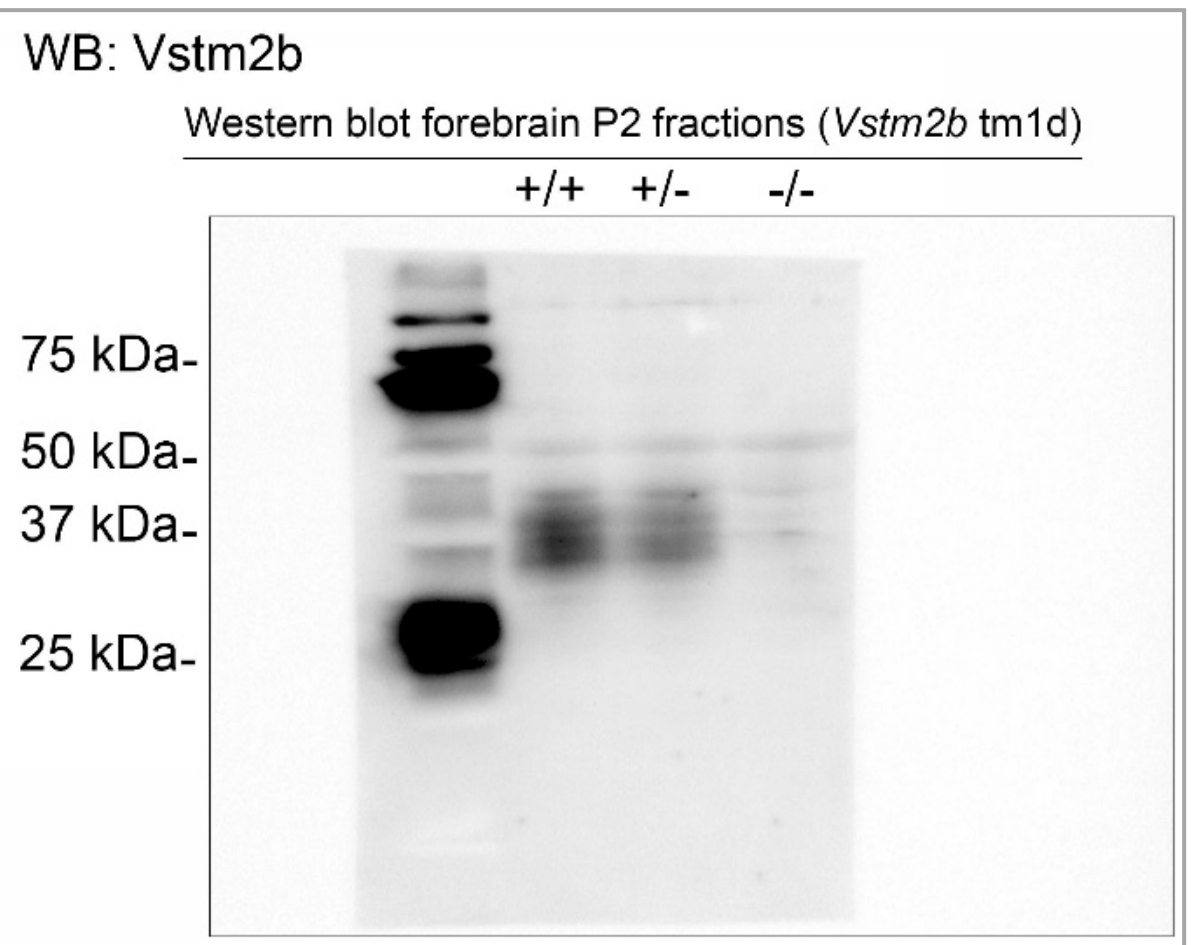


**Sup Fig. 7C_Vstm2b**

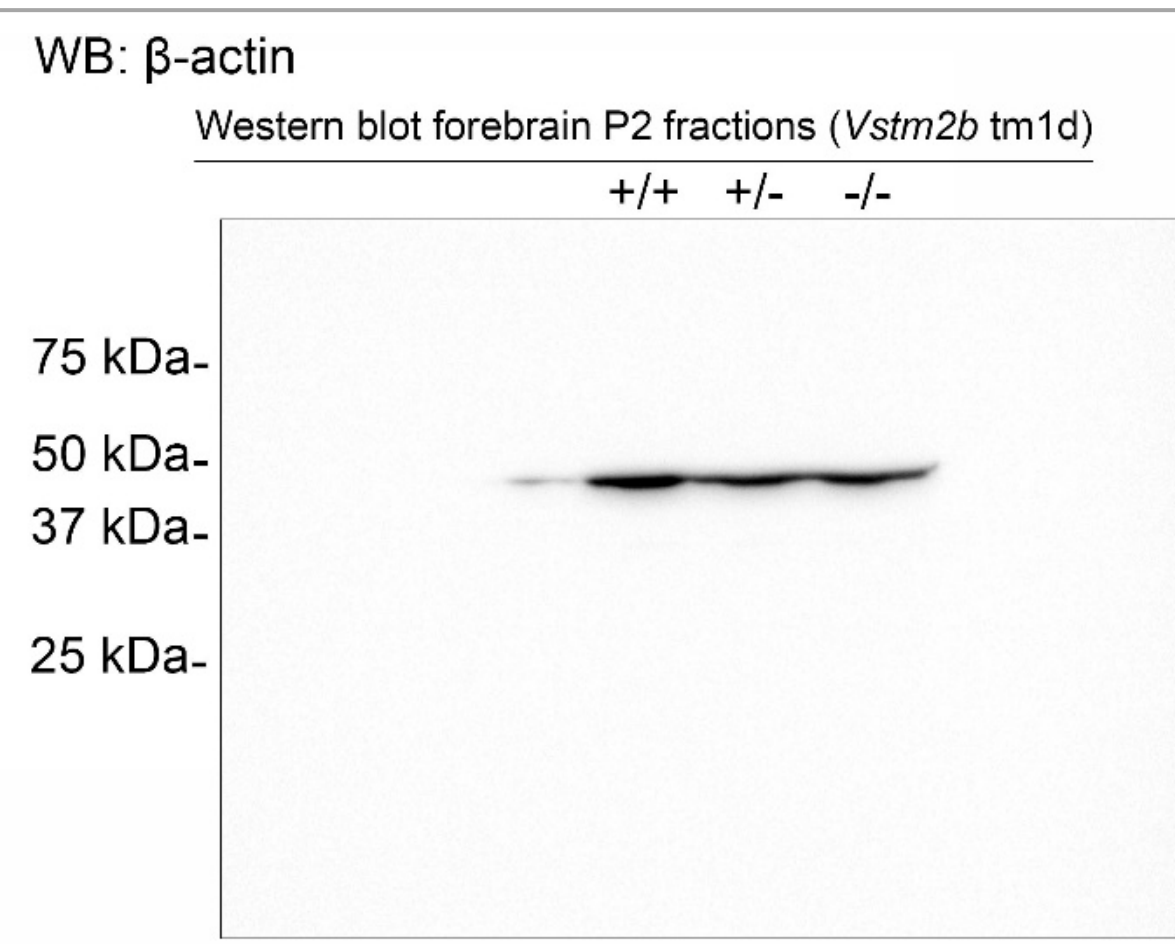


**Sup Fig. 7C_ β-actin**
