# Supplementary material for: Sexually dimorphic control of excitatory synapse maintenance in ventral hippocampal circuits by Neurexin2α ligand Vstm2b
Source: Nat Commun. 2026 Oct 5;17:11344. doi: 10.1038/s41467-026-78199-0 (PMC13728146; doi:10.1038/s41467-026-78199-0)
Supplement: Supplementary file 2 [file 41467_2026_78199_MOESM2_ESM.pdf]

nature portfolio

Corresponding author(s): Hideto Takahashi

Last updated by author(s): Aug 16, 2026

Reporting Summary

Nature Portfolio wishes to improve the reproducibility of the work that we publish. This form provides structure for consistency and transparency in reporting. For further information on Nature Portfolio policies, see our Editorial Policies and the Editorial Policy Checklist.

Statistics

For all statistical analyses, confirm that the following items are present in the figure legend, table legend, main text, or Methods section.

n/a	Confirmed	
☐	☒	The exact sample size (*n*) for each experimental group/condition, given as a discrete number and unit of measurement
☐	☒	A statement on whether measurements were taken from distinct samples or whether the same sample was measured repeatedly
☐	☒	The statistical test(s) used AND whether they are one- or two-sided<br>*Only common tests should be described solely by name; describe more complex techniques in the Methods section.*
☒	☐	A description of all covariates tested
☐	☒	A description of any assumptions or corrections, such as tests of normality and adjustment for multiple comparisons
☐	☒	A full description of the statistical parameters including central tendency (e.g. means) or other basic estimates (e.g. regression coefficient) AND variation (e.g. standard deviation) or associated estimates of uncertainty (e.g. confidence intervals)
☐	☒	For null hypothesis testing, the test statistic (e.g. *F*, *t*, *r*) with confidence intervals, effect sizes, degrees of freedom and *P* value noted<br>*Give P values as exact values whenever suitable.*
☒	☐	For Bayesian analysis, information on the choice of priors and Markov chain Monte Carlo settings
☒	☐	For hierarchical and complex designs, identification of the appropriate level for tests and full reporting of outcomes
☒	☐	Estimates of effect sizes (e.g. Cohen's *d*, Pearson's *r*), indicating how they were calculated

Our web collection on statistics for biologists contains articles on many of the points above.

Software and code

Policy information about availability of computer code

Data collection	Microscopy: ZEN Pro v3.10 (Zeiss); LAS X v3.7.4.23463 (Leica Microsystems)<br>Western blotting: Image Lab v6.0 (Bio-Rad)<br>RT-qPCR: QuantStudio™ 5 Real-Time PCR System software (Applied Biosystems)<br>Electrophysiology: Clampex v11.2 (Molecular Devices)<br>Behavioral analysis: EthoVision XT v17.5 (Noldus Information Technology)
Data analysis	Microscopy: Volocity v6.0 (PerkinElmer); ImageJ v1.54 (NIH); SynBot (PMID: 39255792); QuPath v0.4.3; Metamorph 7.8<br>Protein structure prediction: ColabFold v1.5.5 (a modified implementation of AlphaFold2)<br>Mass spectrometry: PeptideProphet v7.3.0<br>Statistical analysis: GraphPad Prism v10.2.0<br>Electrophysiology: Clampfit v11.2 (Molecular Devices)<br>Behavioral analysis: EthoVision XT v17.5 (Noldus Information Technology)<br>RNA-seq analysis: featureCounts v2.0.6

For manuscripts utilizing custom algorithms or software that are central to the research but not yet described in published literature, software must be made available to editors and reviewers. We strongly encourage code deposition in a community repository (e.g. GitHub). See the Nature Portfolio guidelines for submitting code & software for further information.

Data

Policy information about availability of data

All manuscripts must include a data availability statement. This statement should provide the following information, where applicable:

- Accession codes, unique identifiers, or web links for publicly available datasets
- A description of any restrictions on data availability
- For clinical datasets or third party data, please ensure that the statement adheres to our policy

All data supporting the findings of this study are available within the Article and its Supplementary Information. Source data are also provided with this paper. The mass spectrometry raw data have been deposited in the ProteomeXchange Consortium via the jPOST partner repository with the dataset identifiers PXD079897 for Fig. 1A-D, PXD079898 for Fig. 4E and PXD079896 for Supplementary Fig. 12. Some results from the following publicly available dataset were analyzed: GEO:GSE133291.

Research involving human participants, their data, or biological material

Policy information about studies with human participants or human data. See also policy information about sex, gender (identity/presentation), and sexual orientation and race, ethnicity and racism.

Reporting on sex and gender	The study did not involve human participants.
Reporting on race, ethnicity, or other socially relevant groupings	The study did not involve human participants.
Population characteristics	The study did not involve human participants.
Recruitment	The study did not involve human participants.
Ethics oversight	The study did not involve human participants.

Note that full information on the approval of the study protocol must also be provided in the manuscript.

Field-specific reporting

Please select the one below that is the best fit for your research. If you are not sure, read the appropriate sections before making your selection.

☒ Life sciences ☐ Behavioural & social sciences ☐ Ecological, evolutionary & environmental sciences

For a reference copy of the document with all sections, see nature.com/documents/nr-reporting-summary-flat.pdf

Life sciences study design

All studies must disclose on these points even when the disclosure is negative.

Sample size	Sample sizes were chosen based on previous studies employing similar experimental designs.
Data exclusions	No data were excluded in this study.
Replication	All data were obtained from multiple independent experiments, with successful replication in all cases.
Randomization	Samples were allocated to experimental groups according to predefined biological variables relevant to each experiment. For in vitro studies, cells were assigned to groups based on transfection condition (different plasmids). For in vivo experiments, animals were grouped according to sex and genotype as defined by the study design. Randomization was not applicable. All samples were processed and analyzed using identical protocols to minimize technical and batch-related variability.
Blinding	Investigators were blinded to experimental conditions during microscopy-based , electrophysiological and behavioral data collection and analysis.

Reporting for specific materials, systems and methods

We require information from authors about some types of materials, experimental systems and methods used in many studies. Here, indicate whether each material, system or method listed is relevant to your study. If you are not sure if a list item applies to your research, read the appropriate section before selecting a response.

Materials & experimental systems

n/a	Involved in the study
	☒ Antibodies
	☒ Eukaryotic cell lines
☒	Palaeontology and archaeology
	☒ Animals and other organisms
☒	Clinical data
☒	Dual use research of concern
☒	Plants

Methods

n/a	Involved in the study
☒	ChIP-seq
☒	Flow cytometry
☒	MRI-based neuroimaging

Antibodies

Antibodies used

anti-HA (rat IgG1, clone 3F10, Roche, Cat#: ROAHAHA)
anti-VGAT (polyclonal rabbit, Synaptic Systems, Cat#: 131003)
anti-VGLUT1 (polyclonal guinea pig, Synaptic Systems, Cat#: 135 304)
anti-HA (polyclonal rabbit, Abcam, Cat#: ab9110)
anti-myc (monoclonal mouse, clone 9E10, Santa Cruz, Cat#: sc-40)
anti-6xHis (polyclonal rabbit, Abcam, Cat#: ab213204)
anti-β-actin (polyclonal rabbit, Abcam, Cat#: ab8227)
anti-GAPDH (mouse IgG1, clone 0411, Santa Cruz Biotechnology, Cat: sc-47724)
anti-PSD95 (mouse IgG2a, clone 6G6-1C9, ThermoFischer Scientific, Cat#: MA1-045)
anti-GPHN (mouse IgG1, clone 3B11, Synaptic Systems, Cat#: 147 111)
anti-SYP (mouse IgG1, clone SY38, Millipore, Cat#: MAB5258)
anti-VGLUT1 (polyclonal rabbit, Synaptic Systems, Cat#: 135 308)
anti-β-gal (mouse IgG2a, Promega, Cat#: Z3781)
anti- parvalbumin (PVALB) (polyclonal guinea pig, Swant, Cat#: GP72)
anti- calretinin (CR) (polyclonal rabbit, Millipore, Cat#: ABN2191)
anti-Vstm2b (polyclonal guinea pig, custom made)

Validation

Anti HA (polyclonal rat, Roche, Cat#: ROAHAHA)
• Applications: WB, IP, ICC/IF, ELISA (vendor-recommended)
• RRID: AB_390918
Anti VGAT (polyclonal rabbit, Synaptic Systems, Cat#: 131003)
• Applications: WB, IP, ICC/IF, IHC, EM (vendor-recommended)
• RRID: AB_887869
Anti VGLUT1 (polyclonal guinea pig, Synaptic Systems, Cat#: 135304)
• Applications: WB, IP, ICC/IF, IHC (vendor-recommended)
• RRID: AB_887878
Anti HA (polyclonal rabbit, Abcam, Cat#: ab9110)
• Applications: WB, IP, ICC/IF, ChIP (vendor-recommended)
• RRID: AB_307019
Anti myc (monoclonal mouse, clone 9E10, Santa Cruz, Cat#: sc 40)
• Applications: WB, IP, ICC (vendor-recommended)
Anti 6xHis (polyclonal rabbit, Abcam, Cat#: ab213204)
• Applications: WB, IP, ELISA (vendor-recommended)
• RRID: AB_2893578
Anti β actin (polyclonal rabbit, Abcam, Cat#: ab8227)
• Applications: WB (vendor-recommended)
• RRID: AB_306371
Anti GAPDH (mouse IgG1, clone 0411, Santa Cruz Biotechnology, Cat: sc 47724)
• Applications: WB, ICC (vendor-recommended)
• RRID: AB_627678
Anti PSD95 (mouse IgG2a, clone 6G6 1C9, ThermoFisher Scientific, Cat#: MA1 045)
• Applications: WB, ICC/IF (vendor-recommended)
• RRID: AB_2251530
Anti GPHN (mouse IgG1, clone 3B11, Synaptic Systems, Cat#: 147111)
• Applications: ICC/IF, IHC, WB (vendor-recommended)
• RRID: AB_2621098
Anti SYP (mouse IgG1, clone SY38, Millipore, Cat#: MAB5258)
• Applications: WB, ICC/IF (vendor-recommended)
• RRID: AB_2286945
Anti VGLUT1 (polyclonal rabbit, Synaptic Systems, Cat#: 135308)
• Applications: WB, IP, ICC, IHC (vendor-recommended)
• RRID: AB_2893580
Anti β gal (monoclonal mouse; Promega, Cat#: Z3781)

• Applications: WB, ICC (vendor-recommended)
• RRID: AB_430877
Anti parvalbumin (polyclonal guinea pig, Swant, Cat#: GP72)
• Applications: IF, IHC (vendor-recommended)
• RRID: AB_10000343
Anti calretinin (polyclonal rabbit, Millipore, Cat#: ABN2191)
• Applications: ICC/IF, IHC (vendor-recommended)
• RRID: AB_10806799

Antibodies used for in vitro assays were validated by either Western blotting (WB) or immunocytochemistry (ICC) using non-targeting control (NTC) lysates or cells, respectively. For WB validation, the presence of bands at the expected molecular weights was monitored to confirm antibody specificity. For the analysis of synaptic markers in the mouse brain, antibodies against VGLUT1 and PSD95, both widely used and well-characterized in the neuroscience field, were employed for co-immunostaining. The expected enrichment patterns of immunofluorescence for these markers were confirmed across distinct hippocampal regions. Furthermore, the degree of colocalization between VGLUT1 and PSD95 signals was significantly reduced upon 90° rotation of one fluorescence channel, supporting physiologically relevant colocalization corresponding to pre- and postsynaptic compartments. The custom-made Vstm2b antibody was validated by Western blotting, where a band at the expected molecular weight was detected in control samples and was absent in homozygous knockout brain extracts, confirming antibody specificity.

Eukaryotic cell lines

Policy information about cell lines and Sex and Gender in Research

Cell line source(s)	COS-7 cells (ATCC CRL-1651) and 293T cells (ATCC CRL-3216) were used. Primary hippocampal and cortical neurons were prepared from mouse or rat embryos, regardless of sex.
Authentication	None of the cell lines used in this study were authenticated.
Mycoplasma contamination	None of the cell lines used in this study were tested for mycoplasma contamination.
Commonly misidentified lines (See ICLAC register)	None of the cell lines used in this study are known to be commonly misidentified.

Animals and other research organisms

Policy information about studies involving animals; ARRIVE guidelines recommended for reporting animal research, and Sex and Gender in Research

Laboratory animals	Adult pregnant Sprague Dawley rats (embryonic day 18, E18) and adult pregnant Nxrn2a KO and Vstm2b KO mice (E16-E18) were used to prepare primary neuron cultures. Vstm2b KO mice aged 2–4 months were used for behavioral analyses. Postnatal day 12 (P12) and day 28 (P28) C57BL/6J mice were used for biochemical and immunofluorescence analyses.
Wild animals	The study did not involve wild animals.
Reporting on sex	All experiments involving mice included sex as a biological variable. Sex was determined by visual inspection of external genitalia. Results are generally reported separately for males and females. Overall sample sizes for each sex are provided where applicable.
Field-collected samples	The study did not involve field-collected samples.
Ethics oversight	All animal procedures were conducted in accordance with the guidelines of the Canadian Council on Animal Care and were approved by the IRCM Animal Care Committee (Protocol number: 2020-10 and 2024-06).

Note that full information on the approval of the study protocol must also be provided in the manuscript.

Plants

Seed stocks	The study did not involve plants.
Novel plant genotypes	The study did not involve plants.
Authentication	The study did not involve plants.
