# Supplementary material for: Sexually dimorphic control of excitatory synapse maintenance in ventral hippocampal circuits by Neurexin2α ligand Vstm2b
Source: Nat Commun. 2026 Oct 5;17:11344. doi: 10.1038/s41467-026-78199-0 (PMC13728146; doi:10.1038/s41467-026-78199-0)
Supplement: Supplementary file 3 [file 41467_2026_78199_MOESM3_ESM.pdf]

nature portfolio

Peer Review File

Sexually Dimorphic Control of Excitatory Synapse Maintenance in Ventral Hippocampal Circuits by Neurexin2α Ligand Vstm2b

Corresponding Author: Professor Hideto Takahashi

This file contains all reviewer reports in order by version, followed by all author rebuttals in order by version.

Version 0:

Reviewer comments:

Reviewer #1

(Remarks to the Author)
In this manuscript by Chofflet and colleagues, the authors identify and characterize Vstm2b, a novel extracellular ligand for neurexin2a. Using a diverse set of biochemical, molecular, cellular, computational, electrophysiological, and behavioral assays, the authors convincingly identify Vstm2b as a ligand for neurexin2a. Using an unbiased screen, the authors identified Vstm2l as a putative Nrxn2 interacting protein. Vstm2l is a member of the Vstm family, which also includes Vstm2a and Vstm2b. Vstm2a and Vstm2b are expressed in the brain and are presented to the extracellular space via a GPI-anchor. However, Vstm2l is present on the membrane, yet robustly enriched with the Nrxn2a bait. The authors primarily focus on the properties of Vstm2b due to its specific interaction with Nrxn2a - it selectively binds LNS2, and is dependent on the inclusion of the SS2 alternative exon. When expressed in non-neuronal cells, Vstm2b robustly induces excitatory and inhibitory presynaptic assembly in cultured neurons. Further, the assembly of excitatory presynaptic terminals in this assay is dependent on presynaptic Nrxn2a. In neurons, Vstm2b regulates the number of synapses in ventral CA1 in a sex-dependent manner. This synapse number phenotype is not due the impaired synaptogenesis, but synapse maintenance. Functionally, global ablation of Vstm2b led to reduced basal excitatory transmission at Schaffer-collateral synapses in females but not males. Importantly, in young animals, the deletion of Vstm2b did not alter synapse numbers, but impaired basal synaptic transmission, which suggest that the synapse number phenotype in adult animals might be a consequence of impaired excitatory synapse function. Finally, using different experimental paradigms to study anxiety-like behavior, the authors report reduced anxiety-like behavior in female Vstm2b KO animals. Overall, this is well-done study that importantly expands our understanding of a-Nrxn-specific signaling at the synapse and provides additional insight into the role of Nrxn2a, arguably the least well characterized alpha-neurexin. The conclusions of the paper are supported by strong data and, as such, I only have minor comments.

1. Vstm2b is also expressed in GABAergic neurons (Suppl Fig. 6C) and recruits vGAT-positive terminals in the co-culture assay (Fig. 6). The authors should measure GABAergic markers in ventral CA1 of Vstm2b KO hippocampi with region-specificity (e.g. soma vs apical dendrite).
2. To supplement the experiments in Suppl Fig. G-L, can the authors use a Ca2+-binding deficient LNS2 mutant to further demonstrate that the Vstm2b-Nrxn2a interaction is Ca2+ independent?
3. Can the authors speculate why the LNS2SS2A+ only partially binds to Vstm2b while fully restoring binding to Vstm2l?
4. Does the impact of Vstm2b KO on basal synaptic transmission extend to long-term potentiation of Schaffer-collateral synapses in ventral CA1? It would be informative if LTP measurements were performed on female Vstm2b KO animals.

Reviewer #2

(Remarks to the Author)
The manuscript entitled "Control of Excitatory Synapse Maintenance in Ventral Hippocampal Circuits by Neurexin2α Ligand Vstm2b" presents a comprehensive and compelling study that identifies Vstm2 family members as novel α-neurexin-specific interactors, with a detailed focus on the role of Vstm2b in regulating ventral hippocampal excitatory synapse maintenance in a sex- and input-dependent manner. The work is conceptually significant, technically rigorous, and multidisciplinary, spanning proteomics, biochemistry, structural modeling, cellular assays, electrophysiology, and behavior. The findings

substantially advance our understanding of how synaptic adhesion molecules contribute to circuit-specific and sexually dimorphic synaptic organization and function. Overall, the manuscript is suitable for publication following careful consideration of the points below, which are intended to strengthen the clarity, interpretation, and impact of the study.

Major Points:

1. Mechanistic link between synaptic defects and behavior: While the study elegantly demonstrates synaptic phenotypes in the vCA1 and associated behavioral changes in Vstm2b KO mice, a direct causal relationship remains correlative. The authors appropriately suggest future experiments (e.g., region-specific knockdown) in the Discussion. To strengthen the current manuscript, please consider including a more detailed discussion on the potential circuit mechanisms linking vCA1 synaptic deficits to the observed anxiety and social behavior phenotypes. For instance, how might impaired SC-vCA1 or TA-vCA1 transmission specifically influence downstream targets (e.g., prefrontal cortex, amygdala) to modulate these behaviors? Elaborating on these potential pathways would enhance the conceptual framework.

2. Clarity on Vstm2b expression and localization: The data convincingly show Vstm2b enrichment in vCA1 pyramidal cells and its presence in synaptosomal fractions. However, the precise subsynaptic localization (pre- vs. postsynaptic) in vivo remains partially unclear due to antibody limitations. The authors acknowledge this. In the text (e.g., Results section "Vstm2b is expressed in CA1 pyramidal neurons..." and corresponding Discussion), please temper claims about "postsynaptic Vstm2b" (e.g., page 12, line 340) to reflect that while the heterologous synapse formation assay suggests a postsynaptic organizer function, the in vivo localization to the postsynaptic compartment is strongly supported but not yet definitively proven at the ultrastructural level. A more nuanced phrasing would be appropriate.

3. Statistical reporting and sample sizes: The manuscript generally employs appropriate statistical tests. However, for some key figures (e.g., Figure 8 electrophysiology, Figure 10 behavior), the sample sizes (n) are reported as number of mice, which is good. It would be helpful to also clarify in the figure legends or methods whether data points within an animal were averaged and what the "n" represents for cell-based assays (e.g., in situ binding, heterologous synapse formation). Additionally, for behavioral analyses in Figure 10, please confirm that the statistical tests accounted for any potential litter effects, especially given the use of littermate controls. A brief mention in the Methods would suffice.

4. Interpretation of the SLM synapse density/function disconnect: The finding that synapse density is reduced in the SLM of both sexes without a corresponding decrease in fEPSP strength is intriguing and well-discussed in the context of homeostatic plasticity. This is a strength of the paper. To further solidify this interpretation, could the authors provide (or discuss) any data or citations regarding the baseline release probability or quantal parameters of TA-vCA1 synapses compared to SC-vCA1? Even a speculative discussion on whether TA inputs might have a higher initial release probability or different AMPAR composition that could mask a loss of synapses would be insightful.

5. Sex as a biological variable: The study excels in its thorough integration of sex-based analyses throughout, which is a major strength. The authors have largely followed SAGER guidelines. Please ensure that the title and/or abstract explicitly state that key findings are sex-specific (they currently do in the abstract but could be emphasized in the title, e.g., "...reveals sexually dimorphic regulation of..."). Also, in the Methods description for animal studies, please explicitly state that both sexes were included in all experiments and analyses, and that sex was considered in the study design.

Minor Points and Clarifications:

1. Figure 1F & throughout: The saturation binding curves are valuable. Please state the exact n (number of independent experiments/cells) in the figure legend for panel F.
2. Figure 5A: RT-qPCR shows stable postnatal expression. Are there any significant differences between time points for each region? A statistical annotation (even ns) would be helpful.
3. Page 10, lines 271-272: "Vstm2b is present at both pre- and postsynaptic sites..." This conclusion seems primarily based on fractionation. Given the caveat about localization, consider rephrasing to "Vstm2b is present in synaptic fractions containing both pre- and postsynaptic markers..."
4. Figure 8 & 9 electrophysiology traces: The scale bars in the insets are sometimes hard to read. Ensuring consistent and clear labeling would improve accessibility.
5. Discussion, page 17, lines 495-503: The speculation on Humanin (Hn) is interesting. Please add a sentence acknowledging that the connection between Vstm2b and Hn signaling is currently hypothetical and requires direct experimental testing.
6. Methods - Statistical analysis: Please specify which post-hoc test was used following ANOVA (e.g., Šidák's, Tukey's).
7. Language: The manuscript is generally well-written. There are a few minor grammatical errors or awkward phrases (e.g., page 4, line 74: "αLNS6/βLNS allows assembly" might be clearer as "the αLNS6/βLNS domain allows assembly"; page 14, line 384: "female-specific reduction" is clear but check for similar phrasing consistency). A thorough proofread is recommended.

In summary, this is an outstanding study that makes a significant contribution to the fields of synapse biology and neural circuit mechanism. The identification of Vstm2b as a sex-dependent regulator of ventral hippocampal synapse maintenance via Nrxn2α is novel and important. The authors are commended on a rigorous and multifaceted approach. Addressing the major points above, particularly regarding the mechanistic discussion and clarity of claims, will further enhance the impact and clarity of this already impressive work. I support publication pending these revisions.

Reviewer #3

(Remarks to the Author)
Chofflet et al. perform affinity purification-mass spectrometry using the extracellular domain (ECD) of splice site 2 (SS2)-containing Neurexin-2alpha (Nrxn2aSS2+) as bait and identify Vstm2 immunoglobulin superfamily members as putative interactors. The authors perform an in-depth characterization of the interaction of Nrxn2aSS2+ with Vstm2 family members Vstm2a, Vstm2b and Vstm2l. They find that inclusion of SS2 is required for the interaction with Vstm2 and that Vstm2b only interacts with Nrxn2aSS2+, but not with Nrxn1a or Nrxn3a. In mixed culture assays, the authors find that Vstm2b expressed on the surface of heterologous cells can induce presynaptic differentiation in contacting axons, in a Nrxn2a-dependent manner. Expression of Vstm2b is highly enriched in pyramidal neurons in the ventral CA1 (vCA1) region of the hippocampus. The authors then characterize constitutive Vstm2b knockout (KO) mice, focusing on vCA1. They find sex- and input-specific differences in synaptic puncta density and synaptic transmission, as well as sex-specific behavioral changes, pointing to a role for Vstm2b in vCA1.

Taken together, this study identifies Vstm2b as a novel binding partner of Nrxn2aSS2+ and shows that Vstm2b contributes to synaptic function and maintenance in vCA1. The paper adds to a growing literature on splice site-specific interactors of neurexins and provides first insight into the synaptic function of Vstm2b. The experiments in the study are generally well performed. This is a data-rich and figure-heavy paper, in part due to the extensive literature on neurexins, which requires many experiments to compare results to previous findings. Some data (e.g. Fig. 7 as one example) could be moved to the supplement to improve the flow of the manuscript. The following points should be addressed.

Major Points
1. The authors characterize the interaction of Nrxn2a with Vstm2 proteins using COS-7 cells and a cell-free pull-down assay (Figs. 1 and 2). To rigorously and quantitatively analyze the interaction between purified Nrxn2aSS2+ and Vstm2 proteins, it is important to analyze the interaction using a biophysical method such as SPR, ITC, or other. The same quantitative approach should be used to determine whether the SS2 insert acts as a switch or as a modulator of the interaction with Vstm2 proteins.
2. Fig. 6: the VGLUT1 and VGAT puncta look unusually fuzzy and show high background, especially in panels 6G and 6I. It is not clear whether these puncta represent synaptic puncta. These experiments should be repeated, with better imaging quality. How do the authors explain the residual synapses on Vsmt2b-expressing cells co-cultured with Nrxn2a KO neurons (Fig. 6G, H)?
3. The subcellular fractionation suggests Vstm2b localizes to synaptic fractions, but biochemical analysis does not give a clear indication whether Vstm2b is postsynaptic at excitatory or inhibitory synapses. Given that little is known about Vstm2b and its role at synapses, the subcellular localization of endogenous Vstm2b at excitatory and inhibitory synapses needs to be demonstrated convincingly. If antibodies to Vstm2b are not suitable for detecting Vstm2b at the immunocyto- or histochemical level, the authors should perform CRISPR/Cas9 knock-in of an epitope tag to characterize subcellular localization.
4. I understand that the authors use a global KO mouse for Vstm2b for a first characterization of the role of this protein. However, this approach does not allow to draw direct conclusions about a postsynaptic role of Vstm2b at excitatory synapses: there is a possibility that some of the observed phenotypes are indirect consequences of Vstm2b loss in the brain or reflect Nrxn-independent roles of Vstm2b. Conclusive evidence that Vstm2b is functionally required at excitatory postsynapses is required, ideally showing that this is Nrxn2a-dependent. If the authors have a Vstm2b floxed mouse, targeted deletion of Vstm2b will provide insight. In the absence of a floxed Vstm2b mouse, a rescue strategy with Vstm2b in the global Vstm2b KO could be an alternative approach. If the authors have identified Vstm2b mutations that prevent interaction with Nrxn2a (based on their model in Fig. 3), rescue with such Vstm2b mutants could be insightful.

Minor Points
1. Line 536: “These findings are consistent with proteomic evidence identifying Vstm2a, but not Vstm2l or Vstm2b, in the neuronal mouse brain secretome” - The authors’ data suggests that Vstm2l is not expressed on the surface, yet this is the family member that is identified in the Nrxn2a ECD AP-MS approach in Fig. 1. How do the authors explain this?

2. Bar graphs in Fig. 2 should be shown differently: indicating individual datapoints (as in e.g. Fig. 4) or as box-whisker plots.

3. Why does the L360E Nrxn2a mutant enhance binding to Vstm2b?

4. Fig 4A, B: what is the negative control in this experiment (e.g. untransfected cells)?

5. Fig. 4D: is this MS data from N=1 surface biotinylation experiment? Please indicate in the legend.

6. Fig. 4F: why is GAPDH detected in the culture medium?

7. Fig. 5E, F: Vstm2b immunoreactivity on western blot shows multiple bands. The green arrowhead points to the correct band, but it is not clear whether only the upper band is considered to represent Vstm2b, or also the lower band. How did the authors determine that these bands indeed represent Vstm2b? Was this with the blot shown in Fig. S6D? If so, this should be indicated at this point in the text.

8. Fig. 6L: the IgG-coated control beads are not touching neurites in the example image shown. How does this look when they do touch neurites? Do Vstm2b-coupled beads induce VGLUT1 clustering in Nrxn2a KO cultures?

9. Supplementary Fig. 7: “… did not detect Nrxn2β-specific-peptides (Supplementary Fig. 7)”. It is not entirely clear how this

was concluded: the figure shows common sequences between Nrxn2a and Nrn2b as underlined but sequence unique to Nrxn2b is not shown: do the red sequences shown represent all identified peptides?

10. Have the authors analyzed effects of Vmst2b KO on inhibitory synapse density or synaptic transmission?

11. Line 450: "… the Vstm2b-Nrxn2a complex emerges as a key regulator of ventral hippocampal glutamatergic circuits, providing a mechanism for sexually dimorphic synapse specificity and circuit function." This conclusion seems a bit overstated; the mechanism underlying the sexual dimorphic effects remains unclear. Are there differences in Vstm2b expression patterns in male vs. female mice?

12. Small typo: Fig. 1E legend: 'pull-downed'; line 440: a period seems to be missing between 'LNS2 domain' and 'Vmst2b'.

Version 1:

Reviewer comments:

Reviewer #1

(Remarks to the Author)
The authors have thoroughly addressed my concerns.

Reviewer #2

(Remarks to the Author)
I have carefully reviewed the revised manuscript and the detailed point-by-point response to the reviewers' comments. The authors have undertaken a substantial amount of additional experimental work and textual revision, which has significantly strengthened the manuscript. My assessment of how the key concerns have been addressed is as follows:

New Experimental Data
The authors have generated an impressive set of new data in response to reviewer critiques:
1. Inhibitory synapse analysis (Supp Fig. 16): Immunohistochemistry for VGAT/gephyrin in vCA1 subregions confirms that Vstm2b deletion does not alter inhibitory synapse density in either sex. This convincingly demonstrates that Vstm2b's effects are specific to excitatory synapses.
2. SPR binding data (Supp Fig. 3): Biophysical validation of the Vstm2b–Nrxn2α interaction by surface plasmon resonance (KD = 3.65 ± 0.13 μM) provides quantitative rigor beyond the cell-based binding assays, and confirms the absolute requirement for SS2 inclusion.
3. Ca^{2+} independence (Supp Fig. 4): The D335A Ca^{2+}-binding-deficient mutant experiments are elegant. The unexpected fourfold enhancement of Vstm2b binding by D335A is scientifically interesting and rigorously demonstrates Ca^{2+} independence, consistent with the EGTA experiments.
4. Revised artificial synapse assays (Fig. 6, Supp Figs. 10–11): The bead-based and HEK-cell-based approaches with single immunostaining convincingly show selective VGLUT1 (not VGAT) induction. Image quality is markedly improved, and the complete loss of VGLUT1 clustering in Nrxn2α KO neurons definitively establishes Nrxn2α as the functional presynaptic receptor.
5. Postsynaptic spine phenotype (Fig. 6G–J): The mixed neuron coculture experiments elegantly isolate pre- vs. postsynaptic contributions, demonstrating that postsynaptic Vstm2b loss reduces mature spine density. The competitive nature of the phenotype (no effect in KO–KO cocultures) is consistent with similar findings for Nlgn1 and adds mechanistic depth.
6. mEPSC recordings (Fig. 8, Supp Fig. 18): Single-cell patch-clamp data showing reduced mEPSC frequency specifically in female KO mice corroborates the fEPSP and synapse density phenotypes and reveals intrinsic sex differences in vCA1 synaptic properties that are abolished by Vstm2b deletion.
7. Subcellular targeting (Supp Fig. 9): Exogenous HA-Vstm2b shows broad axonal and dendritic distribution without strong compartmental bias, consistent with its detection in both pre- and postsynaptic biochemical fractions.

Assessment of Major Concerns from Previous Review
Methodological rigor: The addition of SPR, D335A mutant analysis, and the bead-based synapse formation assays substantially elevates the quantitative and mechanistic rigor of the binding characterization. I consider this concern fully addressed.
Synapse specificity and localization: The new inhibitory synapse quantification data and the revised artificial synapse assays compellingly establish that Vstm2b selectively organizes glutamatergic synapses. The tempered language regarding postsynaptic localization accurately reflects both the strength and limitations of current evidence. The authors' candid description of extensive but unsuccessful efforts to detect endogenous Vstm2b (multiple antibodies, CRISPR knock-in attempts) is appreciated. This concern is adequately addressed.
Global KO limitations: The mixed coculture experiments demonstrating a postsynaptic requirement for Vstm2b in spine maintenance, combined with the mEPSC data at the single-neuron level, provide substantial functional evidence that partially mitigates the inherent limitations of global KO approaches. The authors appropriately discuss the need for conditional deletion strategies in future work.
Circuit–behavior link: The expanded Discussion now provides a well-reasoned framework connecting vCA1 synaptic deficits to anxiety and social behavior phenotypes through known vCA1 projections to the nucleus accumbens and lateral

hypothalamic area. The authors appropriately acknowledge that direct causal evidence requires future region-specific manipulations.
SLM synapse density/function disconnect: The revised Discussion now includes a thorough and well-referenced comparison of SR vs. SLM synaptic properties (release probability, AMPAR expression, homeostatic capacity), providing a plausible mechanistic explanation for the dissociation between reduced synapse density and preserved fEPSP strength.

SAGER Guidelines Compliance
The revised manuscript complies well with SAGER guidelines:
The title now explicitly states "Sexually Dimorphic Control"
The abstract clearly indicates sex-specific findings
Methods section explicitly states both sexes were included and analyzed separately
Data are reported disaggregated by sex throughout
Intrinsic sex differences in WT mice are reported (Supp Fig. 18)

Minor Outstanding Points
1. GAPDH in culture medium (Supp Fig. 4F-related): The authors provide a reasonable justification with literature references for extracellular GAPDH. While acceptable, this could be noted as a minor caveat in the figure legend.
2. LTP measurements: The authors acknowledge that systematic LTP analysis at SC–vCA1 and TA–vCA1 synapses would be informative. This is appropriately deferred to future studies, and does not diminish the present findings given that basal synaptic transmission and synapse density phenotypes are already well-characterized.
3. N=2 for surfaceome MS (Fig. 4E): The authors have now clarified this in the figure legend. Given the confirmatory nature of these data (validated by independent biochemical approaches), this is acceptable.
4. Humanin speculation: The revised Discussion now explicitly states that the Vstm2b–Humanin connection is hypothetical and requires experimental testing. This is appropriately tempered.

Overall Assessment
This is an outstanding study that makes a significant and original contribution to the synapse biology field. The identification and characterization of Vstm2b as a sex-dependent, input-specific regulator of ventral hippocampal excitatory synapse maintenance via Nrxn2α is conceptually novel and experimentally rigorous. The revisions have substantively addressed all major concerns, with extensive new experimental data that both strengthen the core conclusions and add mechanistic depth. The manuscript is well-written, the figures are clear, and the statistical reporting is now appropriately detailed.
I recommend acceptance for publication in Nature Communications.

Reviewer #3

(Remarks to the Author)
The authors have carefully addressed my major concerns: the SPR experiment adds a rigorous quantification of the interaction, strengthening the findings in the manuscript. The repeated and extended coculture assays now provide convincing evidence for a specific induction of excitatory presynaptic specialisations and dependence on Nrxn2a. The efforts to attempt endogenous tagging of Vstm2b unfortunately did not work out, but the low-moderate expression approach of a tagged construct is a good compromise that adds valuable insight in the distribution of exogenously expressed Vstm2b. The added culture experiment, using various ratios of wildtype and Vstm2b KO neurons, is a strong addition to support a postsynaptic role of Vstm2b. My minor concerns are equally well addressed. I therefore fully support publication of this interesting study and congratulate the authors with their careful and rigorous work.
In reviewing the revisions, I noted the following minor issues that the authors can easily correct in the final version of their manuscript:
-legend Fig. S3 contains a small typo: 'surface plasma' should be 'surface plasmon'; in addition, the labels in the graphs should be changed from 'um' to 'uM'
-response to minor point #5: 2 independent culture wells from the same neuronal culture in my view should be represented in the figure legend as 2 technical replicates, not 2 biological replicates. For biological replicates, I would consider 2 independently prepared neuronal cultures to represent biological replicates.

Open Access This Peer Review File is licensed under a Creative Commons Attribution 4.0 International License, which permits use, sharing, adaptation, distribution and reproduction in any medium or format, as long as you give appropriate credit to the original author(s) and the source, provide a link to the Creative Commons license, and indicate if changes were made.
In cases where reviewers are anonymous, credit should be given to 'Anonymous Referee' and the source.
The images or other third party material in this Peer Review File are included in the article's Creative Commons license, unless indicated otherwise in a credit line to the material. If material is not included in the article's Creative Commons license and your intended use is not permitted by statutory regulation or exceeds the permitted use, you will need to obtain permission directly from the copyright holder.
To view a copy of this license, visit https://creativecommons.org/licenses/by/4.0/

Authors' response to the reviewers' comments for the manuscript **NCOMMS-26-000546** of Chofflet N. et al., **"Control of Excitatory Synapse Maintenance in Ventral Hippocampal Circuits by Neurexin2α Ligand Vstm2b"**, and description of changes made in the revised paper

First of all, we thank all the reviewers for their careful reviewing and helpful suggestions. We performed additional experiments to answer the reviewers' questions and to strengthen our conclusions. These new data have been integrated into our revised manuscript. The point-by-point answers are described below with the reviewers' comments in *italic* font and the responses and descriptions of changes made in the manuscript in bold font. Page and line numbers referenced in this response letter correspond to the PDF version of the revised manuscript. Further, the changes in the manuscript text file are highlighted in red.

Reviewer #1 (Remarks to the Author):

In this manuscript by Chofflet and colleagues, the authors identify and characterize Vstm2b, a novel extracellular ligand for neurexin2a. Using a diverse set of biochemical, molecular, cellular, computational, electrophysiological, and behavioral assays, the authors convincingly identify Vstm2b as a ligand for neurexin2a. Using an unbiased screen, the authors identified Vstm2l as a putative Nrxn2a interacting protein. Vstm2l is a member of the Vstm family, which also includes Vstm2a and Vstm2b. Vstm2a and Vstm2b are expressed in the brain and are presented to the extracellular space via a GPI-anchor. However, Vstm2l is present on the membrane, yet robustly enriched with the Nrxn2a bait. The authors primarily focus on the properties of Vstm2b due to its specific interaction with Nrxn2a - it selectively binds LNS2, and is dependent on the inclusion of the SS2 alternative exon. When expressed in non-neuronal cells, Vstm2b robustly induces excitatory and inhibitory presynaptic assembly in cultured neurons. Further, the assembly of excitatory presynaptic terminals in this assay is dependent on presynaptic Nrxn2a. In neurons, Vstm2b regulates the number of synapses in ventral CA1 in a sex-dependent manner. This synapse number phenotype is not due the impaired synaptogenesis, but synapse maintenance. Functionally, global ablation of Vstm2b led to reduced basal excitatory transmission at Schaffer-collateral synapses in females but not males. Importantly, in young animals, the deletion of Vstm2b did not alter synapse numbers, but impaired basal synaptic transmission, which suggest

that the synapse number phenotype in adult animals might be a consequence of impaired excitatory synapse function. Finally, using different experimental paradigms to study anxiety-like behavior, the authors report reduced anxiety-like behavior in female Vstm2b KO animals. Overall, this is well-done study that importantly expands our understanding of a-Nrxn-specific signaling at the synapse and provides additional insight into the role of Nrxn2a, arguably the least well characterized alpha-neurexin. The conclusions of the paper are supported by strong data and, as such, I only have minor comments.

1. Vstm2b is also expressed in GABAergic neurons (Suppl Fig. 6C) and recruits vGAT-positive terminals in the co-culture assay (Fig. 6). The authors should measure GABAergic markers in ventral CA1 of Vstm2b KO hippocampi with region-specificity (e.g. soma vs apical dendrite).

Response:

We performed immunohistochemistry for VGAT and gephyrin and quantified the density of their colocalized puncta, which represent inhibitory synapses, in the *stratum pyramidale* (SP), *stratum radiatum* (SR) and *stratum lacunosum moleculare* (SLM) of the vCA1 in *Vstm2b* knockout (KO) and wild-type (WT) mice of both sexes. We found no significant differences in inhibitory synapse number in the vCA1 SP, SR, or SLM of either sex, indicating that Vstm2b primarily regulates excitatory, rather than inhibitory, synapse density. This result has been added to the revised Results section as Supplementary Figure 16.

In addition, to address Reviewer 3's major comment #2 and minor comment #10, we repeated the artificial synapse formation assays using Vstm2b-Fc-coated beads as well as HEK cells expressing Vstm2b, followed by single immunostaining for VGLUT1 or VGAT. These experiments showed that Vstm2b selectively induced VGLUT1, but not VGAT, accumulation, as presented in Fig. 6 and Supplemental Fig. 10. This additional evidence indicates that Vstm2b contributes to excitatory, but not inhibitory, synapse organization.

2. To supplement the experiments in Suppl Fig. G-L, can the authors use a Ca2+-binding deficient LNS2 mutant to further demonstrate that the Vstm2b-Nrxn2a interaction is Ca2+ independent?

Response:

To address this suggestion, we generated a plasmid expressing the extracellular myc-tagged, membrane-anchored Nrxn2α LNS2 domain carrying a Ca^{2+} binding-deficient point

mutation (D335A). Asp335 in Nrxn2α (NP001192163.1) is the structural counterpart of Asp329 in Nrxn1α which we know is the key Ca^{2+} coordinating residue based on the 3D structure of Nrxn1α LNS2 in the Ca2+-containing form (PDB ID: 2H0B; PMID: 16772286). Using the Nrxn2α D335A mutant construct, we performed *in situ* protein binding assays to directly test whether the Vstm2b-Nrxn2α interaction is Ca^{2+} independent.

Unexpectedly, the D335A mutation significantly increased the binding of Vstm2b-Fc protein by approximately fourfold compared to the wild-type Nrxn2α LNS2 domain. This enhanced binding was not attributable to altered surface expression of myc-Nrxn2α LNS2 D335A (see Supplementary Fig. 4). These results indicate that the Vstm2b-Nrxn2α LNS2 interaction does not require extracellular Ca^{2+}, consistent with our EGTA treatment experiments (Supplementary Fig. 4), but the interaction cannot be considered strictly Ca^{2+} independent in the context of the experimental setup and technical details of the assay.

We further examined whether the D335A mutation affects the interaction between Neurexophilin 1 (Nxph1) and Nrxn2α LNS2. *In situ* binding assays showed that D335A had no detectable effect, consistent with previous findings that revealed Ca^{2+} independency of the interaction (Wilson SC et al., EMBO J., 2019; PMID: 31566781). Together with Fig. 3F and 3G, this comparison provides an additional mechanistic insight: Vstm2b engages the Nrxn2α LNS2 domain in a manner that is partially similar to, yet distinct from, Nxph1.

We acknowledge, however, that high-resolution structural characterization, such as crystallography or cryo-EM, of Vstm2b alone and in complex with Nrxn2α will be essential to fully elucidate the molecular basis of these interactions.

These new experimental results have been incorporated into the revised manuscript as Supplementary Figure 4.

3. Can the authors speculate why the LNS2SS2A+ only partially binds to Vstm2b while fully restoring binding to Vstm2l?

Response:

All three Vstm2 paralogs require the LNS2 domain of Nrxn2α for binding, as deleting LNS2 completely abolishes the interaction. When only the isolated LNS2 domain is expressed, all three proteins can bind; however, Vstm2l binds at levels similar to full-length Nrxn2α, whereas Vstm2a and Vstm2b show only partial binding. These binding assays were performed in parallel for all three Vstm2 proteins, using comparable protein

concentrations (500 nM). Given that Vstm2l has a higher affinity for Nrxn2α (Kd ~95 nM) compared to Vstm2b (Kd ≥1 μM) and Vstm2a (Kd ≥1.7 μM), the concentration used was near saturation for Vstm2l, which would mask any detectable difference in binding between full-length Nrxn2α and isolated LNS2. By contrast, the lower-affinity Vstm2a and Vstm2b proteins showed partial binding under the same conditions. Notably, the degree of binding correlates with the affinity of each protein: Vstm2l (highest affinity) binds fully, Vstm2b (intermediate affinity) binds partially at ~50%, and Vstm2a (lowest affinity) binds partially at ~25%.

In addition, we performed binding assays to assess the interaction of soluble Vstm2b with membrane-tethered Nrxn2α truncated variants. We found that constructs containing LNS1–LNS3 and LNS1–LNS2 domains bound Vstm2b at comparable levels, whereas the LNS2 domain alone showed a noticeable, though not statistically significant, reduction in binding. These results suggest that LNS2 primarily mediates the interaction, with potential contributions from adjacent domains either stabilizing the interaction or supporting an optimal structural context for Vstm2b binding. These new data are now included in the revised manuscript as part of Supplementary Figure 2G-H.

4. Does the impact of Vstm2b KO on basal synaptic transmission extend to long-term potentiation of Schaffer-collateral synapses in ventral CA1? It would be informative if LTP measurements were performed on female Vstm2b KO animals.

Response:

To date, we have not conducted LTP measurements; however, we consider a systematic analysis of LTP at SC–vCA1 synapses and TA–vCA1 synapses in both sexes at distinct developmental stages (P12 and P28) to be an important and compelling direction for future investigation. Such analyses are particularly relevant given the sex- and input-specific synaptic phenotypes associated with the impaired social recognition memory observed in both males and females.

Based on our current findings, we anticipate impaired LTP at SC-CA1 synapses at both P12 and P28 in female KO mice, as these synapses exhibit functional deficits as early as P12. We further hypothesize that LTP at TA-vCA1 synapses at P28 will be compromised in both sexes. This prediction is grounded in the likelihood that the available capacity of TA-

vCA1 synapses for postsynaptic AMPA receptor recruitment is already maximally engaged to compensate for the reduced synapse number.

<u>Reviewer #2 (Remarks to the Author):</u>

The manuscript entitled "Control of Excitatory Synapse Maintenance in Ventral Hippocampal Circuits by Neurexin2α Ligand Vstm2b" presents a comprehensive and compelling study that identifies Vstm2 family members as novel α-neurexin-specific interactors, with a detailed focus on the role of Vstm2b in regulating ventral hippocampal excitatory synapse maintenance in a sex- and input-dependent manner. The work is conceptually significant, technically rigorous, and multidisciplinary, spanning proteomics, biochemistry, structural modeling, cellular assays, electrophysiology, and behavior. The findings substantially advance our understanding of how synaptic adhesion molecules contribute to circuit-specific and sexually dimorphic synaptic organization and function. Overall, the manuscript is suitable for publication following careful consideration of the points below, which are intended to strengthen the clarity, interpretation, and impact of the study.

Major Points:

1. Mechanistic link between synaptic defects and behavior: While the study elegantly demonstrates synaptic phenotypes in the vCA1 and associated behavioral changes in Vstm2b KO mice, a direct causal relationship remains correlative. The authors appropriately suggest future experiments (e.g., region-specific knockdown) in the Discussion. To strengthen the current manuscript, please consider including a more detailed discussion on the potential circuit mechanisms linking vCA1 synaptic deficits to the observed anxiety and social behavior phenotypes. For instance, how might impaired SC-vCA1 or TA-vCA1 transmission specifically influence downstream targets (e.g., prefrontal cortex, amygdala) to modulate these behaviors? Elaborating on these potential pathways would enhance the conceptual framework.

Response:

While there is currently no clear evidence regarding layer-specific connectivity of relevant brain regions with vCA1 pyramidal neurons, we have expanded the Discussion to consider how vCA1 synaptic deficits may contribute to the observed anxiety and social behavior phenotypes. Specifically, we highlight the role of the vCA1 in regulating social memory and anxiety through its intra- and extra-hippocampal upstream inputs and downstream projections as follows:

Page 21-22, line 608-629: "While this study identifies synaptic defects in the vCA1 and associated behavioral abnormalities in *Vstm2b* KO mice, further work is necessary to directly test the causal relationship between these phenotypes. The ventral hippocampus, particularly the vCA1, has been strongly implicated in the regulation of social memory and anxiety-related behaviors. Previous studies have shown that vCA1 neuronal ensembles encode social memory and regulate social recognition through projections to downstream limbic regions such as the nucleus accumbens 47, while integrating information from upstream inputs including the dCA2 and the amygdala 62. Additionally, the vCA1 contains a subset of anxiety-responsive pyramidal neurons that are activated in anxiogenic environments and drive avoidance behavior 63, 64. Accordingly, optogenetic inhibition of vCA1 pyramidal neurons promotes exploration and produces anxiolytic effects, whereas activation of their projections to the lateral hypothalamic area (LHA) increases anxiety and avoidance 64. The selective enrichment of Vstm2b in vCA1 pyramidal neurons relative to other hippocampal excitatory neurons suggests that loss of *Vstm2b* in this population may drive cell-autonomous synaptic dysfunction and compromised synaptic integrity, potentially underlying at least part of the observed behavioral abnormalities. At the same time, Vstm2b is also expressed in other social- and anxiety-related regions, including the amygdala and the hypothalamus, as well as in hippocampal GABAergic interneurons. Targeted viral delivery of Cre recombinase into the vCA1 of *Vstm2b*-floxed mice, or conditional deletion of Vstm2b in glutamatergic or GABAergic neurons, would help elucidate whether and how region- or cell-type–specific loss of Vstm2b drives local circuit dysfunction within the vCA1 and contributes to behavioral impairments."

2. Clarity on Vstm2b expression and localization: The data convincingly show Vstm2b enrichment in vCA1 pyramidal cells and its presence in synaptosomal fractions. However, the precise subsynaptic localization (pre- vs. postsynaptic) in vivo remains partially unclear due to antibody limitations. The authors acknowledge this. In the text (e.g., Results section "Vstm2b is expressed in CA1 pyramidal neurons…" and corresponding Discussion), please temper claims about "postsynaptic Vstm2b" (e.g., page 12, line 340) to reflect that while the heterologous synapse formation assay suggests a postsynaptic organizer function, the in vivo localization to the postsynaptic compartment is strongly supported but not yet definitively proven at the ultrastructural level. A more nuanced phrasing would be appropriate.

Response:

We agree that definitive statements regarding postsynaptic localization should be nuanced given the current antibody limitations. We have therefore tempered the language in both the Results and Discussion sections as detailed below.

Results section - Page 11, line 311-313:
“These results indicate that Vstm2b is present in synaptic fractions containing both pre- and postsynaptic markers within the hippocampus.”

Results section - Page 14-15, line 403-406:
“Together with the predominant expression of Vstm2b in vCA1 pyramidal cells and its absence in vCA3 pyramidal cells (Fig. 5B-D), these results are consistent with a model in which Vstm2b engages in a trans-interaction with presynaptic Nrxn2α to the organization of SC–CA1 glutamatergic synapses in the ventral hippocampus.”

Discussion section-Page 23, line 663-667:
“Vstm2b expression and localization also remain incompletely defined: we detected endogenous Vstm2b in synaptosomal and postsynaptic fractions, but antibody limitations prevented the precise determination of layer-specific expression or subcellular and subsynaptic localization in the vCA1 at the ultrastructural level.”

We believe that these revisions more accurately reflect the strength and limitations of the current evidence while preserving the overall interpretation of our findings.

3. Statistical reporting and sample sizes: The manuscript generally employs appropriate statistical tests. However, for some key figures (e.g., Figure 8 electrophysiology, Figure 10 behavior), the sample sizes (n) are reported as number of mice, which is good. It would be helpful to also clarify in the figure legends or methods whether data points within an animal were averaged and what the "n" represents for cell-based assays (e.g., in situ binding, heterologous synapse formation). Additionally, for behavioral analyses in Figure 10, please confirm that the statistical tests accounted for any potential litter effects, especially given the use of littermate controls. A brief mention in the Methods would suffice.

Response:

We have now clarified the definition of n for all experiments in the Methods section of the revised manuscript.

For the behavioral experiments, each n corresponds to a single measurement from one mouse. For the quantification of synapse number in the ventral CA1, each n represents one mouse; for each mouse, 4-6 images were collected from at least three independent brain slices and averaged to yield a single value per animal. For field excitatory postsynaptic synaptic potential (fEPSP) recordings, each n also represents one mouse. For each mouse, 3-5 slices were recorded, and 3-5 trials per stimulation condition (3 trials for SR, 5 trials for SLM) were averaged within each slice. Input-output and paired pulse protocols were performed at the same locations, and values were averaged across slices to generate a single data point per mouse. For miniature excitatory postsynaptic current (mEPSC) recordings, each n represents one patched neuron. For the cell-based assays (e.g., *in situ* binding and artificial synapse formation assays), n corresponds to the number of analyzed cells obtained from multiple independent experiments. This is standard practice in the field of *in vitro* synapse biology, where individual cells constitute independent experimental units and multiple independent cultures are included for each condition.

Regarding potential litter effects in the behavioral analyses, we did not include litter as a statistical factor. However, our experimental design was structured to minimize litter related confounds. Each behavioral cohort included mice derived from at least six independent litters, with balanced numbers of WT and KO littermates within each litter, and animals from both genotypes were always tested in parallel. No cohort included mice of only one genotype from a given litter. This balanced littermate-controlled design reduces the likelihood of litter driven effects. We have now added the following clarification to the Methods section:

Page 41, line 1219-1222: "To minimize potential litter effects, each behavioral test included mice derived from at least six independent litters. In each cohort, animals from both genotypes were tested in parallel, with balanced representation of WT and KO mice from each litter."

4. Interpretation of the SLM synapse density/function disconnect: The finding that synapse density is reduced in the SLM of both sexes without a corresponding decrease in fEPSP strength is intriguing and well-discussed in the context of homeostatic plasticity. This is a strength of the paper. To further solidify this interpretation, could the authors provide (or discuss) any data or citations regarding the baseline release probability or quantal parameters of TA-vCA1 synapses

compared to SC-vCA1? Even a speculative discussion on whether TA inputs might have a higher initial release probability or different AMPAR composition that could mask a loss of synapses would be insightful.

Response:

Given this encouraging suggestion, we revised the appropriate Discussion paragraph as highlighted in red below.

Page 20, line 564-587: "While the reduction in synapse density in the SR correlates with decreased SC-vCA1 synaptic strength in *Vstm2b* KO female mice, this correlation is not observed for TA-vCA1 inputs in the SLM of either sex. Although data from the ventral CA1 are limited, studies in the dorsal CA1 indicate that the SR and SLM differ markedly: the SLM has fewer excitatory and relatively more inhibitory synapses than the SR 53, and although perforated synapses are more prevalent, AMPAR expression is lower 53, 54. In addition, excitatory synapses in the SLM exhibit lower baseline release probability than their SR counterparts, in part due to a reduced contribution of N-type voltage-gated Ca^{2+} channels to neurotransmitter release; however, this N-type channel contribution can be enhanced during synaptic potentiation through NMDAR- and L-type Ca^{2+} channel–dependent mechanisms 55, indicating that TA synapses retain a substantial capacity for activity-dependent strengthening. Consistently, TA inputs also exhibit input-specific homeostatic plasticity: while lesions of EC neurons reduce excitatory synapse density in the SLM, both lesions and chemogenetic silencing of these neurons increase spontaneous synaptic currents in the CA1, an effect not seen following SC lesions 56. Similarly, amino-oxyacetic acid (AOAA)-induced partial neuronal loss in EC layer III does not significantly reduce TA–CA1 efficacy, suggesting that even a modest number of intact synapses can sustain physiologically normal fEPSPs 57. Together, these findings suggest that the homeostatic properties of TA-CA1 synapses may account for the absence of an fEPSP phenotype in *Vstm2b* KO mice, despite a significant reduction in the number of excitatory synapses in the SLM. Future studies such as pharmacological inhibition of N-type Ca^{2+} channels during SLM fEPSP recordings could help clarify the mechanisms underlying the apparent dissociation between reduced synapse density and preserved basal synaptic transmission in *Vstm2b* KO mice."

5. Sex as a biological variable: The study excels in its thorough integration of sex-based analyses throughout, which is a major strength. The authors have largely followed SAGER guidelines.

Please ensure that the title and/or abstract explicitly state that key findings are sex-specific (they currently do in the abstract but could be emphasized in the title, e.g., "...reveals sexually dimorphic regulation of..."). Also, in the Methods description for animal studies, please explicitly state that both sexes were included in all experiments and analyses, and that sex was considered in the study design.

Response:

In response, we have revised the title to explicitly emphasize the sex specific nature of our findings:

Revised title: **Sexually Dimorphic Control of Excitatory Synapse Maintenance in Ventral Hippocampal Circuits by the Neurexin2α Ligand Vstm2b**

We have also updated the Methods section to clearly state that both sexes were included in all *in vivo* experiments and that sex was incorporated into the study design:

Page 24, lines 692-694: "All ex and in vivo experiments and analyses included both male and female mice, and data were analyzed separately by sex. Sex was considered in the study design."

Minor Points and Clarifications:

1. Figure 1F & throughout: The saturation binding curves are valuable. Please state the exact n (number of independent experiments/cells) in the figure legend for panel F.

Response:

For the *in situ* protein binding assays and heterologous synapse formation assays, a large number of experimental conditions and quantified cells were analyzed across multiple experiments. Listing the exact number of analyzed cells for every individual condition directly in each figure legend would substantially increase their length and reduce readability. Instead, as stated in the Fig. 1F legend, we report the minimum number of analyzed cells and the number of independent experiments for each assay (e.g., n ≥ 30 cells from three independent experiments), which provides a clear and consistent indication of sampling depth.

Furthermore, to ensure full transparency, we have provided a comprehensive "Source data" file containing all individual measurements underlying each figure. This allows

readers to see the exact sample number and the value of every data point corresponding to the analyses presented.

In addition, following the reviewer's suggestion, we have explicitly included the number of analyzed mice for the ex *vivo* synapse number measurements and for electrophysiological recordings in both the main and supplementary figure legends.

2. Figure 5A: RT-qPCR shows stable postnatal expression. Are there any significant differences between time points for each region? A statistical annotation (even ns) would be helpful.

Response:

We addressed this point by performing two-way ANOVAs followed by Tukey's multiple comparisons test to compare expression levels between P0 and P56 for each transcript in each brain region. Figure 5A now includes the P values for these statistical comparisons.

3. Page 10, lines 271-272: "Vstm2b is present at both pre- and postsynaptic sites..." This conclusion seems primarily based on fractionation. Given the caveat about localization, consider rephrasing to "Vstm2b is present in synaptic fractions containing both pre- and postsynaptic markers..."

Response:

Our conclusion is based on subcellular fractionation of mouse brain. Synaptosomes were further subfractionated into Triton-soluble and Triton-insoluble fractions, which are commonly described as postsynaptic and presynaptic compartments, respectively. We validated these fractions using established markers: the Triton-soluble fraction is positive for the presynaptic marker Synaptophysin (SYP) and negative for postsynaptic markers PSD95 and Gephyrin (GPHN) as presynaptic fraction, whereas the Triton-insoluble fraction is negative for SYP and positive for PSD95 and GPHN as postsynaptic fraction. This validation supports that endogenous Vstm2b is expressed in both pre- and post-synaptic fractions.

Additionally, we noticed an error in the original Figure 5F: the bottom lane of the immunoblot was labeled as SYT1 instead of SYP. This has been corrected in the updated Figure 5.

To further address subcellular targeting of Vstm2b, we transfected primary rat neuron cultures with a plasmid expressing extracellular HA-tagged Vstm2b (HA-Vstm2b) and imaged transfected neurons with low-to-middle expression level of HA-Vstm2b. We quantified the axon-to-dendrite distribution ratio of HA-Vstm2b and found that Vstm2b can be targeted to both compartments without strong compartmental restriction. The new data has been included in the revised manuscript as Supplementary Figure 9.

Accordingly, we have revised the text in the Results section to emphasize that Vstm2b is present in both presynaptic and postsynaptic fractions:

Page 11, line 307-311: "Vstm2b was detected in synaptosomes and in both Triton-soluble and Triton-insoluble fractions, which correspond to pre- and postsynaptic compartments, respectively, as validated by compartment-specific markers (Triton-soluble: positive for Synaptophysin (SYP), negative for PSD95 and Gephyrin (GPHN); Triton-insoluble: negative for SYP, positive for PSD95 and GPHN) (Fig. 5F)."

4. Figure 8 & 9 electrophysiology traces: The scale bars in the insets are sometimes hard to read. Ensuring consistent and clear labeling would improve accessibility.

Response:

We have revised the electrophysiology traces in Figures 7 and 9 to ensure that all inset scale bars are clearly visible and consistently labeled, improving clarity and accessibility.

5. Discussion, page 17, lines 495-503: The speculation on Humanin (Hn) is interesting. Please add a sentence acknowledging that the connection between Vstm2b and Hn signaling is currently hypothetical and requires direct experimental testing.

Response:

Given this comment, we revised the corresponding paragraph as follows. Page 21, lines 595-606: "On the other hand, the mechanisms underlying sex-dependent maturation and maintenance of the SC-CA1 and TA-CA1 synapses remain unknown. Interestingly, astrocyte-released Humanin (Hn) plays a critical role in synapse maintenance and function, with its secretion regulated by ovarian hormones 60. Moreover, Vstm2l acts as an Hn ligand and modulates its activity 61, leading to the intriguing hypothesis that Vstm2b may also interact with Hn signaling to confer sexually dimorphic synapse

maturation and maintenance. Future studies are required to determine whether Vstm2b physically and functionally interact with Hn. Furthermore, assessing synaptic density and function in gonadectomized Vstm2b WT and KO mice, as well as evaluating Nrxn2α expression and its SS2 splicing levels in these mice, may provide further insight into the mechanisms through which Vstm2b contributes to hippocampal sexual dimorphism."

6. Methods - Statistical analysis: Please specify which post-hoc test was used following ANOVA (e.g., Šidák's, Tukey's).

Response:

In the revised manuscript, *post-hoc* tests for all ANOVA analyses are specified in the corresponding figure legends.

7. Language: The manuscript is generally well-written. There are a few minor grammatical errors or awkward phrases (e.g., page 4, line 74: "αLNS6/βLNS allows assembly" might be clearer as "the αLNS6/βLNS domain allows assembly"; page 14, line 384: "female-specific reduction" is clear but check for similar phrasing consistency). A thorough proofread is recommended.

Response:

We have incorporated the recommended revisions, including clarifying the phrases (e.g., revising "αLNS6/βLNS allows assembly" to "the αLNS6/βLNS domain allows assembly"), and we have thoroughly proofread the entire manuscript to ensure consistency and clarity throughout.

In summary, this is an outstanding study that makes a significant contribution to the fields of synapse biology and neural circuit mechanism. The identification of Vstm2b as a sex-dependent regulator of ventral hippocampal synapse maintenance via Nrxn2α is novel and important. The authors are commended on a rigorous and multifaceted approach. Addressing the major points above, particularly regarding the mechanistic discussion and clarity of claims, will further enhance the impact and clarity of this already impressive work. I support publication pending these revisions.

Reviewer #3 (Remarks to the Author):

Chofflet et al. perform affinity purification-mass spectrometry using the extracellular domain (ECD) of splice site 2 (SS2)-containing Neurexin-2alpha (Nrxn2aSS2+) as bait and identify Vstm2 immunoglobulin superfamily members as putative interactors. The authors perform an in-depth characterization of the interaction of Nrxn2aSS2+ with Vstm2 family members Vstm2a, Vstm2b and Vstm2l. They find that inclusion of SS2 is required for the interaction with Vstm2 and that Vstm2b only interacts with Nrxn2aSS2+, but not with Nrxn1a or Nrxn3a. In mixed culture assays, the authors find that Vstm2b expressed on the surface of heterologous cells can induce presynaptic differentiation in contacting axons, in a Nrxn2a-dependent manner. Expression of Vstm2b is highly enriched in pyramidal neurons in the ventral CA1 (vCA1) region of the hippocampus. The authors then characterize constitutive Vstm2b knockout (KO) mice, focusing on vCA1. They find sex- and input-specific differences in synaptic puncta density and synaptic transmission, as well as sex-specific behavioral changes, pointing to a role for Vstm2b in vCA1.

Taken together, this study identifies Vstm2b as a novel binding partner of Nrxn2aSS2+ and shows that Vstm2b contributes to synaptic function and maintenance in vCA1. The paper adds to a growing literature on splice site-specific interactors of neurexins and provides first insight into the synaptic function of Vstm2b. The experiments in the study are generally well performed. This is a data-rich and figure-heavy paper, in part due to the extensive literature on neurexins, which requires many experiments to compare results to previous findings. Some data (e.g. Fig. 7 as one example) could be moved to the supplement to improve the flow of the manuscript. The following points should be addressed.

Major Points

1. The authors characterize the interaction of Nrxn2a with Vstm2 proteins using COS-7 cells and a cell-free pull-down assay (Figs. 1 and 2). To rigorously and quantitatively analyze the interaction between purified Nrxn2aSS2+ and Vstm2 proteins, it is important to analyze the interaction using a biophysical method such as SPR, ITC, or other. The same quantitative approach should be used to determine whether the SS2 insert acts as a switch or as a modulator of the interaction with Vstm2 proteins.

Response:

We performed surface plasmon resonance (SPR) experiments using a Biacore T100 system. Purified human VSTM2B protein (UniProt A6NLU5; residues Ala^{26}–Arg^{151}; C-terminal 6×His tag) and human NRXN2A N-terminal ectodomain fragments containing

the LNS1, EGF1, and LNS2 domains (UniProt Q9P2S2; residues Asp^{27}-Asp^{488}; C-terminal 6×His tag), with or without the SS2AB insert, were prepared. NRXN2A proteins with or without SS2AB were immobilized on the sensor chip surface. VSTM2B was injected as the analyte at varying concentrations. The SPR results show that VSTM2B binds specifically to NRXN2A N-terminal fragments containing the SS2AB insert, but not to those lacking SS2AB, consistent with our *in situ* binding assays. These SPR data have been included in the revised manuscript as Supplementary Figure 3.

2. Fig. 6: the VGLUT1 and VGAT puncta look unusually fuzzy and show high background, especially in panels 6G and 6I. It is not clear whether these puncta represent synaptic puncta. These experiments should be repeated, with better imaging quality. How do the authors explain the residual synapses on Vsmt2b-expressing cells co-cultured with Nrxn2a KO neurons (Fig. 6G, H)?

Response:

We repeated the artificial synapse formation assays using two independent approaches, Vstm2b-coated beads and HEK cells expressing HA-Vstm2b, to address the reviewer's concerns regarding image quality and the *Nrxn2α* KO effect. In these new experiments, single immunostaining for VGLUT1 or VGAT was performed on separate neuronal coverslips to eliminate any possibility of antibody cross-reactivity or emission-filter bleed-through.

First, in the bead-based assays, Vstm2b-Fc-coated beads robustly induced the accumulation of VGLUT1, but not VGAT, in wild-type hippocampal neurons. This selective induction of excitatory, but not inhibitory, presynaptic markers was independently reproduced using the HEK-cell-based artificial synapse formation assay. Based on these convergent results, we revised our conclusion to state that Vstm2b specifically promotes excitatory presynaptic assembly.

Second, bead-based assays performed with hippocampal neurons from *Nrxn2α* KO mice demonstrated a complete lack of Vstm2b-Fc-induced VGLUT1 accumulation (levels were equivalent to negative control levels). Thus, no residual synapse-inducing activity of Vstm2b was observed in the absence of *Nrxn2α*. These findings strongly support the conclusion that endogenous Nrxn2α is the sole functional receptor mediating Vstm2b-dependent synapse induction.

In accordance with these new data, we have revised Figure 6 in the manuscript and added Supplementary Figures 10 and 11 to present the updated experiments with improved imaging quality.

3. The subcellular fractionation suggests Vstm2b localizes to synaptic fractions, but biochemical analysis does not give a clear indication whether Vstm2b is postsynaptic at excitatory or inhibitory synapses. Given that little is known about Vstm2b and its role at synapses, the subcellular localization of endogenous Vstm2b at excitatory and inhibitory synapses needs to be demonstrated convincingly. If antibodies to Vstm2b are not suitable for detecting Vstm2b at the immunocyto- or histochemical level, the authors should perform CRISPR/Cas9 knock-in of an epitope tag to characterize subcellular localization.

Response:

We agree with the reviewer that the inability to directly visualize endogenous Vstm2b represents a limitation of the current study, as noted in the Discussion.

Although not described in detail in the manuscript, we undertook extensive and systematic efforts to determine the endogenous subcellular localization of Vstm2b.

First, we evaluated multiple commercially available antibodies and a custom-generated antibody across a wide range of fixation conditions and immunocytochemical and immunohistochemical protocols. None of these antibodies yielded specific or reliable signal at synaptic resolution. All validation experiments were performed in parallel on WT and *Vstm2b* KO neurons and tissues to rigorously assess specificity. No antibody produced a signal that was selectively absent in the KO condition, indicating insufficient specificity and/or sensitivity for endogenous detection.

As an alternative strategy, we attempted endogenous epitope tagging using CRISPR/Cas9. We generated N-terminal HA knock-in constructs using the pORANGE system in primary rat hippocampal neurons (ref 1). Importantly, we confirmed that N-terminal tagging does not impair Vstm2b trafficking to the cell surface or its ability to bind Nrxn2α and induce presynaptic assembly (Figure 4A–B and Figure 6), demonstrating that the tag does not disrupt known functional properties. Positive controls, including Tubulin and PSD scaffolding proteins, verified that the tagging and detection workflow was technically robust. However, in contrast to these controls, endogenous HA-Vstm2b signal remained below reliable detection thresholds. Given that synaptic adhesion molecules are frequently

expressed at low abundance, we consider limited endogenous expression the most likely explanation.

We also considered generating a CRISPR knock-in mouse line. While feasible, this approach would require at least six months before analysis could begin and still would not guarantee detectable signal. Indeed, we previously applied an analogous strategy to another GPI-anchored Nrxn2α-interacting postsynaptic adhesion molecule, IgSF21, inserting an HA tag into its membrane-proximal extracellular domain (ref 2). Although tagging did not disrupt surface targeting or binding to its presynaptic receptor Nrxn2α, endogenous IgSF21 remained undetectable in tissue sections despite successful genomic incorporation, again likely due to low expression. This precedent suggests that a knock-in approach may not overcome the sensitivity limitations for Vstm2b.

To further assess subcellular targeting within detectable limits, we expressed exogenous HA-Vstm2b at low-to-moderate levels in primary rat hippocampal neurons and quantified its axon-to-dendrite distribution. These experiments revealed that Vstm2b can target to both compartments without strong compartmental bias. These new data have been added to the revised manuscript as Supplementary Figure 9.

In summary, while we fully acknowledge the limitation imposed by the inability to visualize endogenous Vstm2b at defined synapse types, we have undertaken extensive, technically rigorous, and multifaceted efforts to address this question within the scope of the present study.

References

1. **Willems, J., de Jong, A. P. H., Scheefhals, N., Mertens, E., Catsburg, L. A. E., Poorthuis, R. B., de Winter, F., Verhaagen, J., Meye, F. J., & MacGillavry, H. D. (2020). *ORANGE: A CRISPR/Cas9-based genome editing toolbox for epitope tagging of endogenous proteins in neurons.* PLoS Biology, 18(4), e3000665.**
2. **Tanabe, Y., Naito, Y., Vasuta, C., Lee, A. K., Soumounou, Y., Linhoff, M. W., & Takahashi, H. (2017). *IgSF21 promotes differentiation of inhibitory synapses via binding to neurexin2α.* Nature Communications, 8, 408.**

4. I understand that the authors use a global KO mouse for Vstm2b for a first characterization of the role of this protein. However, this approach does not allow to draw direct conclusions about a

postsynaptic role of Vstm2b at excitatory synapses: there is a possibility that some of the observed phenotypes are indirect consequences of Vstm2b loss in the brain or reflect Nrxn-independent roles of Vstm2b. Conclusive evidence that Vstm2b is functionally required at excitatory postsynapses is required, ideally showing that this is Nrxn2a-dependent. If the authors have a Vstm2b floxed mouse, targeted deletion of Vstm2b will provide insight. In the absence of a floxed Vstm2b mouse, a rescue strategy with Vstm2b in the global Vstm2b KO could be an alternative approach. If the authors have identified Vstm2b mutations that prevent interaction with Nrxn2a (based on their model in Fig. 3), rescue with such Vstm2b mutants could be insightful.

Response:

Our laboratory does not currently maintain a *Vstm2b* floxed mouse line, nor have we yet identified a Vstm2b mutant that fully abolishes Nrxn2α binding in a manner suitable for the rescue experiments suggested by the reviewer.

To directly assess the postsynaptic requirement for Vstm2b, we instead performed mixed hippocampal neuron coculture experiments. We generated cultures containing 10% GFP-labeled (GFP$^+$) *Vstm2b* WT or KO neurons and 90% unlabeled WT or KO neurons, allowing us to isolate postsynaptic versus presynaptic contributions.

Postsynaptic involvement was evaluated by comparing dendritic spine density in GFP$^+$ WT and GFP$^+$ KO neurons cocultured with an excess of WT neurons. Conversely, presynaptic involvement was assessed by evaluating GFP$^+$ WT neurons cocultured with an excess of either WT or KO neurons.

When cocultured with an excess of WT neurons, GFP$^+$ KO neurons exhibited a significantly lower density of head-positive dendritic spines compared to GFP$^+$ WT neurons, indicating that loss of postsynaptic Vstm2b reduces dendritic spine density. In contrast, GFP$^+$ WT neurons showed no difference in spine density when cocultured with an excess of KO or WT neurons, suggesting that presynaptic Vstm2b does not contribute detectably to the spine density of postsynaptic target neurons.

Notably, head-negative protrusions (filopodia-like structures thought to represent immature spine precursors) were unchanged across all coculture conditions, implying that postsynaptic Vstm2b is required for spine maintenance rather than initial spine formation.

These new data support a postsynaptic role for Vstm2b in excitatory synapse maintenance, consistent with our *in vivo* evidence. We have added these results as Figure 6G-J in the revised manuscript.

In addition, as suggested by the reviewer, we are interested in performing targeted deletion of *Vstm2b* (e.g., using floxed mice combined with stereotactic injection of AAV expressing Cre into the ventral hippocampus) in a future study. To enable this approach, it is first necessary to establish synaptic phenotypes at the single-neuron level, because not all vCA1 pyramidal cells would be expected to be infected. Accordingly, we conducted patch-clamp recordings from vCA1 pyramidal neurons in *Vstm2b KO* and WT littermates of both sexes. Consistent with the female-specific phenotype observed in the fEPSP input–output relationship, mEPSC frequency was significantly reduced in KO females, but not in KO males, compared with WT mice. In the revised manuscript, we have included these new mEPSC data as Figure 8 and Supplementary Figure 18.

Minor Points

1. Line 536: "These findings are consistent with proteomic evidence identifying Vstm2a, but not Vstm2l or Vstm2b, in the neuronal mouse brain secretome" - The authors' data suggests that Vstm2l is not expressed on the surface, yet this is the family member that is identified in the Nrxn2a ECD AP-MS approach in Fig. 1. How do the authors explain this?

Response:

The Nrxn2α ECD AP-MS experiment used detergent-solubilized synaptosomes, which disrupt membranes and release intracellular components. Under these conditions, proteins not normally exposed at the cell surface, such as Vstm2l, can still be captured if they associate with Nrxn2α, likely explaining its identification in the assay.

Of the Vstm2 family members, Vstm2l binds Nrxn2α with the highest affinity, consistent with its preferential detection in the AP-MS screen. Although AP-MS is a powerful discovery method, it can miss low-abundance or low-affinity interactors. Follow-up biochemical and cell-based assays provided strong evidence that all three family members, Vstm2a, Vstm2b, and Vstm2l, interact with Nrxn2α via the LNS2 domain in an alternative-splicing-dependent manner.

The subcellular localization of Vstm2l remains unclear. Its ability to bind the extracellular domain of Nrxn2α suggests an extracellular role, yet neither our work nor previous studies have detected it at the cell surface or in the extracellular space. This raises the possibility that Vstm2l functions in a context-dependent manner, requires specific trafficking partners, or acts in a distinct intracellular compartment.

To clarify this point, we have revised the discussion section as follows:

Page 23, lines 655-659: "Notably, the initial identification of Vstm2l as an Nrxn2α binding partner was obtained from AP-MS experiments performed on detergent-solubilized synaptosomes, in which intracellular proteins are accessible for affinity purification due to membrane disruption, likely explaining its isolation despite the absence of detectable extracellular localization."

2. Bar graphs in Fig. 2 should be shown differently: indicating individual datapoints (as in e.g. Fig. 4) or as box-whisker plots.

Response:

We have replaced the bar graphs in Figures 2, 3 and Supplementary Figures 2 and 3 with box-and-whisker plots to better visualize data distribution and variability.

3. Why does the L360E Nrxn2a mutant enhance binding to Vstm2b?

Response:

At present, the structural basis for the enhanced binding of the L360E Nrxn2α mutant to Vstm2b is unclear. As noted in the manuscript (page 9, line 239-242), current AlphaFold predictions are unlikely to reliably capture the detailed interface for this interaction. Thus, definitive determination will require an experimental structure investigation of the Vstm2b-Nrxn2α LNS2 complex, using approaches such as X-ray crystallography and/or cryo-electron microscopy.

4. Fig 4A, B: what is the negative control in this experiment (e.g. untransfected cells)?

Response:

In these experiments, neighboring non-transfected cells, identified as DAPI-positive and myc-negative, served as internal negative controls for both the bound Nrxn2α-Fc signal and the myc-Vstm2a/b/l signal. In the revised manuscript, quantification of these negative controls has now been included in Figure 4A-C.

5. Fig. 4D: is this MS data from N=1 surface biotinylation experiment? Please indicate in the legend.

Response:

The MS data in Figure 4D were obtained from two biological replicates, corresponding to independent cortical neuron culture wells derived from the same batch of neurons. We have updated the figure legend to clarify the number and nature of replicates.

Page 62 line 1721-1724: ***"*(E) Exclusive spectrum counts of Vstm2a, Vstm2b, and selected cell adhesion molecules from the cortical neuron surfaceome (n = 2 biological replicates, independent culture wells from the same neuronal batch). The negative control, processed without biotin, accounts for non-specific binding and background signal.*"***

6. Fig. 4F: why is GAPDH detected in the culture medium?

Response:

GAPDH is classically considered a cytosolic glycolytic enzyme, but multiple studies have documented its presence outside cells. For example, GAPDH has been detected in the conditioned medium of several mammalian cultured cell lines, including COS-7, HEK293, HepG2, PC-12, and Neuro-2a, and in rat serum, where extracellular GAPDH has been shown to inhibit cell spreading, supporting a physiological function independent of cell lysis (ref 1).

Furthermore, GAPDH has been reported as a component associated with extracellular vesicles, binding to the EV surface through a phosphatidylserine-binding motif, consistent with a non-classical extracellular pool of GAPDH (ref 2).

References

1. **Yamaji, R., Chatani, E., Harada, N., Sugimoto, K., Inui, H., & Nakano, Y. (2005). Glyceraldehyde-3-phosphate dehydrogenase in the extracellular space inhibits cell spreading. *Biochimica et Biophysica Acta (BBA) - General Subjects, 1726*(3), 261–271. https://doi.org/10.1016/j.bbagen.2005.09.003**

2. **Dar, G. H., Mendes, C. C., Kuan, W.-L., Speciale, A. A., Conceição, M., Görgens, A., Uliyakina, I., Lobo, M. J., Lim, W. F., El Andaloussi, S., Mäger, I., Roberts, T. C., Barker, R. A., Goberdhan, D. C. I., Wilson, C., & Wood, M. J. A. (2021). GAPDH**

controls extracellular vesicle biogenesis and enhances the therapeutic potential of EV mediated siRNA delivery to the brain. *Nature Communications, 12*(1), 6666. https://doi.org/10.1038/s41467-021-27056-3

7. Fig. 5E, F: Vstm2b immunoreactivity on western blot shows multiple bands. The green arrowhead points to the correct band, but it is not clear whether only the upper band is considered to represent Vstm2b, or also the lower band. How did the authors determine that these bands indeed represent Vstm2b? Was this with the blot shown in Fig. S6D? If so, this should be indicated at this point in the text.

Response:

To assess antibody specificity, we compared WT and Vstm2b KO brain lysates by western blot (Sup. Fig. 7D). The two ~37 kDa bands seen in Fig. 5F were absent in the KO lysate, confirming they represent Vstm2b. In contrast, the ~50 kDa band observed in Fig. 5E remained in the KO sample (Sup. Fig. 7D), indicating it is non-specific.

To prevent confusion, we removed the green arrowheads in Fig. 5E and F and added an asterisk at ~50 kDa in Sup. Fig. 7D. The Figure 5 legend now states that this asterisk marks a non-specific band validated in Sup. Fig. 7D.

8. Fig. 6L: the IgG-coated control beads are not touching neurites in the example image shown. How does this look when they do touch neurites? Do Vstm2b-coupled beads induce VGLUT1 clustering in Nrxn2a KO cultures?

Response:

The two beads at the bottom left of the Fc-coated bead image are indeed in contact with neurites, as shown in the revised Supplementary Fig. 12B. In the updated figure, we replaced the inset with a more representative region and enhanced the MAP2 signal intensity to improve neurite visualization. This revised presentation more clearly highlights the contrast between Fc-coated beads non-specifically associated with neurites and Vstm2b-coated beads specifically attached to them.

As shown in the revised Fig. 6E, F and Supplementary Fig. 10, Vstm2b-Fc-coated beads robustly induce the accumulation of VGLUT1, but not VGAT, around beads contacting

axons of WT mouse hippocampal neurons, whereas this effect is absent in *Nrxn2α*-deficient neurons.

9. Supplementary Fig. 7: "… did not detect Nrxn2β-specific-peptides (Supplementary Fig. 7)". It is not entirely clear how this was concluded: the figure shows common sequences between Nrxn2a and Nrn2b as underlined but sequence unique to Nrxn2b is not shown: do the red sequences shown represent all identified peptides?

Response:

The red sequences in the revised Supplementary Fig. 12 represent all peptides identified by LC-MS/MS in the pulldown experiments. No Nrxn2β-specific peptides were detected, supporting our conclusion that Nrxn2β was not pulled down. The figure legend has been revised to clarify that the red sequences include all detected peptides and that none were unique to Nrxn2β.

Updated figure legend for Supplementary Fig. 12F:

"(F) Amino acid sequences of rat Nrxn2α peptides (red) identified by LC-MS/MS following pulldown of Vstm2b and associated protein complexes. The sequences underlined are shared between Nrxn2α and Nrxn2β isoforms. The red sequences represent all peptides detected in the experiment; no Nrxn2β-specific peptides were identified."

10. Have the authors analyzed effects of Vmst2b KO on inhibitory synapse density or synaptic transmission?

Response:

We performed immunohistochemistry for VGAT and gephyrin and quantified their co-puncta, which represent inhibitory synapses, in the *stratum pyramidale* (SP), *stratum radiatum* (SR) and *stratum lacunosum moleculare* (SLM) of the vCA1 in *Vstm2b* knockout (KO) and wild-type (WT) mice of both sexes. We found no significant differences in inhibitory synapse number in either the vCA1 SP or SLM in either sex, indicating that Vstm2b primarily regulates excitatory, rather than inhibitory, synapses. A description of this result has been added to the revised Results section, and the data are included as Supplementary Figure 16.

11. Line 450: "… the Vstm2b-Nrxn2a complex emerges as a key regulator of ventral hippocampal glutamatergic circuits, providing a mechanism for sexually dimorphic synapse specificity and circuit function." This conclusion seems a bit overstated; the mechanism underlying the sexual dimorphic effects remains unclear. Are there differences in Vstm2b expression patterns in male vs. female mice?

Response:

We have tempered the statement in the revised manuscript.

Page 19, line 538-540: "Together, these findings indicate that the Vstm2b-Nrxn2α complex emerges as an important regulator of ventral hippocampal glutamatergic circuits and may contribute to sexually dimorphic synapse specificity and circuit function."

12. Small typo: Fig. 1E legend: 'pull-downed'; line 440: a period seems to be missing between 'LNS2 domain' and 'Vmst2b'.

Response:

The identified typographical errors have been corrected as follows:

Page 56, Fig. 1E legend: "(E) Immunoblots of purified human Vstm2a-His (left), Vstm2b-His (middle) and HA-Vstm2l (right) pulled down by purified human Nrxn2α-Fc (SS1+, SS2A+, SS3−, SS4−, SS5−) but not by Fc alone."

Page 18-19, line 526-529, Discussion: "Our proteomic profiling of the Nrxn2α ECD interactome revealed Vstm2a, 2b, and 2l as novel α-Nrxn-specific ligands, with interaction requiring inclusion of an insert at SS2 within the LNS2 domain. Vstm2b selectively interacts with Nrxn2α^{SS2+} via a binding site that overlaps with that of Nxph1, resulting in direct binding competition."

Authors' response to the reviewers' comments for the manuscript **NCOMMS-26-000546A** of Chofflet N. et al., **"Sexually Dimorphic Control of Excitatory Synapse Maintenance in Ventral Hippocampal Circuits by Neurexin2α Ligand Vstm2b"**, and description of changes made in the revised paper

We would like to thank all reviewers for their positive evaluation of our manuscript. We have addressed the remaining minor comments, as detailed below.

Reviewer #3 (Remarks to the Author):

In reviewing the revisions, I noted the following minor issues that the authors can easily correct in the final version of their manuscript:

-legend Fig. S3 contains a small typo: 'surface plasma' should be 'surface plasmon'; in addition, the labels in the graphs should be changed from 'um' to 'uM'

Response:

We thank the reviewer for this careful observation. In Supplementary Figure 3, the typographical error has been corrected from "surface plasma" to "surface plasmon." In addition, all concentration labels in the figure have been revised from "μm" to "μM."

-response to minor point #5: 2 independent culture wells from the same neuronal culture in my view should be represented in the figure legend as 2 technical replicates, not 2 biological replicates. For biological replicates, I would consider 2 independently prepared neuronal cultures to represent biological replicates.

Response:

We thank the reviewer for this important clarification. In the experiment shown in Fig. 6G-J, data were collected from two independently prepared neuronal cultures. To avoid ambiguity, we have revised the figure legend to state:

"Data were obtained from two independently prepared neuronal cultures."
